# Lifestyle Interventions, Kidney Disease Progression, and Quality of Life: A Systematic Review and Meta-analysis

**DOI:** 10.1016/j.xkme.2023.100643

**Published:** 2023-04-18

**Authors:** Elizabeth P. Neale, Vinicius Do Rosario, Yasmine Probst, Eleanor Beck, Thai Binh Tran, Kelly Lambert

**Affiliations:** School of Medical, Indigenous and Health Science, University of Wollongong, Australia

**Keywords:** Diet, lifestyle exercise, physical activity, systematic review, meta-analysis

## Abstract

**Rationale & Objective:**

Poor dietary patterns and low physical activity levels are important lifestyle-related factors that contribute to negative health outcomes in individuals with chronic kidney disease (CKD). Previous systematic reviews have not explicitly focused on these lifestyle factors, nor undertaken meta-analyses of any effects. We aimed to evaluate the effect of lifestyle interventions (such as diet, exercise, and other lifestyle-related interventions) on the risk factors for and progression of CKD and the quality of life.

**Study Design:**

Systematic review and meta-analysis

**Setting & Study Populations:**

Individuals aged 16 years or older with CKD stages 1 to 5 not requiring kidney replacement therapy.

**Selection Criteria for Studies:**

Randomized controlled trials of interventions.

**Data Extraction:**

Kidney function, albuminuria, creatinine, systolic blood pressure, diastolic blood pressure, body weight, glucose control, and quality of life.

**Analytical Approach:**

A random-effects meta-analysis with evidence certainty assessed using GRADE.

**Results:**

Seventy-eight records describing 68 studies were included. Twenty-four studies (35%) were dietary interventions, 23 (34%) exercise, 9 (13%) behavioral, 1 (2%) hydration, and 11 (16%) multiple component. Lifestyle interventions resulted in significant improvements in creatinine (weighted mean difference [WMD], −0.43 mg/dL; 95% CI, −0.74 to −0.11; *P* = 0.008); 24-hour albuminuria (WMD, −53 mg/24 h; 95% CI, −56 to −50; *P* < 0.001); systolic blood pressure (WMD, −4.5 mm Hg; 95% CI, −6.7 to −2.4; *P* < 0.001); diastolic blood pressure (WMD, −2.2 mm Hg; 95% CI, −3.7 to −0.8; *P* = 0.003); and body weight (WMD, −1.1 kg; 95% CI, −2.0 to −0.1; *P* = 0.025). Lifestyle interventions did not result in significant changes in the estimated glomerular filtration rate (0.9 mL/min/1.73 m^2^; 95% CI, −0.6 to 2.3; *P* = 0.251). However, narrative synthesis indicated that lifestyle intervention resulted in improvements in the quality of life.

**Limitations:**

Certainty of the evidence was rated very low for most outcomes, primarily owing to the risk of bias and inconsistency. No meta-analysis was possible for quality-of-life outcomes because of variations in measurement tools.

**Conclusions:**

Lifestyle interventions seem to positively affect some risk factors for progression of CKD and quality of life.

**Plain-Language Summary**

Lifestyle interventions, such as those involving changes to diet and exercise, may improve the risk factors for and progression of chronic kidney disease (CKD) and the quality of life in people with CKD. We conducted a systematic review and meta-analysis to examine the current evidence base on lifestyle interventions and CKD. We found 68 randomized controlled trials, 24 being dietary interventions and 23 being exercise interventions. When combined, lifestyle interventions resulted in significant improvements in creatinine, 24-hour albuminuria, systolic blood pressure, diastolic blood pressure, and body weight, although not the estimated glomerular filtration rate. Quality of life improved after lifestyle interventions. Lifestyle interventions seem to positively affect some risk factors for progression of CKD and quality of life.

The burden of chronic kidney disease (CKD) is increasing globally and, in 2019, was ranked as the 18th leading cause of global disability-adjusted life years.^1^ Strategies to prevent the development and progression of CKD are important. Lifestyle-related factors, such as increasing intake of vegetables, increasing physical activity, reducing salt intake, and moderating alcohol consumption are associated with primary prevention of CKD.^2^ In a systematic review of 26 studies of lifestyle interventions (such as diet, physical activity, or general support for people with CKD), more than two-thirds (69%) of studies showed an improvement in at least 1 primary outcome.^3^ However, the effects on progression and quality of life (QoL) are yet to be synthesized fully. We sought to undertake a systematic review of lifestyle interventions on the risk factors for and progression of chronic kidney disease and the QoL in people with CKD.

## Methods

This systematic review is reported according to the Preferred Reporting Items for Systematic Reviews and Meta-analyses checklist^4^ (Item S1). The review protocol was prospectively registered in the International Prospective Register of Systematic Reviews (http://www.crd.york.ac.uk/PROSPERO, registration number: CRD42017082079). Because this study involved synthesis of existing data, informed consent was not required.

### Study Eligibility

The study eligibility criteria are listed in Table 1. Where studies included a mixture of eligible and noneligible participants (eg, adults and children), these studies were included only if the data could be extracted for the eligible group. Studies were restricted to those published in English.

**Table 1:** Inclusion and Exclusion Criteria

	Inclusion Criteria	Exclusion Criteria
Population	Participants aged ≥16 years with CKD	Individuals undergoing kidney replacement therapy or palliative care, and/or pregnant or breastfeeding individuals
Intervention	Interventions conducted in the outpatient setting, which explored the effect of diet, physical activity, exercise, or combined lifestyle (diet, physical activity, and/or exercise) interventions	Medication-only interventions
Comparator	Allowed for the effect of the intervention to be isolated	—
Outcome	Reported the following outcomes: progression of CKD (as indicated by GFR, eGFR, albuminuria, proteinuria, or serum creatinine), or risk factors of progression of existing CKD, such as systolic blood pressure, diastolic blood pressure, body weight, and HbA_1c_; or QoL	—
Study Design	Randomized controlled trials	All other study designs

Abbreviations: CKD, chronic kidney disease; GFR, glomerular filtration rate; eGFR, estimated glomerular filtration rate; HbA_1c_, hemoglobin A_1c_, QoL, quality of life.

### Information Sources and Study Selection

A systematic search of the databases MEDLINE (EBSCOhost), PubMed, Cumulative Index to Nursing and Allied Health Literature (EBSCOhost), and Cochrane Central Register of Controlled Trials was conducted by EPN up to December 8, 2022. No date restrictions were applied. MEDLINE was searched using both EBSCOhost and PubMed to ensure the most recent articles were obtained, as recommended by Rosen and Suhami.^5^ A combination of free-text terms and Medical Subject Headings terms were used.^5^ Search strategies for all databases are shown in Item S2. The search strategy for MEDLINE, PubMed, and Cumulative Index to Nursing and Allied Health Literature incorporated the Cochrane Highly Sensitive Search Strategy for identifying randomized trials.^6^

Records were initially managed in Endnote version 20 (2020; Endnote 20 [software]) for removal of the duplicates. The automation tool Abstrackr was used for the screening of title and abstracts.^7^ Then, full-text articles were retrieved for the assessment of eligibility. Title and abstract screening and full-text review were conducted in duplicate by 2 independent researchers, with disagreements resolved by discussion until consensus was reached. Where multiple records from the same study were found, all were included but linked to the same study if they reported different outcomes. If the same outcomes were reported, the record reporting the longest duration was included.

### Data Collection and Summary Measures

The following data were extracted from eligible studies: country; sample size used for the analysis; participant age, body mass index, CKD stage, and comorbid conditions; and study design, duration, type of intervention, details of the intervention and control arms, and study results. Further details regarding the data extraction methods are provided in Item S3.

### Risk of Bias

When the review commenced, the risk of bias was assessed using the most recent version of the Cochrane Risk of Bias tool 1.0.^8^ Data extraction and risk-of-bias assessment were conducted in duplicate by 2 independent researchers, with conflicts discussed until consensus was reached. Then, extracted data and risk-of-bias assessment was checked again by EPN, and any variation was confirmed with the original study.

### Synthesis of Results

Random-effects meta-analyses were conducted using Stata IC (version 15.1), using the metan command (using the randomi option for random effects). This command uses the DerSimonian and Laird method with the heterogeneity estimate taken from the inverse-variance fixed-effects model.^9^^,^^10^ The weighted mean differences (with 95% confidence interval [CI]) in change or the final mean values for each outcome were calculated.

Prespecified subgroup analyses were conducted based on the type of intervention (exercise, diet, behavior, hydration, or multiple interventions). Although prespecified subgroup analyses based on the CKD stage and comorbid conditions were planned, these were not possible owing to substantial overlap in the subgroups among the studies. Further details regarding the data synthesis methods are provided in Item S4.

The proportion of total variation attributable to the between-study heterogeneity was estimated using the I^2^ statistic.^11^ Contour funnel plots were generated to explore the presence of small study effects for outcomes with 10 or more effect sizes.^12^ The Egger test was used to examine the extent of funnel plot asymmetry.^13^ In the case of funnel plot asymmetry, sensitivity analyses using the trim-and-fill method were conducted to explore these findings further.

The meta-analysis was not appropriate for the QoL owing to the substantial variation in the tools and domains reported among the studies. Thus, the narrative synthesis was used for the QoL, with vote counting used to synthesize the findings, based on whether there were significant improvements in QoL for intervention compared with those of the control, nonsignificant improvements, no effect, significant reductions, or nonsignificant reductions.

### Certainty of the Body of Evidence

The certainty of the body of evidence was assessed using GRADE^14^ software (GRADEpro GDT: GRADEpro Guideline Development Tool; McMaster University, 2015; developed by Evidence Prime Inc; www.gradepro.org). In the case of outcomes that were pooled using a meta-analysis, studies that could not be included in the meta-analysis were not formally included in the pooled GRADE assessment, but their potential effect on the GRADE assessment was considered.

## Results

**Figure 1:**
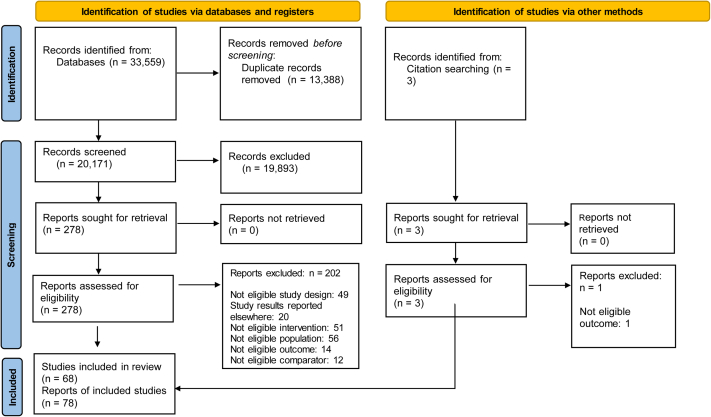
PRISMA flow diagram of study selection.

Across the original and updated searches, 33,559 records were found (Fig 1). After the removal of the duplicates, 20,171 records were screened, with an additional record identified from citation searching. This resulted in 278 full-text records assessed for eligibility, with 78 records describing 68 studies included in the review.

### Study Characteristics

The characteristics of the included studies are outlined in Table 2, Table 3, Table 4, Table 5.^15^, ^16^, ^17^, ^18^, ^19^, ^20^, ^21^, ^22^, ^23^, ^24^, ^25^, ^26^, ^27^, ^28^, ^29^, ^30^, ^31^, ^32^, ^33^, ^34^, ^35^, ^36^, ^37^, ^38^, ^39^, ^40^, ^41^, ^42^, ^43^, ^44^, ^45^, ^46^, ^47^, ^48^, ^49^, ^50^, ^51^, ^52^, ^53^, ^54^, ^55^, ^56^, ^57^, ^58^, ^59^, ^60^, ^61^, ^62^, ^63^, ^64^, ^65^, ^66^, ^67^, ^68^, ^69^, ^70^, ^71^, ^72^, ^73^, ^74^, ^75^, ^76^, ^77^, ^78^, ^79^, ^80^, ^81^, ^82^, ^83^, ^84^, ^85^, ^86^, ^87^, ^88^, ^89^, ^90^, ^91^, ^92^ Studies were predominantly of parallel design, with 5 studies following a crossover design^16^^,^^65^^,^^69^, ^70^, ^71^, ^72^ and 3 studies following a cluster randomized design.^16^^,^^57^^,^^89^^,^^92^ Duration of the interventions ranged from 1 week^65^ to 5 years.^52^ A range of CKD stages were investigated within the studies, with the most common stages being stages 3-4. Dietary interventions were investigated in 24 studies,^46^, ^47^, ^48^^,^^50^, ^51^, ^52^, ^53^, ^54^, ^55^, ^56^, ^57^, ^58^, ^59^, ^60^, ^61^, ^62^, ^63^, ^64^, ^65^, ^66^, ^67^, ^68^, ^69^, ^70^, ^71^, ^72^ with exercise interventions used in 23 studies.^15^, ^16^, ^17^, ^18^, ^19^, ^20^, ^21^, ^22^, ^23^, ^24^, ^25^, ^26^, ^27^, ^28^, ^29^, ^30^, ^31^, ^32^, ^33^, ^34^, ^35^, ^36^, ^37^, ^38^, ^39^, ^40^, ^41^, ^42^, ^43^, ^44^, ^45^ Eleven studies assessed interventions involving multiple components (eg, both diet and exercise),^16^^,^^82^, ^83^, ^84^, ^85^, ^86^, ^87^, ^88^, ^89^, ^90^, ^91^, ^92^ whereas 9 studies involved behavioral interventions,^73^, ^74^, ^75^, ^76^, ^77^, ^78^, ^79^, ^80^, ^81^ and 1 study implemented an intervention focused on changing the participants’ water intake alone.^49^ Further details of the interventions are shown in Item S5.

**Table 2:** Characteristics of Included Studies Assessing the Effect of Exercise Lifestyle Interventions on CKD Progression

Study, Country	Sample Size (for Analysis)	Age (y)	BMI (kg/m^2^)	CKD Stage	Comorbid Conditions	Design	Study Duration (wk)^a^
Aoike (2015), Brazil^15^	29	55.1 ± 11.6	31.2 ± 6.1	3-4	T2DM (∼45% of sample)	P	12
Aoike (2018)/Gomes (2017),Brazil^16^^,^^17^	40	55.8 ± 8.3	31.2 ± 4.4	3-4	T2DM (∼35% of sample)	P	24
Barcellos (2018), Brazil^18^	109	C: 65.1 (1.3); I: 65.0 (1.2)^b^	C: 30.1 (0.6); I: 29.7 (0.7)^b^	2-4	HT	P	16
Baria (2014), Brazil^19^	27	52.1 ± 9.5	30.4 ± 3.8	3-4	T2DM (∼22% of sample)	P	12
Corrêa (2021)/Corrêa (2021)/de Deus (2021)/de Deus (2022), Brazil^20^, ^21^, ^22^, ^23^	105^c^; 90^d^	C: 58 ± 5; I1: 58 ± 6; I2: 58 ± 7	C: 33.2 ± 1.6; I1: 33.6 ± 2.0; I2: 33.3 ± 1.9	2	HT and T2DM	P	24
Castaneda (2001), United States^24^	26	C: 64 ± 13; I: 65 ± 9	C: 26.8 ± 2.7; I: 29.3 ± 6.6	Serum creatinine concentrations, 1.5-5.0 mg/dL	Diagnosed HT: control: 83%, intervention: 64%; mean number of chronic conditions: C: 6.4 ± 1.7, I: 5.5 ± 1.7	P	12
Eidemak (1997), Denmark^25^	30	C: 44 (28-66); I: 45 (22-70)^e^	NR	eGFR median 25 (range 10-43) mL/min/1.73 m^2^	NR	P	Mean follow-up time: C: 20 mo; I: 18 mo
Grazioli (2022), Italy^26^	21	62.7 ± 5.0	C1: 28.9 ± 3.0; C2: 27.3 ± 3.3; I1: 28.3 ± 4.5; I2: 25.5 ± 1.8	1-3b	NR	P	12
Greenwood (2014), UK^27^	18	C: 53.3 ± 12.9; I: 53.8 ± 13.5	C: 28.44 ± 4.24; I: 27.40 ± 3.52	3-4	Mixed	P	12 mo
Headley (2014)/Headley (2017)/Miele (2017), United States^28^, ^29^, ^30^	46	C: 57.1 ± 9.0; I: 58.0 ± 8.0	C: 36.5 ± 8.9; I: 34.9 ± 8.0	3	T2DM or HT	P	16
Hiraki (2017), Japan^31^	28	68.7 ± 6.8	23.7 ± 3.1	3-4	Mixed	P	12 mo
Kirkman (2019)/Kirkman (2021), United States^32^^,^^33^	31^d^; 26^f^	C: 62 ± 9; I: 55 ± 13	C: 34 ± 6; I: 30 ± 2	3-5	NR	P	12
Leehey (2009), United States^34^	11	66 (55-81)^e^	BMI ≥ 30	2-4	T2DM and obesity	P	24
Leehey (2016), United States^35^	32	66 ± 8.0^g^	37 ± 4.5^g^	2-4	T2DM and obesity	P	12 mo
Mustata (2011), Canada^36^	20	C: 72.5 (59-79); I: 64 (55-73)^h^	C: 29 (25-30); I: 27.5 (25-32)^h^	3-4	Diabetic cause of CKD (55%)	P	12 mo
Otobe (2021), Japan^37^	44	C: 78.1 ± 7.4; I: 78.4 ± 6.4^h^	C: 24.1 ± 3.7; I: 23.8 ± 4.1^h^	3-4	Cerebrovascular disease (9.4%), ischemic heart disease (7.5%), diabetes (20.8%), HT (79.2%), dyslipidemia (54.7%), neurologic disorder (1.9%), and orthopedic disease (15.1%)	P	24
Rahimimoghadam (2018), Iran^38^	50	C: 52.11 ± 11.4; I: 49.12 ± 10.3	NR	2-3	NR	P	12
Rossi (2014), United States^39^	94	C:67.7 ± 12.4; I: 69.2 ± 12.4	C: 32.2 ± 7.3; I: 30.7 ± 8.7	3-4	Diabetes (41%) and coronary artery disease (25%)	P	12
Shi (2014), China^40^	21	69.4 ± 7.7	NR	NR	CVD (100%), diabetes (33%), HT (71%), and hyperlipidemia (57%)	P	12
Tang (2017), China^41^	84	C: 43.90 ± 12.44; I: 46.26 ± 15.61	C: 23.30 ± 3.18; I: 23.82 ± 3.76	1-3	45.2% with ≥ 1 comorbid condition	P	12
Thompson (2022), Canada^42^	44	69 (56-73)^h^	32 (27-35)^h^	eGFR 15-44 mL/min/1.73^2^	Chronic heart failure (2.3%), peripheral vascular disease (4.5%), stroke (11.4%), diabetes (54.5%), cancer (20.5%), and depression/anxiety (18.2%)	P	24
Uchiyama (2021)/Adachi (2022), Japan^43^^,^^44^	46	73 (69-78)^h^	23.9 ± 4.5	4	Diabetes (30%) and cerebrovascular/cardiovascular disease (26%)	P	24
Van Craenenbroeck (2015), Belgium^45^	40	C: 54.7 ± 14.1; I: 51.5 ± 11.8	C: 28.3 ± 5.8; I: 28.3 ± 6.2	3-4	Diabetes (10%)	P	12

Abbreviations: BMI, body mass index; C, control; HT, hypertension; I, intervention; NR, not reported; P, parallel; T1DM, type 1 diabetes mellitus; T2DM, type 2 diabetes mellitus.

a Duration reported in weeks (using 4 wk/mo) for duration of <12 months and reported as months/years for duration of 12 months and more.

b Mean (standard error).

c For GFR, creatinine, body weight, and HbA_1c_.

d For blood pressure.

e Mean (range).

f For eGFR, systolic blood pressure.

g Characteristics reported for randomly assigned participants.

h Median (interquartile range).

**Table 3:** Characteristics of the Included Studies Assessing the Effect of Dietary Lifestyle Interventions on CKD Progression

Study, Country	Sample Size (for Analysis)	Age (y)	BMI (kg/m^2^)	CKD Stage	Comorbid Conditions	Design	Study Duration (wk)^a^	
Caldiroli (2022), Italy^46^	27	81 ± 6^b^	27.3 ± 6.5^b^	eGFR: >10 to <30 ml/min/1.73 ^2^	Diabetes (40%), HT (94%), and previous cardiovascular events (46%)	P	24	
Campbell (2008), Australia^47^	47	C: 68.5 ± 12.0; I: 71.0 ± 12.3	C: 27.0 ± 4.9; I: 27.4 ± 5.3	4-5	NR	P	12	
Chilelli (2015), Italy^48^	26	C: 65.2 ± 8.3; I: 64.3 ± 15.6	C: 25.28 ± 1.14; I: 24.86 ± 0.67	3-4	NR	P	12	
Clark (2013), Canada^49^^,^^c^	28	C: 67 ± 11; I: 59 ± 14	C: 30 ± 6; I: 31 ± 6	3	Hypertension—C: 100%, I: 77%; Hyperlipidemia—C: 73%, I: 53%; Diabetes—C 64%, I: 47%	P	6	
de Brito-Ashurst (2013), UK^50^	48	C: 60.7 ± 12.0; I: 55.7 ± 15.1	C: 27.1 ± 5.2; I: 26.6 ± 5.4	Moderate to severe: eGFR < 60 mL/min/1.73 m^2^	Mean BP >130/80 mm Hg	P	24	
Facchini (2003), United States^51^	170	C: 60 ± 12; I: 59 ± 10	C: 28 ± 5; I: 28 ± 5	Various degrees of kidney failure (GFR, 15-75 ml/min/1.73 m^2^) and unexplained proteinuria	T2DM	P	Mean follow-up: 3.9 y	
Goraya (2014)/Goraya (2019), United States^52^^,^^53^	72^d^; 66^e^^,^^f^	C: 53.9 ± 4.8; I: 53.5 ± 5.2	C: 28.2 ± 2.1; I: 28.8 ± 2.1	3	HT	P	60 m^e^; 36 mo^d^	
Hamidianshirazi (2022), Iran^54^	105	C: 49.4 (1.8); I: 50.1 (1.9)^b^^,^^g^	C: 26.7 (0.6); I: 26 (0.6)^b^^,^^g^	3-4	Participants did not present with diabetes, cancer, or heart failure	P	24	
Hwang (2014), South Korea^55^	245	49.5 ± 13.3	67.8 ± 13.5^h^^,^^i^	eGFR ≥ 30 ml/min per 1.73 m^2^	HT	Parallel	8	
Ihle (1989), Australia^56^	64	C: 36.8 ± 4.8; I: 37.2 ± 5.7	NR	Serum creatinine concentrations between 350 and 1,000 μmol/L	NR	P	18 mo	
Kankarn (2019a), Thailand^57^	172	C: 69.24 ± 7.70; I: 70.16 ± 8.79	C: 25.34 ± 25.34; I: 25.31 ± 3.77	3-4	Diabetes (10.5%), HT (25%), diabetes with HT (47.1%)	P (cluster)	12 mo	
Kelly (2020), Australia^58^	76	C: 61 ± 13; I: 63 ± 12	C: 31 ± 6; I: 33 ± 7	3-4	Diabetes (38.8%), CVD (32.5%), HT (81.3%)	P	24	
Martínez-Villaescusa (2022), Spain^59^	57	56.9^b^	C: 27.9; I: 26.3	4-5	HT (94.7%), dyslipidemia (81.3%), diabetes (25.3%), peripheral vascular disease (10.7%), cerebrovascular disease (1.3%), and ischemic heart disease (10.7%)	P	12 mo	
MDRD (Tangri (2011)/Kopple (1997), United States^60^^,^^61^	553^d^; 302^i^	C: 52.5 ± 12.2; I: 51.8 ± 12.1	NR	eGFR: 25 to 55 mL/min/1.73 m^2^	T2DM (3%)	P	2 y	
Mekki (2010), Algeria^62^	40	61 ± 14	26.2 ± 5.6	Moderate CKD (eGFR, 60-89 mL/min/1.73 m^2^)	Dyslipidemia	P	12	
Meloni (2002), Italy^63^	69	54.4 ± 15.3	NR	NR, diabetic nephropathy	T1DM (45%), T2DM (54%), and HT (100%)	P	12 mo	
Meloni (2004), Italy^64^	169	57.4 ± 17.8	NR	NR (n = 80 with diabetic nephropathy)	T1DM (14%), T2DM (33%), and HT (100%)	P	12 mo	
Moe (2011), United States^65^	8	61 ± 8.4	32 ± 5	3-4	Diabetes (50%) and HT (75%)	X	1	
Mozaffari-Rad (2022), Iran^66^	71	C: 63.51 ± 9.34; I: 53.87 ± 13.98	C: 29.94 ± 5.64; I: 27.64 ± 4.82	Protein to creatinine ratio >30 mg/g in a random urine sample and an eGFR >15 mL/min/1.73 m^2^ (eGFR range: 14.7-91.8 mL/min/1.73 m^2^)	Diabetes (49.2%) and HT (31%)	P	8	
Paes-Barreto (2013), Brazil^67^	89	63.4 ± 40.8	C: 28.3 ± 5.3; I: 28.9 ± 5.6	3-5	Diabetes (42.7%) and HT (92%)	P	16	
Sánchez (2009), Spain^68^	40	54 ± 13	C: 28.20 ± 7.06; I: 27.38 ± 5.4	Serum creatinine concentration >25 mg/dL	NR	P	12 mo	
Saran (2017), United States^69^	58	61^j^	NR	3-4	Diabetes (43%) and HT (93%)	X	4	
Slagman (2011), Netherlands^70^	52	Treatment sequence 1: 53 (3); treatment sequence 2: 55 (3); treatment sequence 3: 51 (4); treatment sequence 4: 47 (4)^g^	Treatment sequence 1: 27 (1); treatment sequence 2: 28 (1); treatment sequence 3: 28 (1); treatment sequence 4: 28 (1)^g^	NR	Nondiabetic nephropathy	X	6	
Soroka (1998), Israel^71^	9	30-85^k^	NR	creatinine clearance between 15 and 50 mL/min per 1.73 m^2^	HT	X	24	
Turban (2021), United States^72^	25	67.2 ± 11.6^b^	31.4 ± 4.7^b^	3	Medication for: diabetes (24.1%), HT (93.1%)^b^	X	4	

Abbreviations: BMI, body mass index; C, control; HT, hypertension; I, intervention; MDRD, Modification of Diet in Renal Disease; NR, not reported; P, parallel; T1DM, type 1 diabetes mellitus; T2DM, type 2 diabetes mellitus; X, crossover.

a Duration reported in weeks (using 4 wk/mo) for duration of <12 months and reported as months/years for duration of 12 months and more.

b Characteristics reported for randomly assigned participants.

c Study intervention was hydration related, not diet related.

d For body weight.

e For eGFR and systolic blood pressure.

f Study included other intervention group(s), which was not relevant to this review; therefore, this group(s) was not included in this analysis.

g Mean (standard error).

h Body weight (kg) is reported when BMI was not available.

i For GFR, creatinine, body weight, and HbA_1c_.

j Mean.

k Range.

**Table 4:** Characteristics of Included Studies Assessing the Effect of Behavioral Lifestyle Interventions on CKD Progression

Study, Country	Sample Size (for Analysis)	Age (y)	BMI (kg/m^2)^	CKD Stage	Comorbid Conditions	Design	Study Duration (wk)^a^
Joboshi (2016), Japan^73^	61	C: 70.1 ± 11.1; I: 67.0 ± 11.5	NR	1-5	Diabetes (∼46%)	P	12
Lin (2021), China^74^	108	64.40 ± 11.40	25.64 ± 4.19	1-3a	Diabetes (41.7%), HT (72.2%), heart disease (26.9%), and hyperlipidemia (55.6%)	P	6
Nguyen (2018), Vietnam^75^	135	C: 48.9 ± 13.9; I: 48.8 ± 13.7	C: 21.50 ± 2.65; I: 22.02 ± 3.38	3-5	3 comorbid conditions: 40.05%; 4 comorbid conditions: 48.9%; ≥5 comorbid conditions: 11.05%	P	12-wk intervention (follow-up at 16 wk)
Sevick (2012), United States^76^	32^b^	NR^b^	NR^b^	eGFR < 60 mL/min/1.73 m^2^	T2DM (100%)	P	24
St. Jules (2022), United States^77^	97^c^	C: 65 ± 10; I: 64 ± 8^d^	C: 34.4 ± 5.5; I: 33.2 ± 4.4^d^	1-4	All patients presented with T2DM	P	24
Teng (2021), Taiwan^78^	103	58.30 ± 11.17	28.79 ± 3.63	1-3	NR	P	30 mo
Tuot (2019), United States^79^	122	58.0 [50.0-64.0]^d^^,e^	NR	1-4	Diabetes (58.4%); coronary disease (15.3%); and hyperlipidemia (54%)	P	12 mo
Williams (2012), Australia^80^	75	67.0 ± 9.6^d^	C: 31.4 ± 5.9; I: 31.8 ± 5.4^d^	eGFR)>15 (≤60 mL/min/1.73 m^2^) or diabetic kidney disease (microalbumin/creatinine ratios > 2.0 mg/mmol for men, >3.5 mg/mmol for women)	T1DM and T2DM	P	12
Wu (2018), Taiwan^81^	90	C: 71.73 ± 12.68; I: 67.82 ± 9.43	NR	3b-5	HT (82.9%), high blood sugar (57.65%), high cholesterol (35.7%), and high triglycerides (34.95%)	P	4 (follow-up: 12 wk)

Abbreviations: BMI, body mass index; C, control; HT, hypertension; I, intervention; NR, not reported; P, parallel; T1DM, type 1 diabetes mellitus; T2DM, type 2 diabetes mellitus; X, crossover.

a Duration reported in weeks (using 4 wk/mo) for duration of <12 months and reported as months/years for duration of 12 months and more.

b A subgroup of participants with eGFR < 60 mL/min/1.73 m^2^.

c Study included other intervention group/s, which was not relevant to this review, therefore this group/s was not included in this analysis.

d Characteristics reported for randomly assigned participants.

**Table 5:** Characteristics of the Included Studies Assessing the Effect of Multiple Lifestyle Interventions on CKD Progression

Study, country	Sample Size (for Analysis)	Age (y)	BMI (kg/m^2^)	CKD Stage	Comorbid Conditions	Design	Study Duration (wk)^a^
Beetham (2022), Australia^82^	160	C: 60.4 ± 10.2; I: 59.5 ± 9.9	C: 33.8 ± 6.8; I: 33.1 ± 6.0	3-4	Diabetes (45%), hyperlipidemia (68%), myocardial infarction (15%), heart failure (4%), peripheral vascular disease (19%), and HT (95%)	P	3 y
Flesher (2011), Canada^83^	40	C: 63.4 ± 11.8; I: 63.4 ± 12.1	NR	2-4 (eGFR 20-60 mL/min/1.73 m^2^)	HT	P	12 mo
Fogelfeld (2017), United States^84^	120	C: 58.69 ± 7.46; I: 56.27 ± 7.46	C: 33.86 ± 7.27 (males) 35.27 ± 8.31 (females); I 32.71 ± 6.12 (males) and 35.69 ± 8.72 (females)	3-4	T2DM	P	24 mo
Headley (2012), United States^85^	21	C: 52.5 ± 10.6 I: 57.5 ± 11.5	C: 34.2 ± 5.7; I: 32.7 ± 7.2	2-4	Mixed	P	48
Hotu (2010), New Zealand^86^	58	C: 60 ± 7.1; I: 63 ± 6.6	C: 35.3 ± 5.8; I: 35.8 ± 6.9	3-4	T2DM and HT	P	12 mo
Ikizler (2018), United States^87^	92	60 ± 11	C: 35.5 (30.6-41.5); I1: 31.0 (28.0-36.2); I2: (diet only) 32.8 (28.7-37.1); I3: 32.8 (30.4-35.8)^b^	3-4	Diabetes (25%) and HT (91%)	P	16
Johns (2020), United States^88^	44	C: 60 ± 10; I: 63 ± 11^c^	BMI ≥ 30: C: 69%; I: 67%^c^	3-5	HT (100%), Diabetes (52%), coronary artery disease (28%), congestive heart failure (18%), peripheral vascular disease (26%), and cerebrovascular disease (26%)	P	24
Kankarn (2019b), Thailand^89^	192	C: 69.69 ± 8.05; I: 69.71 ± 8.81	C: 25.48 ± 4.07; I: 25.19 ± 3.77	2-4	Diabetes (10.9%), HT (26.6%), and Diabetes with HT (41.7%)	P	12 mo
Li (2020), Taiwan^90^	49	51.22 ± 10.98	27.28 ± 4.29	1-4	Diabetes (35%), HT (47%), and dyslipidemia (65%)	P	12.8
Montoya (2016), United States^91^	26	68.1 ± 10.1	NR	4	Diabetes (58.1%), HT (90.2%), and coronary artery disease (51.6%)	P	36
Yamagata (2016), Japan^92^	2136	C: 63.17 ± 8.55; I: 62.79 ± 8.25	C: 25.85 ± 3.85; I: 25.58 ± 3.95	1-5	T2DM (61.3%), HT (90.92%	P (cluster)	3.5 y

Abbreviations: BMI, body mass index; C, control; HT: hypertension; I: intervention; NR: not reported; P: parallel; T1DM: type 1 diabetes mellitus; T2DM: type 2 diabetes mellitus.

a Duration reported in weeks (using 4 wk/mo) for a duration of <12 months and reported as months/years for a duration of 12 months and more.

b Median (interquartile range).

c Characteristics reported for randomly assigned participants.

### Risk-of-Bias Assessment

**Figure 2:**
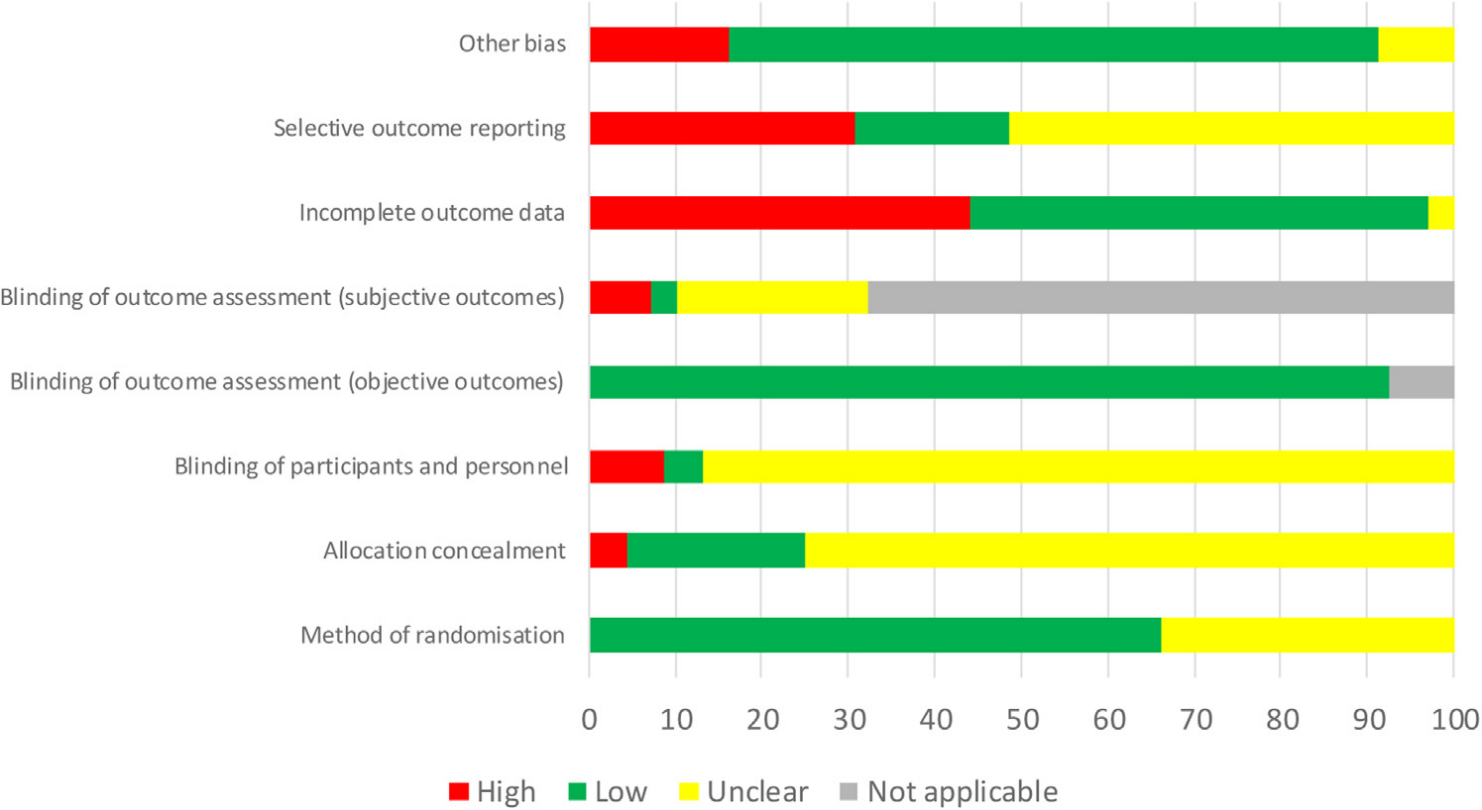
Risk of bias as a proportion of total studies.

The risk-of-bias assessments for included studies are summarized in Figure 2 and outlined in further detail, including the justification for risk-of-bias assessment for each study, in Items S6 and S7.

### Effect of Lifestyle Interventions on Study Outcomes

#### Glomerular Filtration Rate

The glomerular filtration rate was measured or estimated in the included studies using a range of methods, including using the Modification of Diet in Renal Disease Study equation,^35^^,^^40^^,^^55^^,^^67^^,^^78^^,^^80^^,^^82^^,^^85^ Chronic Kidney Disease Epidemiology Collaboration equation,^15^^,^^16^^,^^18^, ^19^, ^20^, ^21^, ^22^, ^23^^,^^27^^,^^46^^,^^54^^,^^64^^,^^66^ Cockroft and Gault formula,^62^ creatinine clearance,^25^^,^^31^^,^^34^^,^^52^^,^^53^^,^^56^^,^^63^^,^^64^^,^^68^^,^^71^ and clearance of ^125^I-iothalamate^24^^,^^60^ or cystatin.^78^

A total of 51 analyses across 47 studies were included in the meta-analysis. A nonsignificant change in the estimated glomerular filtration rate (eGFR) was found (weighted mean difference [WMD], 0.9 mL/min/1.73^2^; 95% CI, −0.6 to 2.3), (Table 6 and Item S9). When studies were separated according to the intervention type, larger changes were found for studies involving exercise interventions (WMD, 1.6 mL/min/1.73^2^; 95% CI, −0.2 to 3.3) (Table 7).

**Table 6:** Changes in Outcomes After Lifestyle Intervention, Compared With Those of Control

Outcome	No. of Studies	No. of Effect Sizes	No. of Participants	Weighted Mean Difference (95% CI), *P* Value	Inconsistency (*I*^2^) (%)
GFR (mL/min/1.73^2^)	47	51	2,852	0.9 (−0.6 to 2.3), 0.251	90.2
ACR (mg/g)	6	7	389	−87 (−212 to 37),0.170	18.1
24-h albuminuria (mg/24 h)	3	3	346	−53 (−56 to −50), <0.001	0
Creatinine (mg/dL)	31	34	2,130	−0.43 (−0.74 to −0.11), 0.008	96.3
Systolic blood pressure (mm Hg)	40	46	2,849	−4.5 (−6.7 to −2.4), <0.001	82.7
Diastolic blood pressure (mm Hg)	37	42	2,614	−2.2 (−3.7 to −0.8), 0.003	76.8
Body weight (kg)	32	38	2,661	−1.1 (−2.0 to −0.1), 0.025	50.1
HbA_1c_ (%)	20	22	1,447	−0.03 (−0.19 to 0.13), 0.717	60.5

Abbreviations: ACR, albumin-creatinine ratio; GFR, glomerular filtration rate; HbA_1c_, hemoglobin A_1c_.

**Table 7:** Changes in Outcomes After Lifestyle Intervention (Categorized by Intervention Type), Compared With Those of Control

Outcome	Intervention Type	No. of Effect Sizes	Weighted Mean Difference (95% CI)	Inconsistency (*I*^2^) (%)
GFR (mL/min/1.73^2^)	Exercise	20	1.6 (−0.2 to 3.3)	62.7
	Diet	19	0.5 (−2.3 to 3.2)	93.8
	Behavioral	4	0.2 (−2.2 to 2.6)	0.0
	Hydration	1	1.0 (−2.5 to 4.5)	—
	Multiple	7	−0.3 (−5.3 to 4.8)	56.4
ACR (mg/g)	Exercise	3	−13 (−259 to 232)	0.0
	Diet	1	−40 (−130 to 49)	—
	Behavioral	2	−304 (−628 to 20)	32.1
	Multiple	1	−9 (−557 to 540)	—
24-h albuminuria (mg/24 h)	Exercise	1	263 (−674 to 1199)	—
	Diet	2	−53 (−56 to −50)	0.0
Creatinine (mg/dL)	Exercise	11	−1.63 (−3.03 to −0.23)	98.8
	Diet	15	0.003 (−0.13 to 0.13)	57.6
	Behavioral	3	−0.08 (−0.30 to 0.15)	0.0
	Multiple	5	−0.04 (−0.30 to 0.23)	39.3
Systolic blood pressure (mm Hg)	Exercise	17	−4.0 (−8.5 to 0.5)	79.6
	Diet	14	−5.2 (−7.6 to −2.9)	61.8
	Behavioral	7	−4.0 (−7.4 to −0.6)	28.2
	Multiple	8	−3.5 (−7.4 to 0.4)	53.4
Diastolic blood pressure (mm Hg)	Exercise	16	−2.4 (−6.1 to 1.3)	86.2
	Diet	13	−1.9 (−3.1 to −0.7)	6.7
	Behavioral	5	−2.3 (−4.3 to −0.3)	1.1
	Multiple	8	−0.70 (−1.7 to 0.3)	0.0
Body weight (kg)	Exercise	13	−0.2 (−3.2 to 2.9)	58.5
	Diet	18	−1.2 (−2.3 to −0.2)	40.6
	Behavioral	2	−1.0 (−3.4 to 1.4)	26.5
	Multiple	5	−4.9 (−9.0 to −0.8)	0.0
HbA_1c_ (%)	Exercise	9	0.01 (−0.38 to 0.40)	74.3
	Diet	4	0.02 (−0.46 to 0.50)	79.2
	Behavioral	4	0.02 (−0.21 to 0.26)	0.0
	Multiple	5	−0.13 (−0.26 to −0.00)	0.0

Abbreviations: ACR, albumin-creatinine ratio; GFR, glomerular filtration rate; HbA_1c_, hemoglobin A_1c_.

Three studies outlined further did not provide information in adequate detail to be included in the primary meta-analysis. Tangri et al^60^ reported a nonsignificant difference in eGFR between the intervention (low protein) and control (usual protein) diets (WMD, −0.3 mL/min/1.73^2^; 95% CI, −2.1 to 1.6). The sensitivity analyses investigating the effect of this study was included in the meta-analysis and found similar results to the primary meta-analysis (effect size: 0.9; 95% CI, −0.4 to 2.3).The 2 cluster randomized controlled trials by Kankarn et al^57^^,^^89^ did not provide the adequate information required to synthesize these studies with trials randomized at the individual level. Both studies found significant improvements in the eGFR after a dietary^57^ and multiple-component intervention,^89^ when compared with those of the control.

#### Albuminuria

Albuminuria was reported as albumin-creatinine ratio (ACR)^34^^,^^35^^,^^42^^,^^69^^,^^79^^,^^84^ and 24-hour albuminuria.^43^^,^^44^^,^^55^^,^^69^ The effect of the lifestyle interventions on ACR was explored using a meta-analysis incorporating 7 effect sizes from 6 studies, with a nonsignificant change (Table 6). When the studies were separated according to the intervention type, larger changes were found for studies involving behavioral interventions (Table 7).

A significant reduction in 24-hour albuminuria was found when results for 3 studies were pooled (WMD, −53 mg/24 h; 95% CI, −56 to −50). When studies were separated according to the intervention type, reductions were found for studies involving only dietary interventions, with a study examining an exercise intervention^43^^,^^44^ reporting a nonsignificant increase in 24-hour albuminuria. However, it should be noted that these pooled effects were driven by the results of 1 study,^55^ which was given a 99.95% weighting in the meta-analysis.

#### Creatinine

A total of 31 studies providing 34 effect sizes were included in the meta-analysis. Lifestyle interventions resulted in a significant reduction in the blood levels of creatinine (WMD, −0.43 mg/dL; 95% CI, −0.74 to −0.11). Larger effects were observed for studies incorporating exercise interventions (Table 7). In addition, Tangri et al^60^ was not able to be included in the primary meta-analysis because of the reporting mean difference between the intervention and control only. Tangri et al^60^ found a significant reduction in the blood levels of creatinine in the intervention diet (low protein), compared with those of the control (usual protein) (WMD, −0.22 mg/d; −0.36 to −0.08). The sensitivity analyses investigating the effect of this study was included in the meta-analysis and found similar results to the primary meta-analysis (effect size, −0.48; 95% CI, −0.78 to −0.18; *P* = 0.002).

#### Systolic and Diastolic Blood Pressure Levels

A total of 40 studies reporting 46 effect sizes and 37 studies reporting 42 effect sizes were included in the meta-analyses for systolic and diastolic blood pressure levels, respectively. Significant reductions in both systolic and diastolic blood pressure levels were found after lifestyle intervention (systolic blood pressure: WMD, −4.5 mm Hg; 95% CI, −6.7 to −2.4; diastolic blood pressure: WMD, −2.2 mm Hg; 95% CI, −3.7 to −0.8) (Table 6). When studies were separated according to the intervention type, similar results were found among the subgroups (Table 7). Two cluster randomized trials did not provide adequate information for inclusion in the meta-analyses for both systolic and diastolic blood pressure levels.^57^^,^^89^ After dietary and multiple interventions, significantly lower systolic and diastolic blood pressure was found in 1 study,^57^ with no significant changes in the second study,^89^ respectively.

#### Body Weight

A total of 38 effect sizes reported in 32 studies were included in the meta-analysis. Lifestyle intervention was found to result in significant reductions in body weight, when compared with those of the control (WMD, −1.1 kg; 95% CI, −2.0 to −0.1) (Table 6). Larger reductions in weight were found after interventions incorporating multiple intervention components (eg, dietary and exercise interventions) (Table 7).

#### Blood Glucose Control

Twenty studies reporting 22 effect sizes were included in the meta-analysis assessing the effect of lifestyle interventions on hemoglobin A_1c_ (HbA_1c_), with nonsignificant changes found (Table 6). Although the magnitude of the effect on HbA_1c_ was overall similar among the subgroups when the studies were separated according to the intervention type, decreases in HbA_1c_ levels were found only for studies assessing multiple interventions (Table 7).

### Sensitivity Analyses

When the sensitivity analyses were conducted using correlation coefficients of 0.25, 0.5, and 0.75 for crossover studies, similar results to primary analyses were found, regardless of the correlation coefficient used (Item S10). In addition, findings were similar for most outcomes for sensitivity analyses exploring the effect of different analysis scenarios, including sensitivity analyses excluding studies with imputed standard deviations, excluding a cluster randomized trial,^92^ and pooling the multiple intervention groups of Ikizler et al^87^ (Item S11). The exceptions to this were creatinine levels and body weight, wherein the results became nonsignificant when studies with imputed standard deviations were excluded. In addition, for most outcomes, leave-1-out sensitivity analyses found similar results if each study was omitted, suggesting no 1 individual study unduly influenced the results (Item S12). However, when an individual study was excluded for creatinine levels,^24^ the pooled effect changed to become nonsignificant and significant, respectively.

### Quality of Life

The effect of lifestyle interventions on QoL were investigated in 20 studies. Quality of life was assessed using a range of tools, including the 36-item short form survey,^16^^,^^17^^,^^28^, ^29^, ^30^^,^^35^^,^^36^^,^^43^^,^^44^^,^^47^^,^^75^^,^^88^ Kidney Disease Quality of Life Short Form questionnaire,^38^^,^^43^, ^44^, ^45^^,^^47^^,^^49^^,^^90^ 12-item short form survey,^41^^,^^54^^,^^79^ World Health Organization Quality of Life-BREF,^74^^,^^78^ Assessment of Quality of Life questionnaire,^58^ EuroQoL 5-dimensional,^36^ Kidney Disease Quality of Life 36-item survey,^41^ RAND 36-Item Short Form Health Survey,^39^ and Veterans RAND-12^42^ (Item S14).

Significant improvements in QoL after lifestyle interventions, compared with those of the control, were reported in 31 domains reported among 11 studies.^16^^,^^17^^,^^28^, ^29^, ^30^^,^^38^^,^^39^^,^^41^^,^^43^, ^44^, ^45^^,^^47^^,^^74^^,^^75^^,^^90^ This included improvements to specific domains, such as cognitive function,^45^^,^^47^ physical function,^28^, ^29^, ^30^^,^^38^^,^^39^^,^^41^^,^^47^^,^^75^^,^^90^ vitality,^47^ pain,^28^, ^29^, ^30^^,^^39^ mental function,^38^^,^^41^^,^^75^ fatigue,^39^^,^^45^ sleep,^45^ quality of social interaction,^43^^,^^44^ and work status^43^^,^^44^ (Item S14). Nonsignificant improvements were reported in 90 domains across 14 studies.^28^, ^29^, ^30^^,^^35^^,^^36^^,^^39^^,^^42^^,^^44^^,^^45^^,^^47^^,^^54^^,^^58^^,^^78^^,^^79^^,^^88^^,^^90^ No difference in QoL between intervention and control arms were reported for 3 domains across 3 studies,^47^^,^^49^^,^^58^ whereas nonsignificant reductions in QoL after lifestyle interventions, compared with those of control, were reported in 17 domains across 10 studies.^36^^,^^43^, ^44^, ^45^^,^^47^^,^^49^^,^^54^^,^^58^^,^^78^^,^^79^^,^^88^ No studies reported statistically significant reductions in QoL after lifestyle interventions, compared with those of the control.

### Small Study Effects

Contour funnel plots were generated for outcomes with 10 or more effect sizes (eGFR, creatinine, systolic blood pressure, diastolic blood pressure, body weight, and HbA_1c_), with funnel plots and the results of Egger test presented in Item S13. Funnel plot asymmetry was detected for body weight (bias, −0.659; 95% CI, −1.138 to 0.180; *P* = 0.008), indicating the presence of small study effects that may have been due to publication bias. Use of the trim-and-fill method did result in a significant effect of lifestyle intervention on body weight (WMD, 0.3; 95% CI, 0.1-0.9; *P* = 0.025) ) (Item S13), suggesting that estimated unpublished studies may have modified the effect. Funnel plot asymmetry was not detected for all other outcomes.

### The Certainty of the Body of Evidence

The certainty of the body of evidence was determined using GRADE^14^ (Item S15). The certainty of the body of evidence was very low for eGFR, creatinine, systolic blood pressure, and diastolic blood pressure, after being downgraded owing to the risk of bias and inconsistency; very low for ACR owing to the risk of bias and imprecision; very low for body weight owing to the risk of bias, inconsistency, and the likelihood of publication bias; low for HbA_1c_ owing to the risk of bias and inconsistency; moderate for 24-hour albuminuria owing to imprecision; and moderate for QoL owing to the risk of bias. Consideration of the studies that were not able to be included in the calculation of the pooled effects did not change these assessments.^53^^,^^57^^,^^89^

## Discussion

This systematic review on the effects of lifestyle interventions on the risk factors for and progression of kidney disease and the QoL in people with CKD found that lifestyle interventions resulted in significant improvement in systolic and diastolic blood pressure levels and in body weight. Statistically significant improvements in creatinine levels and 24-hour albuminuria were also found but should be interpreted with caution because of the large influence of a single study for each outcome (Castaneda et al^24^ and Hwang et al^55^ respectively). In addition, in the case of creatinine, these changes were small and not clinically significant, which may explain why these results did not correspond to significant changes to the eGFR. The narrative synthesis indicated that lifestyle intervention resulted in improvements in the QoL of patients with CKD. This included domains important to patients, such as fatigue, sleep, and pain. The certainty of evidence was very low for most outcomes, largely owing to the risk of bias and inconsistency of the study results.

Identifying successful lifestyle interventions in CKD can guide future clinical practice. When studies were separated according to the type of intervention, findings varied among the outcomes. Although results should be interpreted with caution owing to the variation in the number of studies within each subgroup, exercise interventions seemed to result in the greatest improvements in eGFR and creatinine, whereas dietary interventions resulted in large improvements in albuminuria and systolic blood pressure. The underlying mechanism of the reduction in 24-hour albuminuria by diet is unknown and challenging to tease out, given people consume whole food dietary patterns and not nutrients, such as protein or sodium in isolation. In addition, variations in the components of lifestyle interventions make synthesis of evidence challenging. Given these challenges, the optimal intervention remains to be determined. However, it is important to note that lifestyle interventions (particularly diet) are equally as effective as pharmaceutical strategies for reducing systolic blood pressure and may have positive additive effects on 24-hour albumin excretion in those prescribed sodium-glucose cotransporter 2 inhibitors.^93^

Although a previous systematic review explored lifestyle interventions in CKD, the focus was predominantly on evaluating the behavior change techniques used and did not pool results using a meta-analysis.^3^ Evangelidis et al^3^ examined 26 lifestyle interventions: 11 diet, 8 physical activity, and 7 general lifestyle advice. The authors concluded that the most promising interventions included education with other behavior change techniques, such as persuasion, modelling, and incentivization. Our findings contrast with this review, whereby we found that multimodal interventions did not always produce the greatest effects, except for the outcome of HbA_1c_ and body weight. These differences may be partly because of our review incorporating a meta-analysis, which allowed us to quantify the effects. In addition, Evangelidis et al^3^ focused on interventions with a behavioral component alone, and reported only the primary outcomes for each study. In comparison, our review included a larger number of studies and evaluated all eligible outcomes reported in those studies, which may explain the variations in our findings.

Overall, our results did not seem to be changed in the sensitivity analyses, suggesting the findings were largely robust across varying data inclusion and analysis scenarios. However, some exceptions were found, which warrant further discussion. In the case of creatinine, excluding studies with imputed standard deviations resulted in a pooled effect that was no longer significant. This is likely to be because of the removal of the study by Castaneda et al,^24^ as indicated by the results of the leave-1-out analysis. Although Castaneda et al^24^ reported a substantially lower final creatinine level in intervention participants than those undertaking the control arm, these intervention participants started with a lower creatinine value, which may have influenced these results. In addition, exclusion of studies with imputed standard deviation from the body weight meta-analysis resulted in the effect becoming no longer statistically significant, although the magnitude of the effect was similar overall (WMD of −1.076 kg in the primary analysis vs −0.886 kg in the sensitivity analysis).

The variability in the outcomes and measures limited our ability to comprehensively evaluate the effect on QoL. This is an ongoing challenge in nephrology trials, and the production of a core outcome set in trials of people with CKD will improve the relevance, transparency, and effect of future research.^94^ The outcomes reported in the dietary trials are not included in previous standardized outcomes in the nephrology outcome sets but are of particular importance when discussing lifestyle trials.^95^ The 2020 KDOQI Clinical Practice Guidelines for Nutrition^96^ now recommend that patients adopt dietary patterns, such as a Mediterranean style approach to eating. The implications of this new approach to nutrient prescription should include a move toward reporting diet quality measures in lifestyle trials instead of nutrient-related outcomes. In this review, there were only 4 trials that tested manipulation of dietary patterns as a lifestyle modification, and most were less than 12 weeks duration. These shorter study designs are also inconsistent with the definitions of lifestyle modification,^97^ whereby alterations are made to behavior for months or years. Similar calls for consistency in reporting for exercise trials have also been published,^98^ to increase the rigor of comparisons.

The strengths of this review were the robust method, including duplicate screening, review, and data extraction, and a range of sensitivity analyses were conducted. The limitations include restricting to published studies only, and studies published in the English language, meaning some potentially eligible studies may have been missed. The definition of lifestyle intervention and categorization of intervention type was based on subjective judgment by researchers. In addition, there was a substantial variation among the types of interventions, which comprised lifestyle interventions. This variation has been considered by examining the effects of different intervention types using subgroup analyses. Many of the included studies were not powered to detect changes in the outcomes of interest for this review. Although this issue is somewhat alleviated by pooling using meta-analysis, this should be considered when interpreting results. Treatment of CKD is expensive from a societal and personal perspective.^99^ A need exists for future studies to conduct health economic evaluations of lifestyle interventions and to systematically compare the cost effectiveness of these interventions.

To conclude, this systematic review found that lifestyle interventions may affect some risk factors for progression of CKD, such as blood pressure, albuminuria, and weight. However, the quality of the evidence base is very low, and further synthesis, such as outcomes relating to QoL are limited by variations in the measurements used. Future studies with more robust designs are needed that are also guided by outcomes important to patients and are of longer duration.

## Supplementary Material

Supplementary File (PDF) Item S1-S15.
