# Supplementary material for: Lifestyle Interventions, Kidney Disease Progression, and Quality of Life: A Systematic Review and Meta-analysis
Source: Kidney Med. 2023 Apr 18;5(6):100643. doi: 10.1016/j.xkme.2023.100643 (PMC10205767; doi:10.1016/j.xkme.2023.100643)
Supplement: Supplementary File (PDF) — Item S1-S15. [file mmc1.pdf]

Item S1: PRISMA checklist

Section/topic	#	Checklist item	Reported on page #
**TITLE**			
Title	1	Identify the report as a systematic review, meta-analysis, or both.	1
**ABSTRACT**			
Structured summary	2	Provide a structured summary including, as applicable: background; objectives; data sources; study eligibility criteria, participants, and interventions; study appraisal and synthesis methods; results; limitations; conclusions and implications of key findings; systematic review registration number.	2-3
**INTRODUCTION**			
Rationale	3	Describe the rationale for the review in the context of what is already known.	5
Objectives	4	Provide an explicit statement of questions being addressed with reference to participants, interventions, comparisons, outcomes, and study design (PICOS).	5
**METHODS**			
Protocol and registration	5	Indicate if a review protocol exists, if and where it can be accessed (e.g., Web address), and, if available, provide registration information including registration number.	5
Eligibility criteria	6	Specify study characteristics (e.g., PICOS, length of follow-up) and report characteristics (e.g., years considered, language, publication status) used as criteria for eligibility, giving rationale.	Table 1
Information sources	7	Describe all information sources (e.g., databases with dates of coverage, contact with study authors to identify additional studies) in the search and date last searched.	6
Search	8	Present full electronic search strategy for at least one database, including any limits used, such that it could be repeated.	S1
Study selection	9	State the process for selecting studies (i.e., screening, eligibility, included in systematic review, and, if applicable, included in the meta-analysis).	7, S3
Data collection process	10	Describe method of data extraction from reports (e.g., piloted forms, independently, in duplicate) and any processes for obtaining and confirming data from investigators.	7, S3
Data items	11	List and define all variables for which data were sought (e.g., PICOS, funding sources) and any assumptions and simplifications made.	7

Risk of bias in individual studies	12	Describe methods used for assessing risk of bias of individual studies (including specification of whether this was done at the study or outcome level), and how this information is to be used in any data synthesis.	7
Summary measures	13	State the principal summary measures (e.g., risk ratio, difference in means).	7 -8
Synthesis of results	14	Describe the methods of handling data and combining results of studies, if done, including measures of consistency (e.g., I^2) for each meta-analysis.	7-8, S4

**Section/topic**	**#**	**Checklist item**	**Reported on page #**
Risk of bias across studies	15	Specify any assessment of risk of bias that may affect the cumulative evidence (e.g., publication bias, selective reporting within studies).	8
Additional analyses	16	Describe methods of additional analyses (e.g., sensitivity or subgroup analyses, meta-regression), if done, indicating which were pre-specified.	S4
**RESULTS**			
Study selection	17	Give numbers of studies screened, assessed for eligibility, and included in the review, with reasons for exclusions at each stage, ideally with a flow diagram.	Figure 1, 9
Study characteristics	18	For each study, present characteristics for which data were extracted (e.g., study size, PICOS, follow-up period) and provide the citations.	Table 2, S5
Risk of bias within studies	19	Present data on risk of bias of each study and, if available, any outcome level assessment (see item 12).	Figure 2, S6, S7
Results of individual studies	20	For all outcomes considered (benefits or harms), present, for each study: (a) simple summary data for each intervention group (b) effect estimates and confidence intervals, ideally with a forest plot.	S8, S9
Synthesis of results	21	Present results of each meta-analysis done, including confidence intervals and measures of consistency.	S9, Table 3, Table 4
Risk of bias across studies	22	Present results of any assessment of risk of bias across studies (see Item 15).	14, 15, S13, S15
Additional analysis	23	Give results of additional analyses, if done (e.g., sensitivity or subgroup analyses, meta-regression [see Item 16]).	13, S10, S11, S12
**DISCUSSION**			

Summary of evidence	24	Summarize the main findings including the strength of evidence for each main outcome; consider their relevance to key groups (e.g., healthcare providers, users, and policy makers).	15 - 18
Limitations	25	Discuss limitations at study and outcome level (e.g., risk of bias), and at review-level (e.g., incomplete retrieval of identified research, reporting bias).	17 - 18
Conclusions	26	Provide a general interpretation of the results in the context of other evidence, and implications for future research.	18
**FUNDING**			
Funding	27	Describe sources of funding for the systematic review and other support (e.g., supply of data); role of funders for the systematic review.	2

From: Moher D, Liberati A, Tetzlaff J, Altman DG, The PRISMA Group (2009). Preferred Reporting Items for Systematic Reviews and Meta-Analyses: The PRISMA Statement. PLoS Med 6(7): e1000097. doi:10.1371/journal.pmed1000097

Item S2: Search strategies for all databases

Pubmed

((((kidney failure, chronic[MeSH Terms] OR Renal Insufficiency, Chronic[MeSH Terms] OR CRF OR CKD OR "end stage kidney failure" OR "end-stage kidney failure" OR "end stage kidney disease" OR "end-stage kidney disease" OR "chronic renal failure" OR "end-stage renal disease" OR "end stage renal disease" OR "end-stage renal failure" OR "end stage renal failure" OR "chronic renal disease" OR "chronic kidney disease")) AND ((quality of life[MeSH Terms] OR body weight[MeSH Terms] OR urine[MeSH Terms] OR Hemoglobin A, Glycosylated[MeSH Terms] OR blood pressure[MeSH Terms] OR Creatinine[MeSH Terms] OR Albuminuria[MeSH Terms] OR glomerular filtration rate[MeSH Terms] OR QoL OR "quality of life" OR "glycosylated hemoglobin" OR "glycosylated haemoglobin" OR "glycated hemoglobin" OR "glycated haemoglobin" OR HbA1c OR "urine volume" OR "body weight" OR "blood pressure" OR creatinine OR albuminuria OR eGFR OR "glomerular filtration rate"))) AND (((lifestyle[MeSH Terms] OR exercise[MeSH Terms] OR diet[MeSH Terms] OR dietary protein[MeSH Terms] OR sodium, dietary[MESH Terms] OR potassium[MESH Terms] OR electrolytes[MESH Terms] OR protein OR energy OR sodium OR salt OR potassium OR electrolyte OR phosph* OR hydrat* OR fluid* OR behavior* OR behaviour* OR psycholog* OR "progressive resistance training" OR "physical activity" OR exercise OR nutrition* OR diet* OR lifestyle)))) AND ((randomized controlled trial[pt] OR controlled clinical trial[pt] OR randomized[tiab] OR placebo[tiab] OR drug therapy[sh] OR randomly[tiab] OR trial[tiab] OR groups[tiab] NOT (animals[mh] NOT humans[mh])))

Medline (via EBSCO)

(((((MH "kidney failure, chronic+”) OR (MH "Renal Insufficiency, Chronic+”) OR CRF OR CKD OR "end stage kidney failure" OR "end-stage kidney failure" OR "end stage kidney disease" OR "end-stage kidney disease" OR "chronic renal failure" OR "end-stage renal disease" OR "end stage renal disease" OR "end-stage renal failure" OR "end stage renal failure" OR "chronic renal disease" OR "chronic kidney disease")) AND (((MH "quality of life+”) OR (MH "body weight+”) OR (MH “urine+”) OR (MH "Hemoglobin A, Glycosylated+”) OR (MH "blood pressure+”) OR (MH “Creatinine+”) OR (MH “Albuminuria+”) OR (MH "glomerular filtration rate+”) OR QoL OR "quality of life" OR "glycosylated hemoglobin" OR "glycosylated haemoglobin" OR "glycated hemoglobin" OR "glycated haemoglobin" OR HbA1c OR "urine volume" OR "body weight" OR "blood pressure" OR creatinine OR albuminuria OR eGFR OR "glomerular filtration rate"))) AND ((((MH "Life Style+") OR (MH “exercise+”) OR (MH “diet+”) OR (MH "Dietary Proteins+") OR (MH "sodium, dietary+”) OR (MH “potassium+”) OR (MH “electrolytes+”) OR protein OR energy OR sodium OR salt OR potassium OR electrolyte OR phosph* OR hydrat* OR fluid* OR behavior* OR behaviour* OR psycholog* OR "progressive resistance training" OR "physical activity" OR exercise OR nutrition* OR diet* OR lifestyle)))) AND ((PT "randomized controlled trial" OR PT "controlled clinical trial" OR (TI randomized OR AB randomized) OR (TI placebo OR AB placebo) OR "Drug Therapy" OR

(TI randomly OR AB randomly) OR (TI trial OR AB trial) OR (TI groups OR AB groups) NOT ((MH "animals+") NOT (MH "humans+"))))

CINAHL (via EBSCO)

(((((MH "kidney failure, chronic"+) OR (MH "Renal Insufficiency, Chronic"+) OR CRF OR CKD OR "end stage kidney failure" OR "end-stage kidney failure" OR "end stage kidney disease" OR "end-stage kidney disease" OR "chronic renal failure" OR "end-stage renal disease" OR "end stage renal disease" OR "end-stage renal failure" OR "end stage renal failure" OR "chronic renal disease" OR "chronic kidney disease")) AND (((MH "quality of life"+) OR (MH "body weight"+) OR (MH urine+) OR (MH "Hemoglobin A, Glycosylated"+) OR (MH "blood pressure"+) OR (MH Creatinine+) OR (MH Albuminuria+) OR (MH "glomerular filtration rate"+) OR QoL OR "quality of life" OR "glycosylated hemoglobin" OR "glycosylated haemoglobin" OR "glycated hemoglobin" OR "glycated haemoglobin" OR HbA1c OR "urine volume" OR "body weight" OR "blood pressure" OR creatinine OR albuminuria OR eGFR OR "glomerular filtration rate"))) AND ((((MH "Life Style+") OR (MH exercise+) OR (MH diet+) OR (MH "Dietary Proteins+") OR (MH "sodium, dietary"+) OR (MH potassium+) OR (MH electrolytes+) OR protein OR energy OR sodium OR salt OR potassium OR electrolyte OR phosph* OR hydrat* OR fluid* OR behavior* OR behaviour* OR psycholog* OR "progressive resistance training" OR "physical activity" OR exercise OR nutrition* OR diet* OR lifestyle)))) AND ((PT "randomized controlled trial" OR PT "controlled clinical trial" OR (TI randomized OR AB randomized) OR (TI placebo OR AB placebo) OR "Drug Therapy" OR (TI randomly OR AB randomly) OR (TI trial OR AB trial) OR (TI groups OR AB groups) NOT ((MH animals+) NOT (MH human+))))

Cochrane library

#1 MeSH descriptor: [Kidney Failure, Chronic] explode all trees

#2 MeSH descriptor: [Renal Insufficiency, Chronic] explode all trees

#3 CRF or CKD or "end stage kidney failure" or "end-stage kidney failure" or "end stage kidney disease" or "end-stage kidney disease" or "chronic renal failure" or "end-stage renal disease" or "end stage renal disease" or "end-stage renal failure" or "end stage renal failure" or "chronic renal disease" or "chronic kidney disease"

#4 #1 or #2 or #3

#5 QoL or "quality of life" or "glycosylated hemoglobin" or "glycosylated haemoglobin" or "glycated hemoglobin" or "glycated haemoglobin" or HbA1c or "urine volume" or "body weight" or "blood pressure" or creatinine or albuminuria or eGFR or "glomerular filtration rate"

#6 MeSH descriptor: [Quality of Life] explode all trees

#7 MeSH descriptor: [Body Weight] explode all trees

#8 MeSH descriptor: [Urine] explode all trees

#9 MeSH descriptor: [Glycated Hemoglobin A] explode all trees

#10 MeSH descriptor: [Blood Pressure] explode all trees

#11 MeSH descriptor: [Creatinine] explode all trees

#12 MeSH descriptor: [Albuminuria] explode all trees

#13 MeSH descriptor: [Glomerular Filtration Rate] explode all trees

#14 #5 or #6 or #7 or #8 or #9 or #10 or #11 or #12 or #13

#15 protein or energy or sodium or salt or potassium or phosph* or electrolyte* or hydrat* or fluid* or behavior* or behaviour* or psycholog* or "progressive resistance training" or "physical activity" or exercise or nutrition* or diet* or lifestyle

#16 MeSH descriptor: [Life Style] explode all trees

#17 MeSH descriptor: [Exercise] explode all trees

#18 MeSH descriptor: [Diet] explode all trees

#19 MeSH descriptor: [Dietary Proteins] explode all trees

#20 MeSH descriptor: [Sodium, Dietary] explode all trees

#21 MeSH descriptor: [Potassium] explode all trees

#22 MeSH descriptor: [Electrolytes] explode all trees

#23 #15 or #16 or #17 or #18 or #19 or #20 or #21 or #22

#24 #4 and #14 and #23

Item S3: Additional details of data extraction methods

Where required details were missing from the published reports, authors were contacted via email to request the information. Where data was presented in figures, data was extracted using Web Plot Digitizer [1]. Where available, mean change and standard deviation, standard error, or 95% confidence intervals (95% CI) were extracted, and where this data was not provided, mean final values and respective standard deviation, standard error, or 95% CI were used as recommended by the Cochrane Handbook for Systematic Reviews of Interventions [2]. Standard errors and 95% CI were then converted to standard deviations using the formulas outlined in the Cochrane Handbook [2]. In the case that median and range or interquartile range were reported, these were converted to mean and standard deviation using formulas from Wan et al. [3] and Luo et al. [4]. If measures of variability were not available, standard deviations were imputed by taking the average standard deviations from other included studies. In the case of one study which had a standard deviation of 0 for the intervention group (calculated from range) [5] for HbA1c, this was treated as 0.0001 for the purpose of data synthesis, although the impact of this imputation was assessed via sensitivity analysis (outlined below). Study results were converted to common units, for example for studies which reported creatinine as µmol/L, these were converted to mg/dL by multiplying by 0.0113.

Item S4: Additional details of meta-analysis methods

Crossover studies were initially treated in the same way as parallel studies. While this approach results in a unit-of-analysis issue, it was deemed appropriate due to the absence of concerns regarding carry-over or period effects and given that it is considered a conservative approach to managing crossover studies [2]. To complement this analysis, sensitivity analyses were conducted using paired analysis of cross-over studies with correlation coefficients of 0.25, 0.5 and 0.75, in order to determine if crossover studies were underweighted, as conducted in our previous reviews [6,7].

Where multiple eligible intervention (for example two dietary interventions) or control groups were included in a single study, these were pooled for the meta-analysis [2]. In the case of one study which reported three intervention groups consisting of different types of interventions [8], these groups were included separately in the meta-analysis, with the sample size of the control group split to avoid a unit-of-analysis issue. Sensitivity analyses pooling these three intervention groups into one intervention group were also conducted, to identify whether this approach impacted on results.

In the case of cluster randomised trials, where possible these were included in the meta-analysis by calculating the appropriate sample size using the approach outlined in the Cochrane Handbook [2]. Sensitivity analyses were conducted excluding cluster randomised trials, to determine the impact of including these studies. Where cluster randomised trials did not provide

adequate information required to synthesise these studies with trials randomized at the individual level, cluster randomized trials were explored via the narrative synthesis.

Further sensitivity analyses were conducted excluding studies with imputed standard deviations. In addition, one study reported only differences between study groups (as mean difference and 95% CI) [9]. To ensure data could be pooled as weighted mean differences for affected outcomes, this study was not included in primary meta-analyses, however sensitivity analyses were also conducted including this study to assess if it influenced results. In addition, leave-one-out' sensitivity analyses (using the metaninf command) were also conducted to explore the effect of removing each individual study from each primary meta-analysis.

Item S5: summary table with intervention and control details

Study ref, country	Intervention details†	Control details†
Aoike (2015), Brazil	Home-based moderate intensity aerobic exercise program (3 times/week). Initially 30 min walk/session, then 10 min increment increase every 4 weeks until 8 weeks	Usual care
Aoike (2018)/Gomes (2017), Brazil	1. Home-based moderate aerobic activity (3 times/week). Initially 30 min walk/session, then 10 min increment increase every 4 weeks until 8 weeks<br>2. As 1) but center-based activity under supervision of an exercise physiologist.	Usual care
Barcellos (2018), Brazil	3 x 60 min exercise sessions per week (warm up exercises and joint flexibility, and aerobic and muscular endurance exercises)	Usual care
Baria (2014), Brazil	1. Home-based moderate aerobic activity (3 times/week). Initially 30 min walk/session, then 10 min increment increase every 4 weeks until 12 weeks<br>2. As (1) but center-based activity under supervision of an exercise physiologist.	Usual care
Beetham (2022), Australia	Exercise: 150 mins of moderate intensity exercise/week (8 weeks supervised by clinical exercise physiologist, followed by home-based). Included both aerobic and resistance training. Progression tailored individually<br>Other lifestyle components: 4 weeks group behaviour and lifestyle modification (facilitated by dietitian and psychologist). Program focused on sustainable diet and lifestyle change to aid weight loss, and nutrition component adhered to CKD guidelines	Usual care
Caldiroli (2022), Italy	Low protein diet (0.6 - 0.7g/kg), including provision of commercial protein free products.	Normoprotein diet (0.8g/kg), with plant sources of protein preferred
Campbell (2008), Australia	One individualised counselling session using self-management principles for CKD while following the ADA medical nutrition therapy framework. The session was followed by fortnightly telephone follow-up for one month, then monthly.	Standard care (generic written nutrition information containing an overview of nutrition advice for chronic kidney disease and co-morbidity management)

Castaneda (2001), US	Resistance exercise 3 times/ week (3 sets of eight repetitions on 5 exercise machines)	Participants performed exercises designed to not have a physiological impact
Chilelli (2015), Italy	Low advanced glycation end product diet and cooking methods	Standard diet for CKD
Clark (2013), Canada	Encouraged to consume additional 1-1.5L of water/day on top of current fluid intake based on sex, weight and 24hr urine osmolality.	Usual fluid intake if 24hr urine osmolality >500 mOsm/kg or to decrease by 1 cups/day if urine osmolality between 300-500 mOsm/kg or decrease by 1-2cups/day if <300 mOsm/kg
Correa (2021)/Correa (2021)/Deus (2021)/Deus (2022), Brazil	1. *RT*: resistance training 3 days/week, intensity adjusted every 2 months (0 - 2 months: 1-3 sets of 12 repetitions at 50% 1-repetition maximum; 2 - 4 months: 2-3 sets of 10 repetitions at 60% 1-repetition maximum; 4 - 6 months: 3 sets of 8 repetitions at 70% 1-repetition maximum, 3 days/week)<br>2. *RT+ BFR*: resistance training 3 days/week, intensity adjusted every 2 months (0 - 2 months: 1-3 sets of 12 repetitions at 30% 1-repetition maximum; 2 - 4 months: 2-3 sets of 10 repetitions at 40% 1-repetition maximum; 4 - 6 months: 3 sets of 8 repetitions at 50% 1-repetition maximum). Restriction of 50% of the measured systolic blood pressure applied on each arm.	No exercise program
de Brito-Ashurst (2013), UK	Dietetic advice, two weekly cooking classes delivered in a community kitchen of a traditional Bangladeshi dish including a 50% salt reduced version. Fortnightly telephone calls to reinforce advice and set new targets.	Usual care (low sodium general dietary advice sheet not specifically adapted for Bangladeshi diets).
Eidemak (1997), Denmark	Individually designed exercise program consisting of bicycle ergometer, running, swimming, and walking. Exercise duration and intensity increased over time to approximately 60-70% maximal exercise capacity.	Usual activity

Facchini (2003), US	Reduced carbohydrate intake (50% of usual); red meat replaced with poultry, fish, eggs, dairy, and soy; fluid limited to tea, water and red wine; extra virgin olive oil used for frying and dressings.	An isocaloric diet with protein restricted to 0.8g/kg/IBW
Flesher (2011), Canada	Group CKD nutrition class, CKD cooking class with a dietitian and cook educator (2-hour sessions, over four weeks, plus shopping tour), CKD cookbook, and 12-week exercise program led by exercise physiologist and nurse (3 x 1hour session/week including aerobic, strength, and flexibility components, commenced after 6-month timepoint). All classes included self-management focus.	Standard CKD care
Fogelfeld (2017), US	Group diet instruction, followed by individual visits with all study staff (endocrinologist, nephrologist, nurse practitioner, certified diabetes educator/dietitian, and research coordinator). All study staff included to improve coordination of care and integrate treatment protocols. Visit frequency: monthly for first 6 months, then bimonthly for next 18 months (15 visits over two years).	Usual care (visits with primary care physicians, diabetes, kidney clinics)
Goraya (2014)/Goraya (2019), US	Provided with fruit and vegetables (emphasizing base-producing fruits and vegetables)**	Usual care
Grazioli (2022), Italy	*I1*: 60 mins of home-based activity (including aerobic phase and strength-focused phase) three times per week, plus two 32 g functional bars per day (energy: 122 kcal, potassium: 200 mg per bar)<br>*I2*: 60 mins of home-based activity (including aerobic phase and strength-focused phase) three times per week	*C1*: two 32 g functional bars per day (energy: 122 kcal, potassium: 200 mg per bar)<br>*C2:* control group (no further details given)
Greenwood (2014), UK	Individualised program (aerobic and resistance). Participants worked towards 2 x 20 min sessions 3 times/week, increasing to 1 x 40 min session 3 times/week. Resistance training at 80% of one repetition maximum (starting at 1 -2 sets x 10 reps, increasing to 3 sets x 8 - 10 reps). Pts also met with kidney physiotherapist for 40 min individual session at baseline	Usual care

Hamidianshirazi (2022), Iran	Individualised CKD diet therapy based on NKF KDOQI guidelines supported by nutrition counseling:<br>0.75 g protein/kg/day and 30-35 kcal/kg/day with sodium restriction)	Usual care
Headley (2012), US	3 x supervised exercise sessions per week for 48 weeks (including flexibility and aerobic components). Duration of exercise increased to 45 mins/session. From weeks 24 - 48, allowed to perform resistance exercise 2 x week (in addition to aerobic).<br>Received CKD nutritional counselling from dietitian	Usual care
Headley (2014)/Headley (2017)/Miele (2017),US	3 x supervised exercise sessions per week for 16 weeks (starting at 15 - 30 mins and increasing to 55 mins)	Usual care
Hiraki (2017), Japan	Home-based aerobic (30 mins walking or 8000-10000 steps/day) and resistance training exercises (upper and lower limb resistance exercises 20 - 30 reps per exercise, 3 x week).	Not given any exercise instructions and asked to carry out daily activities as usual
Hotu (2010), NZ	Monthly community visits by a nurse-led, culturally appropriate Māori or Pacific health-care assistant. Compliance with medication use and BP checked. Participants offered transport to collect medication, to lab for tests, and to follow-up clinic appointments.	routine doctor and CKD/diabetes hospital outpatient clinical care.
Hwang (2014), South Korea	Intensive low salt intervention with target of <100mEq/d sodium ($\geq$25% reduction of salt intake) - supported by dietary consultant and telephone feedback for 30 mins/week	Standard low salt diet education at an outpatient clinic and written information on sodium content of commonly consumed food products
Ihle (1989), Australia	Protein restricted diet of 0.4g/kg/day of body weight, using protein foods with high biological value of 75-80%. Energy intake of 35-40kcal/kg/day.	Ad libitum food intake with a protein intake of at least 0.75g/kg/day of body weight.

Ikizler (2018), US	1. Exercise and dietary: moderate physical activity of 30-45mins 3 x weekly customized to participants baseline fitness status and building to 60-80% of VO^2 max<br>2. Dietary: reduced caloric intake of 10-15% from baseline (~300-500kcal/day)<br>3. Exercise: moderate physical activity of 30-45mins 3 x weekly customized to participants baseline fitness status and building to 60-80% of VO2 max	Dietary recommendations to include fresh unprocessed plant intake with moderate levels of lean protein and fats such as omega-3 and monounsaturated fats.
Joboshi (2016), Japan	Steps 1-6 of the Encourage Autonomous Self-Enrichment (EASE) program action plan covering self-management, goal setting, self-monitoring administrated by a trained nurse and monitored with weekly phone calls, monthly interviews and outpatient visits	Standard patient education and care
Johns (2020), US	Group-based care (1 per month) - sessions included self-care activities (measurement of BP and other vitals) and discussion (lead by nephologist or kidney dietitian) of set topic sourced from existing educational materials from the National<br>Kidney Disease Education Program (including kidney function, blood pressure control, medication side effects, avoidance of sodium and phosphorus, physical activity).	Received printed health education materials from the National Kidney Disease Education Program
Kankarn (2019a), Thailand	Individualised nutritional management: included meal planning and personalized care plan, with home visits, plus group meeting once/ month consisting of a 60 min dietitian talk. Nutritional advice focused on controlling sodium and protein intake (particularly from animal sources)	Usual care (Stage 2 to 3 CKD patients followed-up every 6 months<br>and stage 4 CKD patients followed-up every 3 months)
Kankarn (2019b), Thailand	Included dietitian (nutritional education which aimed at managing CKD and avoiding malnutrition), pharmacist (medication education), physical therapist (exercise education and program), village health volunteer who visited participant home and coordinated with multidisciplinary team, social worker, care giver and community care network also involved.	Usual care (Stage 2 to 3 CKD patients followed-up every 6 months<br>and stage 4 CKD patients followed-up every 3 months)

Kelly (2020), Australia	Telehealth intervention plus workbook involving two phases: Phase 1 (0 - 3 months): individualised telephone-based coaching from a dietitian focused on healthy eating and lifestyle every two weeks for three months with weekly text messages. Phase 2 (3 - 6 months): tailored text messages at frequency requested by participant.	Phase 1 (0 - 3 months): Received workbook + usual care. Phase 2 (3 - 6 months): non-tailored text messages
Kirkman (2019)/Kirkman (2021), US	Supervised aerobic exercise (cycling, walking/jogging, or elliptical) 3 times/week. Duration progressively increased to 45 min at 60 – 85% heart rate reserve	Usual care
Leehey (2009), US	Individualised walking program 3 x weekly starting at 30mins and increased by 5mins every 2 weeks. Supervised for first 6 weeks then home based with weekly phone calls to collect step counts.	No exercise program
Leehey (2016), US	Exercise 3 x week aerobic and resistance training (90mins duration) for first 12 weeks supervised then 40 weeks home based (3 x 60mins or 6 x 30mins) with weekly phone calls and monthly meetings with trainer. Nutritional counselling at baseline with 9 follow-up telephone calls during the study. Referral to a lifestyle modification program including instruction on weight loss and increasing physical activity.	Nutritional counselling at baseline with 9 follow-up telephone calls during the study. Referral to a lifestyle modification program including instruction on weight loss and increasing physical activity.
Li (2020), Taiwan	Provided with wearable devices collecting exercise and dietary data. Participants received diet, exercise, and self-management education by researchers trained by a dietitian. Daily target of 7500 steps set. Mobile app used to encourage participants and allow participants to ask questions on CKD management.	Routine care
Lin (2021), China	Six-week health coaching intervention conducted by nurse, including four 60 min face-to-face sessions (including motivational interviewing to determine motivation, identification of risk factors in participants' lifestyle, establishment of behavioral modification goals and action plans) and two 15 min follow-up telephone sessions to follow-up on action plan and overcome barriers).	pay-for-performance program managed by a health education nurse using a CKD case management recording sheet

Martínez - Villaescusa (2022), Spain	Personalised nutritional intervention aimed at less dietary restriction (promoting vegetables and fibre)	Usual care
MDRD (Tangri (2011)/Kopple (1997), US	0.58g protein/kg/day, 5 - 10mg phosphorus/kg/day•••	1.3g protein/kg/day, 16 - 20 mg phosphorus/kg/day•••
Mekki (2010), Algeria	Mediterranean diet (modified from the National Kidney Foundation - Kidney Disease Outcomes Quality Initiative guidelines): advised to consume olive oil and nuts for seasonings, whole grains, fruits, vegetables and fish. List of foods rich in salt, potassium and phosphorus provided.	Nutritional advice based on the National Kidney Foundation - Kidney Disease Outcomes Quality Initiative guidelines with energy intake: 0.12MJ/kg/BW/day, protein intake: 0.75 g /kg/BW/day, lipid intake: 35% and carbohydrates:55% of total energy intake.
Meloni (2002), Italy	Low protein diet of 0.6g/kg/d. Dietitian provided participants with optimal self-management nutritional education	Maintain usual protein intake
Meloni (2004), Italy	1. Diabetic intervention group received a low protein diet of 0.8g/kg/d. Dietitian provided pts with optimal self-management nutritional education<br>2. Non-diabetic intervention group received a low protein diet 0.6g/kg/d. Dietitian provided pts with optimal self-management nutritional education	Free protein intake
Moe (2011), US	Diet with protein from plant sources: isocaloric (2200 kcal) diet, containing 1000 mg of calcium and 3000mg of sodium per day. Protein and total phosphorus concentration were equivalent, with a target of 800 mg/d of phosphorus.	Protein from animal sources: Isocaloric (2200 kcal) diet, containing 1000 mg of calcium and 3000mg of sodium per day. Protein and total phosphorus concentration were equivalent, with a target of 800 mg/d of phosphorus.

Montoya (2016), US	6 x 1 monthly visits (including physical examination by nephrologist in month 0,3&6). Education sessions at each visit conducted by a specialist (including physician, dietitian, pharmacist, social worker) in the topic area with written information (topics included CKD basics, diet, medication adherence, putting affairs in order, and options for end-stage kidney disease)	Standard care with nephrologist visits every 3 months based on CKD guidelines. Patients were referred to CKD education classes provided by a dialysis vendor for one to two sessions.
Mozaffari-Rad (2022), Iran	Low phosphorous diet limited to 12 mg/kg ideal body weight/day.	No education on reducing phosphorous intake
Mustata (2011), Canada	Exercise program of 2 days/weekly supervised and 3 days/week home- based exercise. Exercise was at 40-60% VO^2 max starting between 5-10mins and increasing to a maximum of 60mins during the study.	Standard care
Nguyen (2018), Vietnam	12-week self-management intervention delivered by experienced nurse teacher. The intervention<br>involved a CKD booklet and a handout, one face-to-face session and two brief follow-up sessions. Sessions aimed at improving CKD knowledge and self-management guided by social cognitive theory	Usual care (brief verbal information about taking medications, reducing salt, smoking cessation, and reducing alcohol consumption)
Otobe (2021), Japan	60 mins group-based exercise once per week (incorporating stretching, resistance training, balance, and aerobic exercises), with at home exercises twice or more per week	Usual care
Paes-Barreto (2013), Brazil	Nutrition education program (in addition to the standard dietary counseling), including individual class, hands-on session, and written materials including recipes. Education including protein and sodium sources, portion sizes, sodium content of cured and packaged foods, written recipes	Standard dietary counselling including an individualized reduced sodium dietary plan based on the National Kidney Foundation Kidney Disease Outcomes Quality Initiative Nutrition Guidelines with protein intake between 0.6 to 0.75g/kg/day or up to 0.8g/kg/day for diabetics with energy intake 25 to 35ckal/kg/day

Rahimimoghadam (2018), Iran	Classical Pilates exercise regime. Exercises conducted in small groups 3 times per week, duration initially 45 mins long, gradually increased to 70 mins	CKD routine care
Rossi (2014), US	Participants completed supervised cardiovascular, resistance and stretching exercises 2 x weekly. Cardiovascular exercises were at 60-65% of predicted max HR with a goal of increasing to 60mins continual exercise. Outside of supervised exercise session participants were encouraged to walk 5,000 - 10,000 steps/day.	Usual care
Sanchez (2009), Spain	Individualised low protein diet of 0.6g/kg/d, energy of 30 - 35kcal/kg/day, low sodium, potassium, phosphates, saturated fat and refined sugar. Diet prescribed by dietitian and adjusted to meet individual needs and preferences	Low protein diet prescribed prior to study
Saran (2017), US	Low sodium diet <2g sodium/day through dietary counselling using motivational interviewing techniques	Usual diet with stable intake of total and saturated fat, potassium intake of 2-3g/day and phosphorus intake of <=1g/day
Sevick (2012), US	Group counselling sessions (weekly for months 1 &2 then biweekly during months 3&4 then monthly for months 5&6) guided by Social Cognitive Theory and supported by technology for dietary self-monitoring using food composition information. Individualised dietary energy targets provided with macronutrient targets of 55% energy from carbohydrates, 30% fats & 15% protein.	Monthly contact with study team, 3 group seminars with general diabetes education and stress management and cooking demonstrations.
Shi (2014), China	Tai Chi 30 mins 3-5 x week with 10mins warm up and 5 mins cool down either side.	no Tai Chi
Slagman (2011), Netherlands	1. Low sodium diet - target sodium intake 50 mmol Na+/day, with lisinopril 40 mg/day<br>2. Low sodium diet - target sodium intake 50 mmol Na+/day, with lisinopril 40 mg/day and valsartan 320 mg/day	1. Regular sodium diet - target sodium intake 200 mmol Na+/day with lisinopril 40 mg/day<br>2. Regular sodium diet - target sodium intake 200 mmol Na+/day with lisinopril 40

		mg/day and valsartan 320 mg/day
Soroka (1998), Israel	Low protein diet of 0.75g/kg/d provided by soy-based products and 1 egg 3/ week. Energy content ~35kcal/kg/day.	Low protein diet of 0.75g/kg/d provided by 50% animal products. Energy content approx 35kcal/kg/day.
St Jules (2022), US	14 group-based sessions with dietitian, immediately followed by 1 hour group behavioural counselling sessions based on Social Cognitive Theory. Diet and exercise prescription included 500kcal/day energy deficit, 150 min/week moderate physical activity, sodium intake <1500mg/day, avoiding processed foods containing phosphorous based food additives. In addition, pts directed to log all food and beverage intake, physical activity, and body weight. Food logs provided nutrient composition data to align with education sessions. Participants also received personalised feedback reports from dietitian.	14 group-based sessions with dietitian. Diet and exercise prescription included 500kcal/day energy deficit, 150 min/week moderate physical activity, sodium intake <1500mg/day, avoiding processed foods containing phosphorous based food additives. In addition, pts directed to log all food and beverage intake, physical activity, and body weight. Food logs provided nutrient composition data to align with education sessions. Participants also received personalised feedback reports from dietitian.
Tang (2017), China	One to one exercise education and guidance provided 3 times while patients in hospital. Home based aerobic exercise program 3 x week, 20-30 mins with warm up and cooldown.	Usual care
Teng (2021), Taiwan	Usual care plus six 30 min face-to-face lifestyle modification sessions with a nurse. Sessions were based on trans theoretical model and tailored to participant stage of change	Usual care plus 10 - 20 minutes face-to-face counselling (verbal information on maintaining therapeutic regimen, reducing salt, smoking cessation, and reducing alcohol intake)

Thompson (2022), Canada	Aimed to increase activity to 150 min/week at moderate intensity. Consisted of 8 weeks of once/week supervised sessions and twice/week home-based sessions, followed by 16 weeks of home-based training	Usual care
Tuot (2019), US	1. Provider intervention: electronic health record-enabled CKD registry tool<br>2. Patient intervention: CKD-SMS program based on SCT components. Program delivered over 1 year by health coaches. Program included: language-concordant<br>low-literacy written materials mailed at months 1, 4, 8; language concordant and culturally tailored automated telephone self-management program with 26 modules delivered fortnightly on kidney health topics; telephone-based health coaching (delivered by lay bilingual health coaches)<br>3. Both 1) and 2)	Usual care
Turban (2021), US	Higher K diet (meals provided): diet providing 100mmol/day K, similar to DASH diet. Nutrients based on KDOQI guidelines and provided 15% energy as protein, 50% energy as carbohydrate, 37% energy as fat (<10% energy as sat fat). Dietary goals at 2000 kcal level: 1 g phosphorous/day and 145 mmol sodium/day.	Lower K diet (meals provided): diet providing 40mmol/day K (similar to higher K diet, but with low K fruit and vegetables). Nutrients based on KDOQI guidelines and provided 15% energy as protein, 50% energy as CHO, 37% energy as fat (<10% energy as sat fat). Dietary goals at 2000 kcal level: 1 g phosphorous/day and 145 mmol sodium/day.
Uchiyama (2021)/Adachi (2022), Japan	Home-based aerobic exercise 3 times per week (40 - 60% peak heart rate) and resistance training 2 times per week (target was 70% of one-repetition maximum)	Usual care
Van Craenenbroeck (2015), Belgium	Home based exercise program Exercise was 4 x 10min cycling per day on a home exercise bike at 90% anaerobic threshold.	Usual care
Williams (2012), Australia	Self-monitoring of blood pressure plus individualised medication review and management plan including motivational interviewing and education, led by a kidney nurse	Usual care

Wu (2018), Taiwan	Self-management intervention focused on self-efficacy, involving small group intervention weekly for four weeks. Intervention meetings ran for 100 mins, consisting of 90 min group discussion and 10 min video	Usual care
Yamagata (2016), Japan	Bimonthly education material and consultation reminders, 30 mins education sessions from dietitians every 3 months and patient data regarding targets and treatment plans	Standard CKD treatment

†Intervention and control details columns only report where differences between intervention and control arms existed (for example if both groups were on a standard CKD diet, this is not reported)

Item S6: Risk of bias assessment summary

Study ID	Method of randomisation	Allocation concealment	Blinding of participants and personnel	Blinding of outcome assessment (objective outcomes)	Blinding of outcome assessment (subjective outcomes)	Incomplete outcome data	Selective outcome reporting	Other bias
Aoike (2015)	?	?	?	+	NA	+	?	+
Aoike (2017)	+	?	?	+	?	-	-	+
Barcellos (2018)	+	+	?	+	+	-	+	+
Baria (2014)	?	?	?	+	NA	+	?	+
Beetham (2022)	+	?	?	+	NA	-	-	+
Caldiroli (2022)	+	?	?	+	NA	-	+	+
Campbell (2008)	+	+	?	NA	?	-	?	+
Castaneda (2001)	?	?	?	+	NA	+	?	+
Chilelli (2015)	+	?	?	+	NA	+	?	+
Clark (2013)	+	?	+	+	+	+	-	-
Correa (2020)/Correa (2021)/Deus (2021)/Dues (2022)	+	?	?	+	NA	-	-	+
de Brito-Ashurst (2013)	+	-	?	+	NA	-	-	+
Eidemak (1997)	?	?	?	+	NA	+	?	+
Facchini (2003)	?	+	?	+	NA	-	?	+
Flesher (2011)	?	?	?	+	NA	-	?	?
Fogelfeld (2017)	?	?	?	+	NA	-	-	+
Goraya (2014)	?	?	?	+	NA	+	?	+
Grazioli (2022)	?	?	?	+	NA	+	?	?
Greenwood (2014)	+	?	?	+	NA	-	-	+
Hamidianshirazi (2022)	+	?	?	+	?	-	+	+
Headley (2012)	?	?	?	+	NA	-	?	+

Headley (2014)/Headley (2017)/Miele (2017)	+	?	?	+	?	+	+	+
Hiraki (2017)	+	?	?	+	NA	-	-	+
Hotu (2010)	?	?	?	+	NA	+	-	-
Hwang (2014)	+	?	?	+	NA	+	-	+
Ihle (1989)	?	?	?	+	NA	+	?	+
Ikizler (2018)	+	?	?	+	NA	+	?	+
Joboshi (2016)	+	-	?	+	NA	+	?	+
Johns (2020)	+	?	?	+	?	+	+	-
Kankarn 2019a	+	?	?	+	NA	+	?	+
Kankarn 2019b	+	?	?	+	NA	+	?	+
Kelly (2020)	+	+	?	+	-	+	+	-
Kirkman (2019)/Kirkman (2020)	+	?	?	+	NA	-	+	+
Leehey (2009)	+	?	?	+	NA	-	?	?
Leehey (2016)	+	?	?	+	?	-	-	+
Li (2020)	?	?	?	+	-	-	?	+
Lin (2020)	+	+	?	NA	-	+	?	-
Martínez-Villaescusa (2022)	?	?	?	+	NA	-	?	+
MDRD	+	+	?	+	NA	+	+	+
Mekki (2010)	?	?	?	+	NA	+	?	?
Meloni (2002)	?	?	?	+	NA	+	?	+
Meloni (2004)	+	?	?	+	NA	+	?	+
Moe (2011)	?	?	-	+	NA	+	?	+
Montoya (2016)	+	?	?	+	?	-	?	+
Mozaffari-Rad (2022)	+	+	?	+	NA	-	-	-
Mustata (2011)	+	+	?	+	?	+	?	+
Nguyen (2018)	+	+	?	+	?	+	?	+
Otobe (2021)	+	?	?	+	NA	-	-	-
Paes-Barreto (2013)	+	-	?	+	NA	-	?	+
Rahimimoghadam (2018)	+	?	?	NA	?	+	?	+
Rossi (2014)	+	?	?	NA	?	-	+	-

Sanchez (2009)	?	?	?	+	NA	-	?	+
Saran (2017)	?	+	-	+	NA	+	-	-
Sevick (2012)	+	?	?	+	NA	?	+	?
Shi (2014)	?	?	?	+	NA	+	?	+
Slagman (2011)	+	?	+	+	NA	+	?	+
Soroka (1998)	?	?	-	+	NA	-	?	?
St Jules (2022)	+	?	-	+	NA	-	-	+
Tang (2017)	+	?	?	NA	?	+	?	+
Teng (2021)	?	?	?	+	?	+	-	+
Thompson (2022)	+	+	-	+	NA	+	-	+
Tuot (2019)	+	+	?	+	-	+	-	-
Turban (2021)	+	?	+	+	NA	-	-	+
Uchiyama (2021)/Adachi (2022)	+	+	-	+	-	+	+	-
Van Craenenbroeck (2015)	?	+	?	+	?	-	-	+
Williams (2012)	+	?	?	+	NA	+	-	+
Wu (2018)	+	?	?	+	?	-	?	+
Yamagata (2016)	+	?	?	+	NA	?	+	+

Item S7: Justification for risk of bias judgements

Study ID	Method of randomisation	Support	Allocation concealment	Support	Blinding of participants and personnel	Support	Blinding of outcome assessment (objective outcomes)	Support	Blinding of outcome assessment (subjective outcomes)	Support
Aoike (2015)	unclear	Study design stated to be randomised, method of randomisation not specified	unclear	Not stated	unclear	Not possible to blind personnel, unclear if pts blinded	low	Not stated, however outcomes objective and unlikely to be affected by blinding	NA	NA
Aoike (2017)	low	"The patients were assigned to exercise or controlgroups after a blocked randomization procedure using arandom block of 6 participants."	unclear	Not stated	unclear	Not possible to blind personnel, unclear if pts blinded	low	Not stated, however outcomes objective and unlikely to be affected by blinding	unclear	Not stated, but suspectible as subjective
Barcellos (2018)	low	"The randomization sequence was obtained using a randomly generated list, in fixed-size blocks of six individuals" - implies computer generated	low	"researchers responsible for randomization were concealed to group assignment"	unclear	Not possible to blind personnel, unclear if pts blinded	low	From protocol paper: "Interviewers will be blinded to the interventions or control status of each patient."	low	From protocol paper: "Interviewers will be blinded to the interventions or control status of each patient."

Baria (2014)	unclear	Study design stated to be randomised, method of randomisation not specified	unclear	Not stated	unclear	Not possible to blind personnel, unclear if pts blinded	low	Not stated, however outcomes objective and unlikely to be affected by blinding	NA	NA
Beetham (2022)	low	computer random assignment program	unclear	not stated	unclear	Not possible to blind personnel, unclear if pts blinded	low	Not stated, although outcomes unlikely to be influenced by blinding	NA	NA
Caldiroli (2022)	low	"For randomization we tossed a coin (heads–LP group, tails–NP group)"	unclear	Not stated	unclear	Described as 'open-label', however unclear whether this refers to only personnel or both personnel and participants	low	Implied to not have been blinded, however outcomes unlikely to be influenced by blinding	NA	NA
Campbell (2008)	low	Patients were randomised toreceive either individual counselling with fortnightly tele-phone follow-up, or standard care (written material only),allocated via a computer-generated number sequence,which was concealed to the recruiting officer (seeFigure 1)	low	Patients were randomised toreceive either individual counselling with fortnightly tele-phone follow-up, or standard care (written material only),allocated via a computer-generated number sequence,which was concealed to the recruiting officer (seeFigure 1)	unclear	Not possible to blind personnel, unclear if pts blinded	NA	NA	unclear	Not stated, but suspectible as subjective
Castaneda (2001)	unclear	Stated as randomised, method of randomisation not stated	unclear	Not stated	unclear	Not possible to blind personnel, unclear if pts blinded	low	Observers were blinded to study group assignment at all times, except during assessments	NA	NA

								of post intervention muscle strength		
Chilelli (2015)	low	by simple randomization using a computer-generated ran-dom binary list;	unclear	Not stated	unclear	Not possible to blind personnel, unclear if pts blinded	low	Not stated, however outcomes objective and unlikely to be affected by blinding	NA	NA
Clark (2013)	low	Participants were randomised in block sizes of three bycomputer-generated randomisation to the hydrationgroup or the control group (2:1), stratified by gender.	unclear	Blinding stated, but allocated concealment not	low	The random allocation was concealed to patients, their healthcare providers and research staff	low	The random allocation was concealed to patients, their healthcare providers and research staff	low	The random allocation was concealed to patients, their healthcare providers and research staff
Correa (2020)/Correa (2021)/Deus (2021)/Deus (2022)	low	www.randomization.com used	unclear	Not stated	unclear	Not possible to blind personnel, unclear if pts blinded after allocation	low	Not stated, although outcomes unlikely to be influenced by blinding	NA	NA
de Brito-Ashurst (2013)	low	Randomisation totreatment was conducted by the study statistician usingcomputer-generated random blocks with block sizes betweenfour and eight and the group assignment given to the researcher	high	Randomisation to treatment was conducted by the study statistician using computer-generated random blocks with block sizes between four and eight and the group assignment given to the researcher - suggests allocation not concealed	unclear	This was a dietary behaviour intervention, thus, neither partici-pants nor the dietitian administering the intervention could beblinded to treatment allocation, parallel design so unclear if pts aware.	low	This was a dietary behaviour intervention, thus, neither partici-pants nor the dietitian administering the intervention could beblinded to treatment allocation. Data analysis was conducted bythe study statistician who was blinded to treatment allocation.	NA	NA

Eidemak (1997)	unclear	Randomisation method not stated	unclear	Not stated	unclear	Not possible to blind personnel, unclear if pts blinded	low	Not stated, however outcomes objective and unlikely to be affected by blinding	NA	NA
Facchini (2003)	unclear	Randomisation method not stated	low	Randomization and concealed patient allocation to either treatment arm was performedby staff personnel blinded to the aim of the study.	unclear	Not possible to blind personnel, unclear if pts blinded	low	Not stated, although outcomes unlikely to be influenced by blinding	NA	NA
Flesher (2011)	unclear	Study design stated to be randomised, method of randomisation not specified	unclear	Not stated	unclear	Not possible to blind personnel, unclear if pts blinded	low	Not stated, although outcomes unlikely to be influenced by blinding	NA	NA
Fogelfeld (2017)	unclear	Study design stated to be randomised, method of randomisation not specified	unclear	Not stated	unclear	Not possible to blind personnel, unclear if pts blinded	low	Not stated, although outcomes unlikely to be influenced by blinding	NA	NA
Goraya (2014)	unclear	Study design stated to be randomised, method of randomisation not specified	unclear	Not stated	unclear	Not possible to blind personnel, unclear if pts blinded	low	Not stated, although outcomes unlikely to be influenced by blinding	NA	NA
Grazioli (2022)	unclear	States: "Patients randomly placed in groups A or B followed…" indicating randomisation, but does not state method	unclear	Not stated	unclear	Not possible to blind personnel, unclear if pts blinded	low	Not stated, although outcomes unlikely to be influenced by blinding	NA	NA
Greenwood (2014)	low	computerised randomised procedure	unclear	Not stated	unclear	Not possible to blind personnel, unclear if pts blinded	low	A researcher blinded to patient group allocation conducted all assessments and patients were asked not to reveal their group allocation	NA	NA

Hamidianshirazi (2022)	low	"Block randomization method was done using online software applications, given a four sequences block size with 30 possible block combination of A or B, to assign participants to either group A or B."	unclear	Not stated	unclear	Stated to be "non-blinded", but not clear if this also applied to participants	low	Not stated, although outcomes unlikely to be influenced by blinding	Unclear	Unclear if participants were blinded (participant reported outcome)
Headley (2012)	unclear	Study design stated to be randomised, method of randomisation not specified	unclear	Not stated	unclear	Not possible to blind personnel, unclear if pts blinded	low	Not stated, although outcomes unlikely to be influenced by blinding	NA	NA
Headley (2014)/Headley (2017)/Miele (2017)	low	An on-line random generator (www.randomization.com) was used to determine the allocation of 50 participants into 25 blocks	unclear	Not stated	unclear	Not possible to blind personnel, unclear if pts blinded	low	Not stated, although outcomes unlikely to be influenced by blinding	unclear	Not stated, but suspectible as subjective
Hiraki (2017)	low	computer-based table of random numbers with treatment allocation ratio of 1:1	unclear	Not stated	unclear	Not possible to blind personnel, unclear if pts blinded	low	Not stated, although outcomes unlikely to be influenced by blinding	NA	NA
Hotu (2010)	unclear	Stated as randomised, method not given (protocol states computer generated, but not clear from paper if this was followed)	unclear	Not stated	unclear	Not possible to blind personnel, unclear if pts blinded	low	Unclear if blinded for all measures (stated as blinded for echocardiographic), however unlikely to affect outcomes	NA	NA

Hwang (2014)	low	Eligible participants were randomly assigned (1:1) to either conventional education or intensive education for LSD with computerized block randomization (block size six) andbalanced according to institution and sex	unclear	Not stated	unclear	study states "It was not feasibleto mask participants to allocation, but clinicians were masked to group assignment" - however unclear if clinicians refers to personnel or outcome assessors	low	Not stated (unclear who "clinicians" refers to), although outcomes unlikely to be influenced by blinding	NA	NA
Ihle (1989)	unclear	Stated as randomised, method not given (protocol states computer generated, but not clear from paper if this was followed)	unclear	Not stated	unclear	Not possible to blind personnel, unclear if pts blinded	low	Not stated, although outcomes unlikely to be influenced by blinding	NA	NA
Ikizler (2018)	low	participants were assigned to one of the study arms using a permuted block randomization strategy in a 1:1 ratio.	unclear	Not stated	unclear	Study stated to be 'unblinded', however unclear who this refers to	low	Not stated, however outcomes objective and unlikely to be affected by blinding	NA	NA

Joboshi (2016)	low	Participants were subsequently allocated to the two groups via computer-based stratified permuted-block randomization with two factors:age(<65 or 65years)and the presence or absence of a cohabitant	high	"Research facilities received notification of the allocation results" - suggests that allocation was not concealed	unclear	"Participants were not informed of the allocation grouping until the end of the intervention", however would not be possible to blind personnel	low	Not stated, although outcomes unlikely to be influenced by blinding	NA	NA
Johns (2020)	low	"Adults (aged >21 years) were randomly assigned using a block scheme and stratified by level of estimated glomerular filtration rate" - assumed to be computer generated	unclear	Not stated	unclear	Not possible to blind personnel, unclear if pts blinded after allocation	low	Not stated, although outcomes unlikely to be influenced by blinding	unclear	Unclear if participants were blinded (participant reported outcome)
Kankarn 2019a	low	"randomization occurred at the level of the group practice using a	unclear	States "randomization occurred at the	unclear	Not possible to blind personnel,	low	Unclear if blinded, but outcomes	NA	NA

		computergenerated randomization sequence and concealment"		level of the group practice using a computergenerated randomization sequence and concealment" however unclear what this refers to in terms of allocation concealment		unclear if pts blinded after allocation		unlikely to be affected		
Kankarn 2019b	low	"randomization occurred at the level of the group practice using a computergenerated randomization sequence and concealment"	unclear	States "randomization occurred at the level of the group practice using a computergenerated randomization sequence and concealment" however unclear what this refers to in terms of allocation concealment	unclear	Not possible to blind personnel, unclear if pts blinded after allocation	low	Unclear if blinded, but outcomes unlikely to be affected	NA	NA
Kelly (2020)	low	"The randomization schedule was created in RedCap (15) and participants were allocated on a 1:1 ratio, stratified by recruitment site and diabetes status using a computer generated random number list"	low	"Allocation was concealed from the site investigators. An offsite trial coordinator randomized participant and notified the intervention coach (who had never met the participant and did not work in the same hospital), who in turn notified participants of their allocation"	unclear	Not possible to blind personnel, participants not blinded, however unclear how this affected results as parallel design	low	To maintain blinding of allocation for site investigators, participants were asked not to disclose which group they had been assigned to during their study visits. Site investigators performed all objective clinical measures, whereas serum and urine pathology were performed as part of usual care (using random samples) by local National	high	self-reported surveys completed by participants who were not blinded

								Association of Testing Authorities, Australia (NATA)-accredited laboratories		
Kirkman (2019)/Kirkman (2020)	low	"computer-generated blocked sequence stratified by sex (randomization.com)"	unclear	Not stated	unclear	Not possible to blind personnel, unclear if pts blinded	low	Not stated, although outcomes unlikely to be influenced by blinding	NA	NA
Leehey (2009)	low	After all baselinedata were obtained, if patients were still eligible, they were randomized using a 2 × 2 block randomization scheme to the exercise or control group.	unclear	Not stated	unclear	Not possible to blind personnel, unclear if pts blinded	low	Not stated, although outcomes unlikely to be influenced by blinding	NA	NA
Leehey (2016)	low	eligible patients were randomized using a computer-generated permuted block scheme.	unclear	Not stated	unclear	Not possible to blind personnel, unclear if pts blinded	low	Not stated, although outcomes unlikely to be influenced by blinding	unclear	not stated, but suspectible as subjective
Li (2020)	unclear	Randomisation method not stated	unclear	Not stated	unclear	Not possible to blind personnel, participants not blinded, however unclear how this affected results as parallel design	low	Not blinded, but outcomes unlikely to be affected	high	Participants not blinded

Lin (2020)	low	"An independent research assistant generated a randomization sequence using a free online software program with a block size of four."	low	"The generated random sequence was concealed in sequentially numbered, opaque envelopes until the participants were assigned to either the experimental group or control group"	unclear	Not possible to blind personnel, participants not blinded, however unclear how this affected results as parallel design	NA	NA	high	Participants not blinded
Martínez-Villaescusa (2022)	unclear	Randomisation method not stated	unclear	Not stated	unclear	Not possible to blind personnel, unclear if pts blinded after allocation	low	Not stated, although outcomes unlikely to be influenced by blinding	NA	NA
MDRD	low	Statified randomisation, assume implies low risk approach	low	"The randomization process is centrally administered by the Data Coordinating Center through telephone contact"	unclear	Not possible to blind personnel, unclear if pts blinded	low	Not stated, although outcomes unlikely to be influenced by blinding	NA	NA
Mekki (2010)	unclear	Not stated	unclear	Not stated	unclear	Not possible to blind personnel, unclear if pts blinded	low	Not stated, although outcomes unlikely to be influenced by blinding	NA	NA
Meloni (2002)	unclear	Not stated	unclear	Not stated	unclear	Not possible to blind personnel, unclear if pts blinded	low	Not stated, although outcomes unlikely to be influenced by blinding	NA	NA
Meloni (2004)	low	We performed simple randomization iwthin these 2 groups using	unclear	Not stated	unclear	Not possible to blind personnel,	low	Not stated, although outcomes unlikely to be	NA	NA

		dedicated software generating casual numbers to assign patients to treatment groups. The remaining were placed in the control group				unclear if pts blinded		influenced by blinding		
Moe (2011)	unclear	Not stated	unclear	Not stated	high	Not possible to blind personnel or pts	low	Not stated, although outcomes unlikely to be influenced by blinding	NA	NA
Montoya (2016)	low	"computer randomised group assignment"	unclear	States "participants were then given a written notation of their computer-randomized group assignment" - however unclear if allocation concealed from participants	unclear	Not possible to blind personnel, unclear if pts blinded	low	Not stated, although outcomes unlikely to be influenced by blinding	unclear	not stated, but suspectible as subjective
Mozaffari-Rad (2022)	low	"Participants were randomly assigned to the intervention or control group (1:1ratio) in random blocks of 6 subjects based on the blocked randomization method"	low	"An investigator with no clinical involvement in the study put the trial group codes in opaque envelopes based on the random list and sealed them. Then, the other person, who was not involved in the trial and not aware of random sequences, assigned the patients to the numbered sealed envelopes"	unclear	Not possible to blind personnel, unclear if pts blinded	low	Not stated, although outcomes unlikely to be influenced by blinding	NA	NA
Mustata (2011)	low	"Subjects were randomly assigned to exercise in addition to standard care (EX) or standard care alone (CT) using a	low	"Subjects were randomly assigned to exercise in addition to standard care (EX) or	unclear	Not possible to blind personnel, unclear if pts blinded	low	Not stated (only stated for exercise testing), although outcomes unlikely	unclear	not stated, but suspectible as subjective

		computer-generated randomization list"		standard care alone (CT) using a computer-generated randomization list and sequentially numbered, sealed envelopes."				to be influenced by blinding		
Nguyen (2018)	low	"A random-number table was generated in Microsoft Excel to produce an allocation sequence for the control and intervention groups"	low	"A number was placed inside an opaque sealed envelope according to the random-number table. All sealed envelopes were then mixed and placed into a box. After baseline data were collected, each participant selected an envelope. Allocation concealment was achieved with a trained recruitment research assistant assessing for eligibility, conducting recruitment, collecting baseline data, and supervising participants selecting an envelope. The recruitment research assistant then used the coding sheet to randomly allocate participants into either the control or intervention group."	unclear	Not possible to blind personnel, unclear if pts blinded after allocation	low	"The outcome research assistant did not have access to the coding sheet or the interpretation of the code, was therefore blinded to group allocation."	unclear	Unclear if participants were blinded (participant reported outcome)

Otobe (2021)	low	"randomized to either the exercise or control group using an interactive web response system"	unclear	Not stated	unclear	Described aa 'unblinded', however unclear whether this refers to only personnel or both personnel and participants	Low	"The outcome measure evaluators were blinded to patient allocation at both time-points."	NA	NA
Paes-Barreto (2013)	low	"A randomization list with a number sequence was generated by computer software".	high	"A randomization list with a number sequence was generated by computer software. Patients were randomized to the intervention groups in consecutive order of admission to the study"	unclear	States "allocation group was concealed during study" but unclear who this refers to	low	Not stated, although outcomes unlikely to be influenced by blinding	NA	NA
Rahimimoghadam (2018)	low	"simple allocation using www.random.org"	unclear	Not stated	unclear	Not possible to blind personnel, unclear if pts blinded after allocation	NA	NA	unclear	Unclear if participants were blinded (participant reported outcome)
Rossi (2014)	low	"Participants were stratified by age (<=70 or>70 years ofage) and CKD stage (3 or 4) and randomized to one of two study arms using computer-generated random numbers"	unclear	Not stated	unclear	Not possible to blind personnel, unclear if pts blinded	NA	NA	unclear	not stated, but suspectible as subjective
Sanchez (2009)	unclear	"The patients were divided randomly into two groups" - randomisation method not stated	unclear	Not stated	unclear	Not possible to blind personnel, unclear if pts blinded	low	Not stated, although outcomes unlikely to be influenced by blinding	NA	NA
Saran (2017)	unclear	States "randomization was performed using sealed	low	randomization was performed using	high	Not possible to blind	low	States study investigators were	NA	NA

		envelopes at each site" - however does not define how randomisation sequence generated		sealed envelopes at each site		personnel delivering intervention, or participants		blinded to assigned treatment, also outcomes unlikely to be affected by blinding		
Sevick (2012)	low	Participants were randomized within these apriori defined HbA1c/eGFR strata to either the intervention or attention control groups using computer-generated per-muted blocks	unclear	Not stated	unclear	Not possible to blind personnel, unclear if pts blinded	low	Not stated, although outcomes unlikely to be influenced by blinding	NA	NA
Shi (2014)	unclear	Not stated	unclear	Not stated	unclear	Not possible to blind personnel, unclear if pts blinded	low	Not stated, although outcomes unlikely to be influenced by blinding	NA	NA
Slagman (2011)	low	An independent pharmacist randomised these sequences, using a computer program	unclear	States "We implemented the random allocation sequence by means of sequentially numbered containers of study drug. Physicians enrolled patients, and the pharmacist allocated the study drug sequentially to consecutive participants" - however unclear if allocation concealed	low	"The randomisation code remained secret during the entire study; all participants, investigators, and care providers were blinded, except for the pharmacist." "Diverse professional dietitians	low	"The randomisation code remained secret during the entire study; all participants, investigators, and care providers were blinded, except for the pharmacist."	NA	NA

						gave further dietary counselling in various autonomous dietary practices in the community" - implies care providers blinded				
Soroka (1998)	unclear	Not stated	unclear	Not stated	high	Not possible to blind personnel or pts	low	Not stated, although outcomes unlikely to be influenced by blinding	NA	NA
St Jules (2022)	low	From protocol paper: "Within strata they are randomized using computer-generated permuted blocks with equal allocation"	unclear	Not stated	High	"Because of the nature of behavioral interventions, neither participants nor study staff could subsequently be blinded to group assignment."	low	Protocol paper suggests staff taking outcome measures blinded ("bias is minimized by excluding interventionists from follow-up measurement visit"). In addition, outcomes unlikely to be influenced by blinding	NA	NA
Tang (2017)	low	Subjects who enrolled in the study were sequentially numbered and randomly assigned to the experimental group and the control group by computer-generated random list.	unclear	Not stated	unclear	Not possible to blind personnel, unclear if pts blinded	NA	NA	unclear	not stated, but suspectible as subjective
Teng (2021)	unclear	Paper states: "Upon obtaining written informed consent, subjects were randomly assigned to the control or intervention group at a ratio of 1 to 1 using paper ballots." however unclear	Unclear	Not stated	unclear	Not possible to blind personnel, unclear if pts blinded after allocation	low	Not stated, although outcomes unlikely to be influenced by blinding	unclear	Unclear if participants were blinded (participant reported outcome)

		whether paper ballot system was randomised								
Thompson (2022)	low	From protocol paper: "The randomization sequence will be computer generated using Stata/MP 15.1"	low	"Allocation was concealed by web-based central randomization using The Research Electronic Data Capture System (RedCap; projectredcap.org)."	High	"Due to the nature of the intervention, participants and study staff could not be blinded to group assignment."	low	Unclear if outcome assessors were blinded, although outcomes unlikely to be influenced by blinding	NA	NA
Tuot (2019)	low	"Prior to enrollment, using blind and secure allocation by computer, the study team randomly assigns participants to one study arm using a random number generator"	low	"Results are placed in a sealed randomization envelope by a study coordinator.<br><br>At the study enrollment appointment, the study team obtains written informed consent and collects baseline study measures. At the completion of this baseline visit, patients learn about their randomization assignment by opening the randomization envelope."	unclear	Not possible to blind personnel, participants not blinded, however unclear how this affected results as parallel design	low	"outcome assessors were blinded to randomization"	high	Participants not blinded
Turban (2021)	low	"After run-in, participants were randomized to one of two computer-generated sequences"	unclear	Not stated	low	"Participants and investigators were masked to diet sequence"	low	"Participants and investigators were masked to diet sequence"	NA	NA

Uchiyama (2021)/Adachi (2022)	low	"block randomization with a block size of two was performed by an individual not associated with the trial, using computer-generated random numbers"	low	From protocol document: "The patients will be equally allocated into control and exercise groups, with assignment to either exercise or usual care. Dr. Nakaya, who is not associated with this trial, will be responsible for these processes. Dr. Nakaya will keep the master list of number assignments in a password protected and encrypted laptop"	high	"Blinding of participants and rehabilitation doctors to group assignment was impossible"	low	"nephrologists responsible for the CKD clinic and outcome assessors were blinded"	high	Participants not blinded
Van Craenenbroeck (2015)	unclear	States "patients were randomly assigned in a 1:1 ratio by sealed opaque envelopes to exercise training or usual care" however unclear how randomisation sequence generated	low	States "patients were randomly assigned in a 1:1 ratio by sealed opaque envelopes to exercise training or usual care"	unclear	Not possible to blind personnel, unclear if pts blinded	low	Not stated, although outcomes unlikely to be influenced by blinding	unclear	not stated, but suspectible as subjective
Williams (2012)	low	"Following recruitment, participants were allocated code numbers prior to enrolment and being randomized to the intervention or control group (1:1 ratio) by an off-site statistician"	unclear	Not stated	unclear	Not possible to blind personnel, participants in intervention stated not to be blinded, however unclear re: control group	low	States: "The research assistant was trained to collect data and was blinded to group assignment"	NA	NA
Wu (2018)	low	"randomly assigned into an experimental or control group by using a random number table"	unclear	Not stated	unclear	Not possible to blind personnel, unclear if pts blinded after allocation	low	Not stated, although outcomes unlikely to be influenced by blinding	unclear	Unclear if participants were blinded (participant reported outcome)

Yamagata (2016)	low	Randomization was performed centrally by means of a computer-generated random-number sequence.	unclear	Not stated	unclear	Not possible to blind personnel, unclear if pts blinded	low	Not stated, although outcomes unlikely to be influenced by blinding	NA	NA

Item S8: Summary data for each study

Table 8A: summary data for eGFR (mL/min/1.73^{2})

Study	Intervention	Post-intervention or change from baseline	Mean (intervention)	Standard deviation (intervention)	n (intervention)	Mean (control)	Standard deviation (control)	n (control)
Aoike (2015)	Exercise	Post	31.9	13.7	14	23.9	12.2	15
Aoike (2017)	Exercise	Post	29.288	12.88889	25	24.1	13	15
Barcellos (2018)	Exercise	Change	-1.9	12.17023	58	-2.6	12.18521	49
Baria (2014)	Exercise	Post	30.11111	10.30108	18	25.9	14	9

Beetham (2022)	Multiple	Post	37.1	12.50068	79	37.7	12.43679	81
Caldiroli (2022)	Dietary	Post	18.5	7	14	19	9	13
Castaneda (2001)	Exercise	Change	1.18	12.26977	14	-1.62	11.80911	12
Chilelli (2015)	Dietary	Post	23.85	3.01	13	33.6	4	13
Clark (2013)	Hydration	Change	-0.8	4	17	-1.8	5	11
Correa (2020)/Correa (2021)/Deus (2021)/Deus (2022)	Exercise	Post	48.23	3.591594	70	43.88	2.85	35
de Brito-Ashurst (2013)	Dietary	Change	3	7.146669	25	3.4	5.434375	23
Eidemak (1997)	Exercise	Change	-0.3044	0.5404	15	-0.3127	0.3191	15
Fogelfeld (2017)	Multiple	Post	30.98	15.49	60	28.95	15.06	60
Goraya (2014)	Dietary	Change	-10	1.692121	33	-18.8	1.833131	33
Grazioli (2022) - with functional bar	Exercise	Post	45.7512	14.9818	6	45.7108	11.0192	5
Grazioli (2022) - without functional bar	Exercise	Post	49.7753	21.0212	5	46.8621	4.3653	5
Greenwood (2014)	Exercise	Post	40.3	18.7	8	41.7	18.2	10
Hamidianshirazi (2022)	Dietary	Change	2.8	5.824088	53	-2.7	7.211103	52
Headley (2012)	Multiple	Post	34.2	18	10	53.5	29.9	11
Headley (2014)/Headley (2017)/Miele (2017)	Exercise	Post	52.4	19.1	25	50.1	16.2	21
Hiraki (2017)	Exercise	Post	35.1	11.4	14	39.5	12.9	14
Hotu (2010)	Multiple	Post	33	17	30	41	18	28
Hwang (2014)	Dietary	Post	63.4	10.90871	119	65.1	10.10247	126
Ihle (1989)	Dietary	Post	12	16.70329	31	6	17.23369	33
Ikizler (2018) - Exercise + dietary	Multiple	Post	42.7868	26.3211	24	35.6385	16.6256	7
Ikizler (2018) - Exercise	Exercise	Post	38.7138	15.6913	22	35.6385	16.6256	7
Ikizler (2018) - Dietary	Dietary	Post	38.1601	22.6173	24	35.6385	16.6256	7
Joboshi (2016)	Behaviour	Change	-1.37	4.86	32	-0.49	6.51	29
Kelly (2020)	Dietary	Change	2	7.498127	37	3	8.866524	36
Leehey (2009)	Exercise	Post	39	22	7	41	5.3	4
Leehey (2016)	Exercise	Post	39.6	19.9	14	35.8	20.2	18
Li (2020)	Multiple	Post	72.47	24.28	25	56.69	22.25	24

Martínez-Villaescusa (2022)	Dietary	Post	17.5393	7.4224	36	15.2469	3.5635	21
Mekki (2010)	Dietary	Post	77	4.024922	20	75	35.77709	20
Meloni (2002)	Dietary	Post	38.8	9.6	35	39.3	7.2	34
Meloni (2004) - diabetic (0.8g/kg/d intervention)	Dietary	Post	38.9	9.6	40	39.3	7.2	40
Meloni (2004) - non-diabetic (0.6g/kg/d intervention)	Dietary	Post	41.8	2.4	44	38.3	3.8	45
Montoya (2016)	Multiple	Post	19.3	12.26977	13	22.6	11.80911	13
Mozaffari-Rad (2022)	Dietary	Change	-4.129	14.609	36	-1.468	6.154	35
Otobe (2021)	Exercise	Post	37.2	14.5	23	34.7	13.7	21
Paes-Barreto (2013)	Dietary	Post	33.7	15.6	43	34.1	13.5	46
Sanchez (2009)	Dietary	Post	26.42	7.32	20	25.46	9.99	20
Saran (2017)	Dietary	Post	35.4621	12.4036	29	37.5241	11.9888	29
Shi (2014)	Exercise	Post	51	21.55806	11	43	24.66577	10
Soroka (1998)	Dietary	Post	28.11	3.4	9	29.56	3.8	9
Teng (2021)	Behaviour	Post	54.01	19.63	52	49.44	17.54	52
Thompson (2022)	Exercise	Post	25.84615	13	13	28	11	20
Uchiyama (2021)/Adachi (2022)	Exercise	Post	21.3	6.7	23	22	4.3	23
Van Craenenbroeck (2015)	Exercise	Change	0.74	5.114272	19	-0.33	4.163055	21
Williams (2012)	Behaviour	Post	47.8223	19.688	36	46.03	21.5503	39
Wu (2018)	Behaviour	Post	27.81	13.81	36	26.83	13.3	54

Table 8B: summary data for 24 hr albuminuria (mg/24 hr)

Citation	Intervention	Post-intervention or change from baseline	Mean (intervention)	Standard deviation (intervention)	n (intervention)	Mean (control)	Standard deviation (control)	n (control)
Hwang (2014)	Dietary	Post	178	11.94906	118	231.1	12.24908	124
Saran (2017)	Dietary	Post	100.799	171.8425	29	167.3472	353.5426	29

Uchiyama (2021)/Adachi (2022)	Exercise	post	1307	1724.7	23	1044.5	1508.4	23

Table 8C: summary data for albumin:creatinine ratio (mg/g)

Citation	Intervention	Post-intervention or change from baseline	Mean (intervention)	Standard deviation (intervention)	n (intervention)	Mean (control)	Standard deviation (control)	n (control)
Leehey (2009)	Exercise	Post	305	456	7	221	304	4
Fogelfeld (2017)	Multiple	post	1086.206	1514.608	60	1094.892	1551.068	60
Leehey (2016)	Exercise	post	323.6021	443.1727	14	479.9625	568.7516	18
Saran (2017)	Dietary	Post	66.9412	124.85	29	107.3638	212.4515	29
Tuot (2019) - registry	Behaviour	Post	353.9	432.0421	37	783.6	691.8945	25
Tuot (2019) - without registry	Behaviour	Post	611.2	894.3766	37	697.9	1137.844	38
Thompson (2022)	Exercise	post	687.7588	850.5848	13	509.7733	557.9808	18

Table 8D: summary data for creatinine (mg/dL)

Citation	Intervention	Post-intervention or change from baseline	Mean (intervention)	Standard deviation (intervention)	n (intervention)	Mean (control)	Standard deviation (control)	n (control)
Aoike (2015)	Exercise	Post	2.6	1.1	14	3.2	1.4	15
Aoike (2017)/Gomes (2017)	Exercise	Post	2.8	1.1	24	3.14	1.58	15
Beetham (2022)	Multiple	Post	1.9097	0.655839	79	1.8871	0.638799	81
Castaneda (2001)	Exercise	Post	163.54	1.519299	14	182.1	1.708882	12
Correa (2020)/Correa (2021)/Deus (2021)/Deus (2022)	Exercise	Post	1.76	0.285231	70	1.9	0.31	35
Fogelfeld (2017)	Multiple	Post	2.612	1.3672	60	2.7886	1.899	60
Grazioli (2022) - with functional bar	Exercise	Post	1.5876	0.6281	6	1.4281	0.2458	5
Grazioli (2022) - without functional bar	Exercise	Post	1.4626	0.4408	5	1.2137	0.0932	5
Greenwood (2014)	Exercise	Post	2.11649	0.69608	8	2.30068	1.13565	10
Hamidianshirazi (2022)	Dietary	Change	-0.09	0.728011	53	0.3	0.72111	52
Hotu (2010)	Multiple	Post	2.796411	1.501205	30	2.22723	1.261193	29
Hwang (2014)	Dietary	Post	1.15	11.0178	119	1.13	11.33722	126
Ihle (1989)	Dietary	Post	8.927	13.2123	31	10.509	16.22839	33
Joboshi (2016)	Behaviour	Change	0.12	0.29	32	0.21	0.98	29
Kelly (2020)	Dietary	Change	-0.04	0.305666	35	0.02	0.576324	36
Leehey (2009)	Exercise	Post	2.8	1.2	7	2.1	0.4	4
Li (2020)	Multiple	Post	1.16	0.39	25	1.51	0.81	24

Mekki (2010)	Dietary	Post	1.2317	2.375151	20	1.243	1.667659	20
Meloni (2002)	Dietary	Post	3	0.5	35	2.8	0.3	34
Meloni (2004) - diabetic (0.8g/kg/d intervention)	Dietary	Post	3	0.5	40	2.8	0.3	40
Meloni (2004) - non-diabetic (0.6g/kg/d intervention)	Dietary	Post	2.7	0.3	44	3	0.5	45
Montoya (2016)	Multiple	Post	3.1	1.519299	13	2.8	1.708882	13
Mozaffari-Rad (2022)	Dietary	Change	0.056	0.294	36	0.043	2.162	35
Otobe (2021)	Exercise	Post	1.71	0.79	23	1.46	0.69	21
Paes-Barreto (2013)	Dietary	Post	2.3	1.1	43	2.3	1.2	46
Sanchez (2009)	Dietary	Post	3.31	0.82	20	3.48	1.24	20
Saran (2017)	Dietary	Post	2.0552	0.7716	29	1.9552	0.62	29
Shi (2014)	Exercise	Post	1.1	0.663325	11	1.5	0.948683	10
Slagman (2011) - ACE inhibitor	Dietary	Post	1.6837	0.51857	26	1.5481	0.460951	26
Slagman (2011) - ACE inhibitor + ARB	Dietary	Post	1.7741	0.51857	26	1.5481	0.460951	26
Soroka (1998)	Dietary	Post	3.2	0.32	9	3.2	0.26	9
Thompson (2022)	Exercise	Post	2.804138	1.3334	13	2.6442	1.5481	20
Williams (2012)	Behaviour	Post	1.4886	0.7547	36	1.5573	0.5392	39
Wu (2018)	Behaviour	Post	2.96	2.14	36	3.04	2.17	54

Table 8E: summary data for systolic blood pressure (mmgHg)

Citation	Intervention	Post-intervention or change from baseline	Mean (intervention)	Standard deviation (intervention)	n (intervention)	Mean (control)	Standard deviation (control)	n (control)
Aoike (2015)	Exercise	Post	118.7	7.3	14	126.8	6.7	15
Aoike (2017)	Exercise	Post	117.492	7.653617	25	130.4	7.2	15
Barcellos (2018)	Exercise	Change	-12.1	23.96015	58	-11.1	23.15189	49
Beetham (2022)	Multiple	Post	133	29.01944	79	136	27.13481	81
Correa (2020)/Correa (2021)/Deus (2021)/Deus (2022)	Exercise	Post	132	4.601032	60	146	3	30
de Brito-Ashurst (2013)	Dietary	Change	-8.5743	6.907814	25	0.662651	6.87249	23
Eidemak (1997)	Exercise	Post	131.7209	14.3729	15	137.9905	15.8102	15
Flesher (2011)	Multiple	Post	126.6	17.23056	23	143.7	16.19966	17
Fogelfeld (2017)	Multiple	Post	140.06	18.94	60	140.25	15.64	60
Goraya (2014)	Dietary	Change	-37.5	11.42182	33	-24.2	11.70384	33
Grazioli (2022) - with functional bar	Exercise	Post	131.5743	14.8258	6	128	5.0858	5
Grazioli (2022) - without functional bar	Exercise	Post	136.9022	24.5812	5	134.468	18.224	5
Greenwood (2014)	Exercise	Post	133.2	14.6	8	127.1	17	10
Hamidianshirazi (2022)	Dietary	change	-7.8	11.64818	53	-0.4	9.374433	52
Headley (2012)	Multiple	Post	117.7	11.21	10	123.8	18.7	11
Headley (2014)/Headley (2017)/Miele (2017)	Exercise	Post	124.5	15.9	25	128.4	25.3	21
Hotu (2010)	Multiple	Post	140	19	30	149	23	28
Hwang (2014)	Dietary	Post	121.2	14.18133	119	122.6	14.59246	126
Ihle (1989)	Dietary	Post	132	61.24541	31	129	63.19019	33
Ikizler (2018)	Multiple	Post	121.9394	13.397	24	125.8664	8.2669	7

Ikizler (2018)	Exercise	Post	126.3589	15.0573	22	125.8664	8.2669	7
Ikizler (2018)	Dietary	Post	122.7156	17.2946	25	125.8664	8.2669	7
Joboshi (2016)	Behaviour	Post	140.26	17.65	32	140.03	16.9	29
Kelly (2020)	Dietary	Change	-1	22.81773	38	2	19.49513	37
Kirkman (2019)/Kirkman (2020)	Exercise	Post	136	15.49193	15	147	16	16
Leehey (2009)	Exercise	Post	113	16	7	136	5	4
Leehey (2016)	Exercise	Post	135	18	14	131	18	18
Meloni (2004) - diabetic (0.8g/kg/d intervention)	Dietary	Post	133	9.2	40	135	3.3	40
Meloni (2004) - non-diabetic (0.6g/kg/d intervention)	Dietary	Post	134	7.6	44	136	3.2	45
Montoya (2016)	Multiple	Post	130.4	17.23056	13	128.8	16.19966	13
Mozaffari-Rad (2022)	Dietary	Change	-2.239	12.582	36	-3.772	21.719	35
Nguyen (2018)	Behaviour	Post	127.57	15.33	68	132.05	14.15	67
Saran (2017)	Dietary	Post	127.3069	16.7493	29	131.3828	15.7209	29
Shi (2014)	Exercise	Post	129	53.066	11	149	60.08328	10
Slagman (2011) - ACE inhibitor	Dietary	Post	123	10.19804	26	134	15.29706	26
Slagman (2011) - ACE inhibitor + ARB	Dietary	Post	121	15.29706	26	131	15.29706	26
St Jules (2022)	Behaviour	change	-4	19	51	-2	15	46
Thompson (2022)	Exercise	Post	150	23	11	131	15	13
Tuot (2019) - registry	Behaviour	Post	124.56	16.49588	37	129.14	16.9582	25
Tuot (2019) - without registry	Behaviour	Post	131.91	23.99401	37	127.27	22.81773	38
Turban (2021)	Dietary	Post	118.6	12.4	25	122.8	9.2	25
Uchiyama (2021)/Adachi (2022)	Exercise	post	142.3	18.7	23	144	19.9	23
Van Craenenbroeck (2015)	Exercise	Change	-3.58	17.26196	19	-8.88	10.19344	21
Williams (2012)	Behaviour	Change	136	17.23056	36	148	16.19966	39
Wu (2018)	Behaviour	Post	137.84	18.83	36	144	22.74	54
Yamagata (2016)	Multiple	Post	135.5	4.132662	48	135.1	4.132662	48

Table 8F: summary data for diastolic blood pressure (mmgHg)

Citation	Intervention	Post-intervention or change from baseline	Mean (intervention)	Standard deviation (intervention)	n (intervention)	Mean (control)	Standard deviation (control)	n (control)
Aoike (2015)	Exercise	post	76.1	4.4	14	81	3.7	15
Aoike (2017)	Exercise	post	74.768	5.377323	25	83.8	6.7	15
Barcellos (2018)	Exercise	change	-5.8	13.88167	58	-6.3	13.22965	49
Beetham (2022)	Multiple	post	78	15.62585	79	79	15.82864	81
Correa (2020)/Correa (2021)/Deus (2021)/Deus (2022)	Exercise	post	78	4.809947	60	92	5	30
de Brito-Ashurst (2013)	Dietary	change	-5.04016	9.729316	25	-1.10442	5.758032	23
Eidemak (1997)	Exercise	post	82.8279	4.3119	15	83.6884	2.8746	15
Flesher (2011)	Multiple	post	69.4	11.48257	23	74.5	10.67634	17
Fogelfeld (2017)	Multiple	post	71.56	14.03	60	74.96	11.71	60
Grazioli (2022) - with functional bar	Exercise	post	76.0849	11.7046	6	77.5447	5.0858	5
Grazioli (2022) - without functional bar	Exercise	post	77.0042	8.9001	5	72.5447	1.6953	5
Greenwood (2014)	Exercise	post	84.4	14.7	8	78.9	6.9	10
Hamidianshirazi (2022)	Dietary	change	-3.3	8.736132	53	1.08	10.09554	52
Headley (2012)	Multiple	post	74.5	5.1	10	73.8	14.9	11
Headley (2014)/Headley	Exercise	post	77.8	10.6	25	75.3	14.7	21

(2017)/Miele (2017)									
Hotu (2010)	Multiple	post	78	11	30	77	12	28
Hwang (2014)	Dietary	post	73.6	9.817841	119	74.8	10.10247	126
Ihle (1989)	Dietary	post	84	44.54211	31	87	45.9565	33
Ikizler (2018) - Exercise + dietary	Multiple	post	75.6116	9.8507	24	76.6221	8.2669	7
Ikizler (2018) - Exercise	Exercise	post	79.0645	9.9853	22	76.6221	8.2669	7
Ikizler (2018) - Dietary	Dietary	post	76.3578	11.7918	25	76.6221	8.2669	7
Joboshi (2016)	Behaviour	post	74.23	9.01	32	76.37	8.66	29
Kelly (2020)	Dietary	change	-1	12.16945	38	0	9.006197	37
Kirkman (2019)/Kirkman (2020)	Exercise	post	80	11.61895	15	86	8	16
Leehey (2009)	Exercise	post	65	10	7	77	8	4
Meloni (2004) - diabetic (0.8g/kg/d intervention)	Dietary	post	83	7.4	40	83.6	5.1	40
Meloni (2004) - non-diabetic (0.6g/kg/d intervention)	Dietary	post	81.2	7.1	44	81.4	4.8	45
Montoya (2016)	Multiple	post	76.9	11.48257	13	70.3	10.67634	13
Mozaffari-Rad (2022)	Dietary	change	-1.848	9.524	36	3.455	17.325	35
Nguyen (2018)	Behaviour	post	79.69	9.87	68	81.85	10.14	67
Saran (2017)	Dietary	post	69.3862	9.3496	29	70.7207	9.7341	29
Shi (2014)	Exercise	post	80	33.16625	11	92	34.78505	10
Slagman (2011) - ACE inhibitor	Dietary	post	73	10.19804	26	80	10.19804	26
Slagman (2011) - ACE inhibitor + ARB	Dietary	post	71	10.19804	26	77	10.19804	26

St Jules (2022)	Behaviour	change	-2.3	12	51	0	11	46
Thompson (2022)	Exercise	post	76	14	11	70	13	13
Turban (2021)	Dietary	post	65.1	7.2	25	65.2	8.8	25
Uchiyama (2021)/Adachi (2022)	Exercise	post	78.5	16.3	23	76.9	12.7	23
Van Craenenbroeck (2015)	Exercise	change	-3.41	13.09171	19	-1.88	7.930675	21
Williams (2012)	Behaviour	change	69	11.48257	36	75	10.67634	39
Wu (2018)	Behaviour	post	75.97	13.63	36	74.2	13.38	54
Yamagata (2016)	Multiple	post	75.8	3.099497	48	76.4	2.582914	48

Table 8G: summary data for body weight (kg)

Citation	Intervention	Post-intervention or change from baseline	Mean (intervention)	Standard deviation (intervention)	n (intervention)	Mean (control)	Standard deviation (control)	n (control)
Aoike (2015)	Exercise	Post	82.3	13	14	84.4	11.4	15
Barcellos (2018)	Exercise	Change	-0.9	2.281919	58	-0.2	9.676391	50
Baria (2014)	Exercise	Post	87.52222	16.96569	18	85.9	7.7	9
Beetham (2022)	Multiple	Post	92	17.85812	79	98	20.35111	81
Caldiroli (2022)	Dietary	Post	72.2705	19.3579	14	75.815	16.1128	13
Castaneda (2001)	Exercise	Change	0.46	2.6	14	-3.21	1.5	12
Correa (2020)/Correa (2021)/Deus (2021)/Deus (2022)	Exercise	Post	97.755	9.981827	70	101.4	9.49	35
Facchini (2003)	Dietary	Post	76	14	91	78	14	79
Goraya (2014)	Dietary	Post	80.2	5.1	36	81.2	6	36
Grazioli (2022) - with functional bar	Exercise	Post	79.4891	11.4315	6	75.6745	10.4682	5
Grazioli (2022) - without functional bar	Exercise	Post	75.3945	11.7397	5	70.9127	5.2977	5
Greenwood (2014)	Exercise	Post	72.9	16.1	8	85.6	12.7	10
Hamidianshirazi (2022)	Dietary	Change	-0.04	0.728011	53	-0.2	0.72111	52
Headley (2012)	Multiple	Post	82.4	17.6	10	93.6	14.8	11
Ihle (1989)	Dietary	Post	54.1	12.66327	31	59.1	14.14469	33
Ikizler (2018)	Multiple	Post	93.2667	20.5683	24	103.4881	39.7729	7
Ikizler (2018)	Exercise	Post	90.2689	15.7705	22	103.4881	39.7729	7
Ikizler (2018)	Dietary	Post	96.6312	24.6055	25	103.4881	39.7729	7
Kelly (2020)	Dietary	Change	-1.6	4.198951	37	-0.1	3.694385	36
Kirkman (2019)/Kirkman (2020)	Exercise	Post	88.3	14.08	12	96.74	20.74	14

Leehey (2009)	Exercise	Post	115	23	7	136	20	4
Li (2020)	Multiple	Post	76.25	15.52	25	76.8	13.27	24
MDRD - females	Dietary	Post	69.3	13.7	111	72.2	14.9	102
MDRD - males	Dietary	Post	83.2	12.8	168	88.5	14.6	181
Meloni (2002)	Dietary	Post	60.8	7.3	35	60.3	11.7	34
Meloni (2004) - diabetic (0.8g/kg/d intervention)	Dietary	Post	61.4	12.66327	40	65	7.4	40
Meloni (2004) - non-diabetic (0.6g/kg/d intervention)	Dietary	Post	65.6	3.5	44	64	7.2	45
Montoya (2016)	Multiple	Post	83.68779	12.66327	13	88.17836	14.14469	13
Mozaffari-Rad (2022)	Dietary	Change	-0.79	7.93	36	-0.48	6.07	35
Paes-Barreto (2013)	Dietary	Post	72.4	17.5	43	72.8	15.9	46
Sanchez (2009)	Dietary	Post	74.85	12.4	20	76.84	16.2	20
Saran (2017)	Dietary	Post	93.4828	18.7904	29	95.1828	19.9734	29
Slagman (2011) - ACE inhibitor	Dietary	Post	87	10.19804	26	89	15.29706	26
Slagman (2011) - ACE inhibitor + ARB	Dietary	Post	87	10.19804	26	89	10.19804	26
St Jules (2022)	Behaviour	Change	-2.7	4.4	51	-2.3	3.4	45
Teng (2021)	Behaviour	Post	74.97	9.31	52	78.39	15.13	52
Thompson (2022)	Exercise	Post	96.39	22.4	10	85.6	21.1	13
Uchiyama (2021)/Adachi (2022)	Exercise	Post	66.6	16.3	23	61.3	12.9	23

Table 8H: summary data for HbA1c (%)

Citation	Intervention	Post-intervention or change from baseline	Mean (intervention)	Standard deviation (intervention)	n (intervention)	Mean (control)	Standard deviation (control)	n (control)
Aoike (2015)	Exercise	Post	6.07	0.96	14	6.03	1.01	15
Aoike (2017)	Exercise	Post	5.948	0.689879	25	6.1	1	15
Beetham (2022)	Multiple	Post	6.9	1.546357	79	6.9	1.789495	81
Correa (2020)/Correa (2021)/Deus (2021)/Deus (2022)	Exercise	Change	-0.543	0.864498	70	0.001	0.183	35
Facchini (2003)	Dietary	Post	7.91	1.067382	91	7.29	1.124106	79
Fogelfeld (2017)	Multiple	Post	7.42	1.66	60	7.82	1.71	60
Hotu (2010)	Multiple	Post	8	1.9	30	7.9	1.7	28
Ikizler (2018)	Multiple	Post	5.5212	0.4947	20	5.8189	0.6889	7
Ikizler (2018)	Exercise	Post	5.7723	0.485	17	5.8191	0.7151	6
Ikizler (2018)	Dietary	Post	5.5642	0.3951	23	5.8191	0.7151	6
Leehey (2009)	Exercise	Post	8.3	2.4	7	8.1	3.7	4
Leehey (2016)	Exercise	Post	7.9	2.3	14	7.4	1.2	18
Meloni (2002)	Dietary	Post	6	1.1	35	6.2	0.8	34
Meloni (2004) - diabetic	Dietary	Post	6	1.1	40	6.2	0.8	40

Otobe (2021)	Exercise	Post	5.9358	0.3951	23	5.5078	0.5565	21
Sevick (2012)	Behaviour	Change	0.5	0.7	16	0.5	1	16
St Jules (2022)	Behaviour	Change	-0.3	0.9	51	-0.3	0.9	46
Thompson (2022)	Exercise	Post	7.3	1.1	7	7.2	1	7
Uchiyama (2021)/Adachi (2022)	Exercise	Post	6.3	1.8	23	6.1	0.8	23
Williams (2012)	Behaviour	Post	7	0.0001	36	7	1.53931	39
Wu (2018)	Behaviour	Post	7.13	1.28	36	7	1.42	54
Yamagata (2016)	Multiple	Post	7.06	0.344389	48	7.18	0.378827	48

Item S9: Forest plots

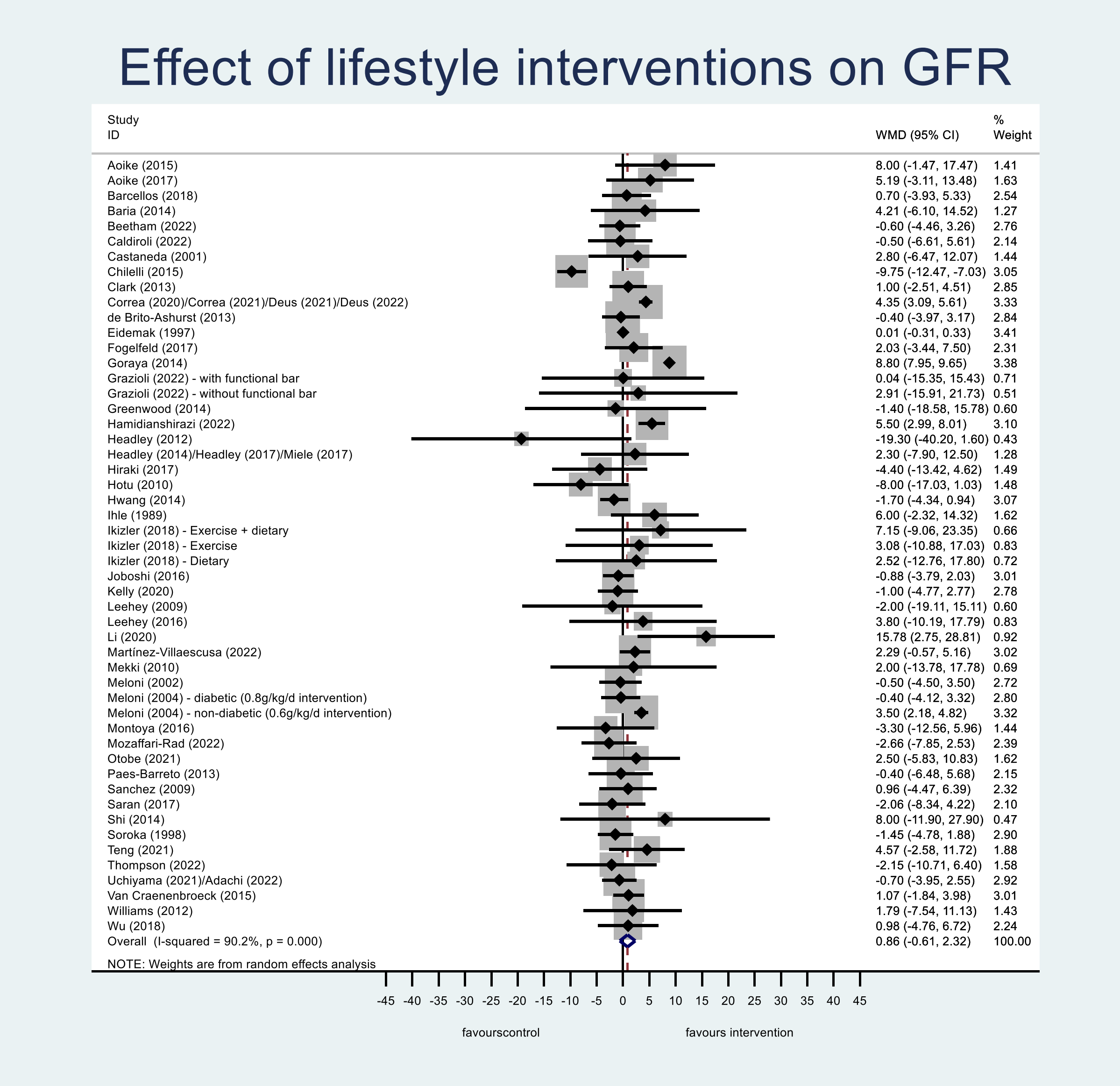

Figure 9A: Difference in eGFR (mL/min/1.73^2) between lifestyle intervention and control. Diamond indicates weighted mean difference with 95% confidence intervals.

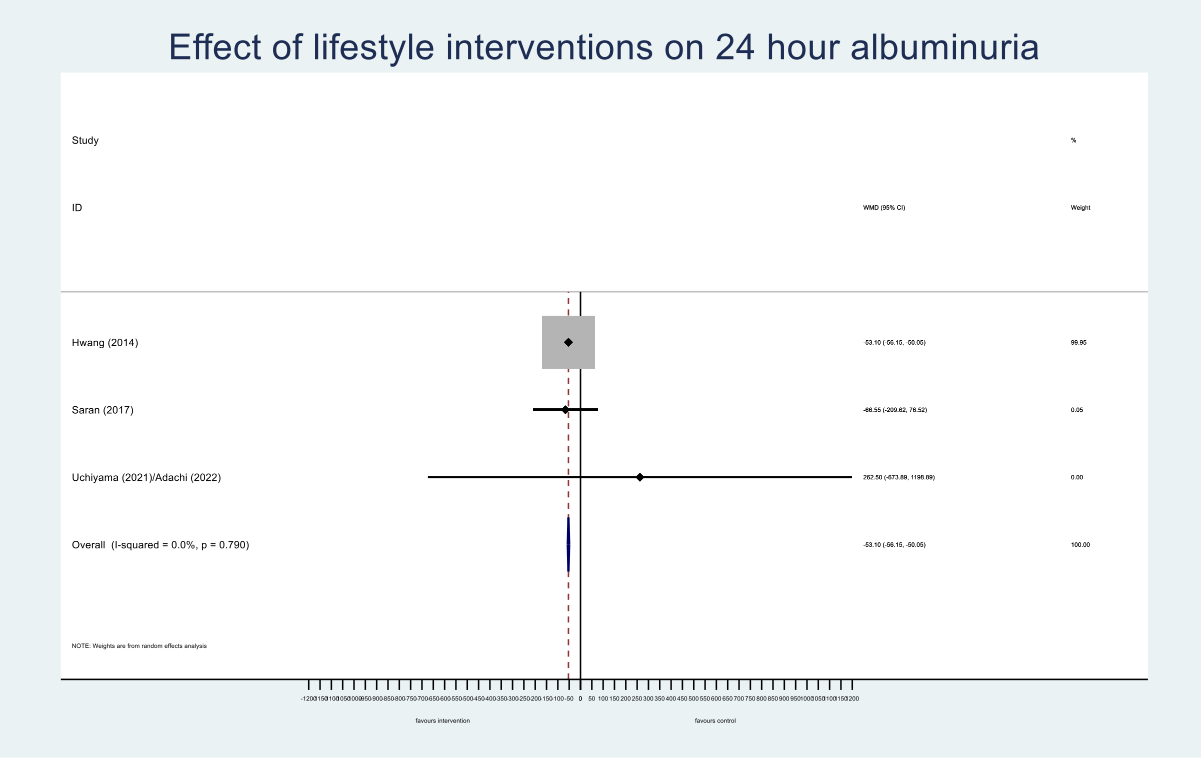

Figure 9B: Difference in 24 hour albuminuria (mg/24 hr) between lifestyle intervention and control. Diamond indicates weighted mean difference with 95% confidence intervals.

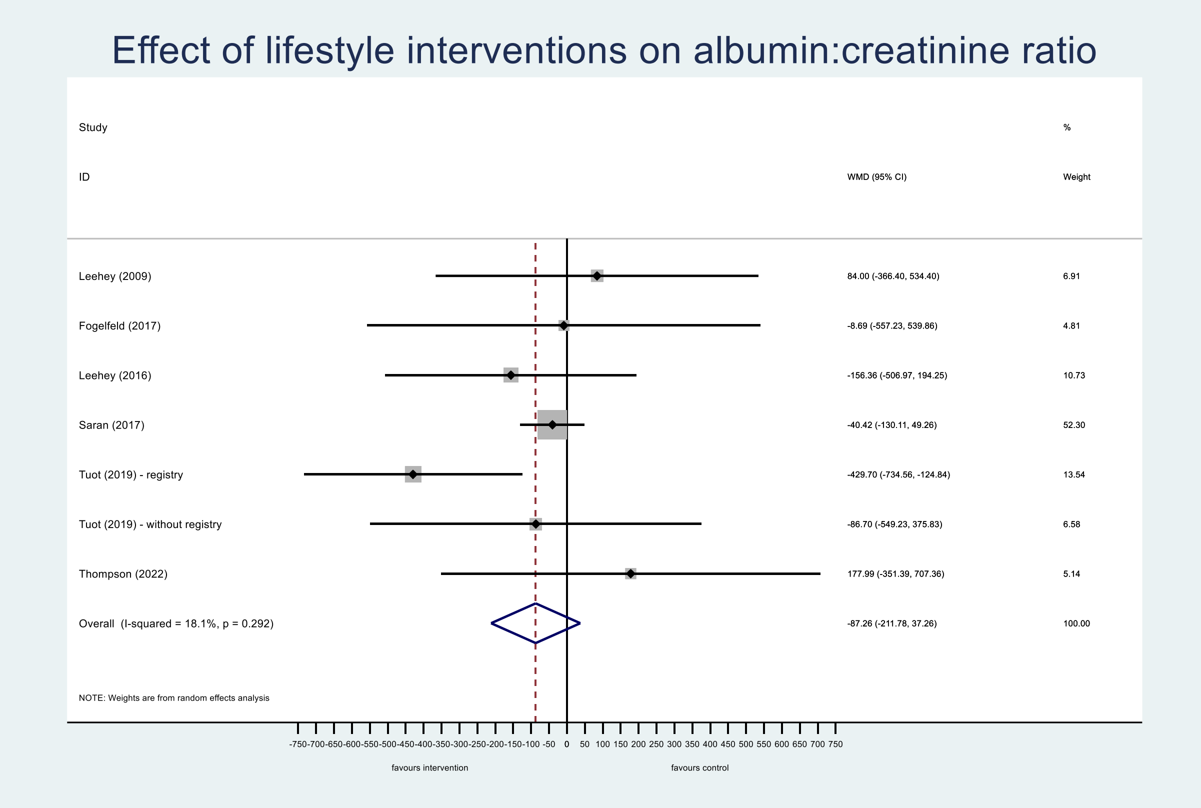

Figure 9C: Difference in albumin:creatinine ratio (mg/g) between lifestyle intervention and control. Diamond indicates weighted mean difference with 95% confidence intervals.

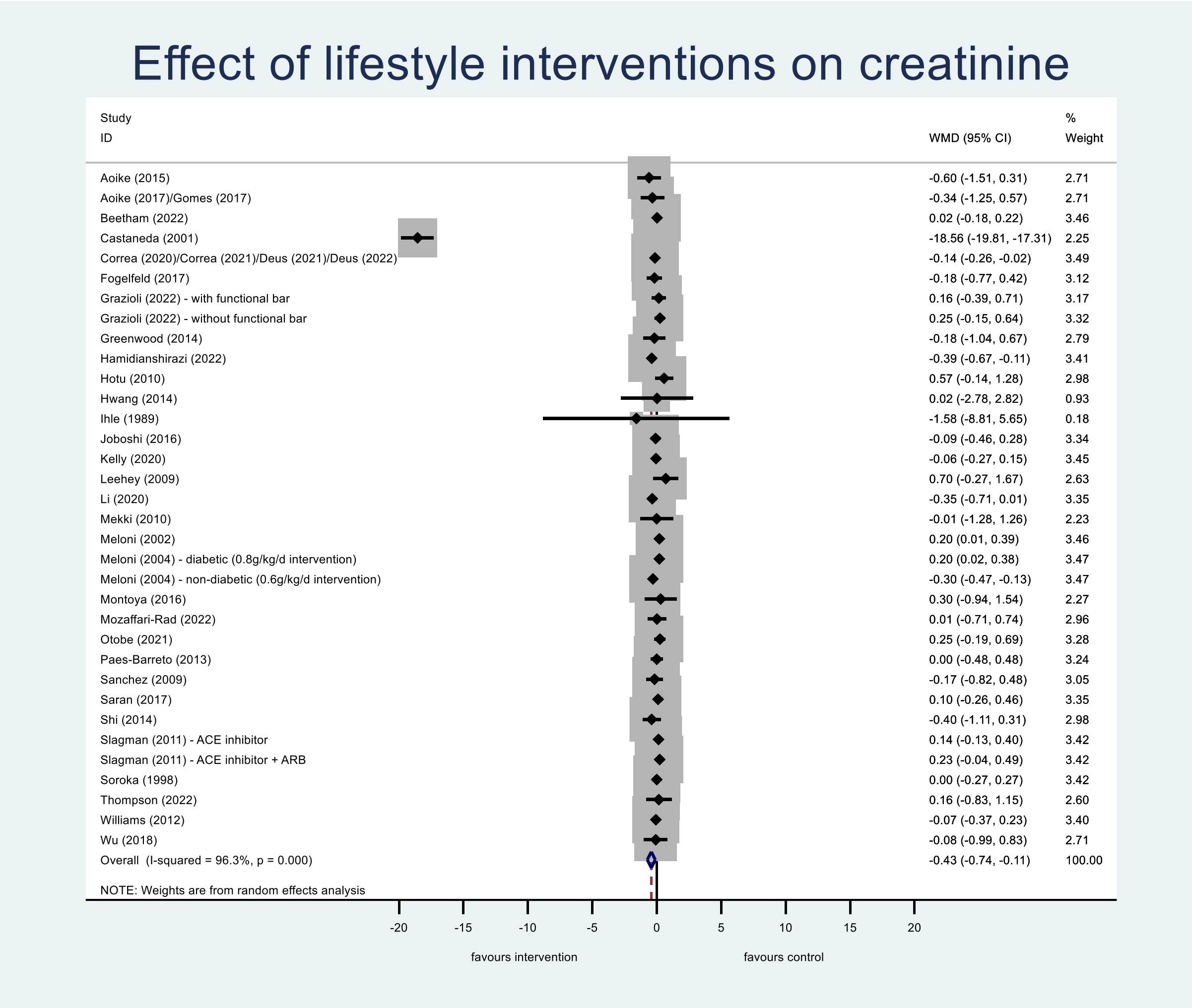

Figure 9D: Difference in creatinine (mg/dL) between lifestyle intervention and control. Diamond indicates weighted mean difference with 95% confidence intervals.

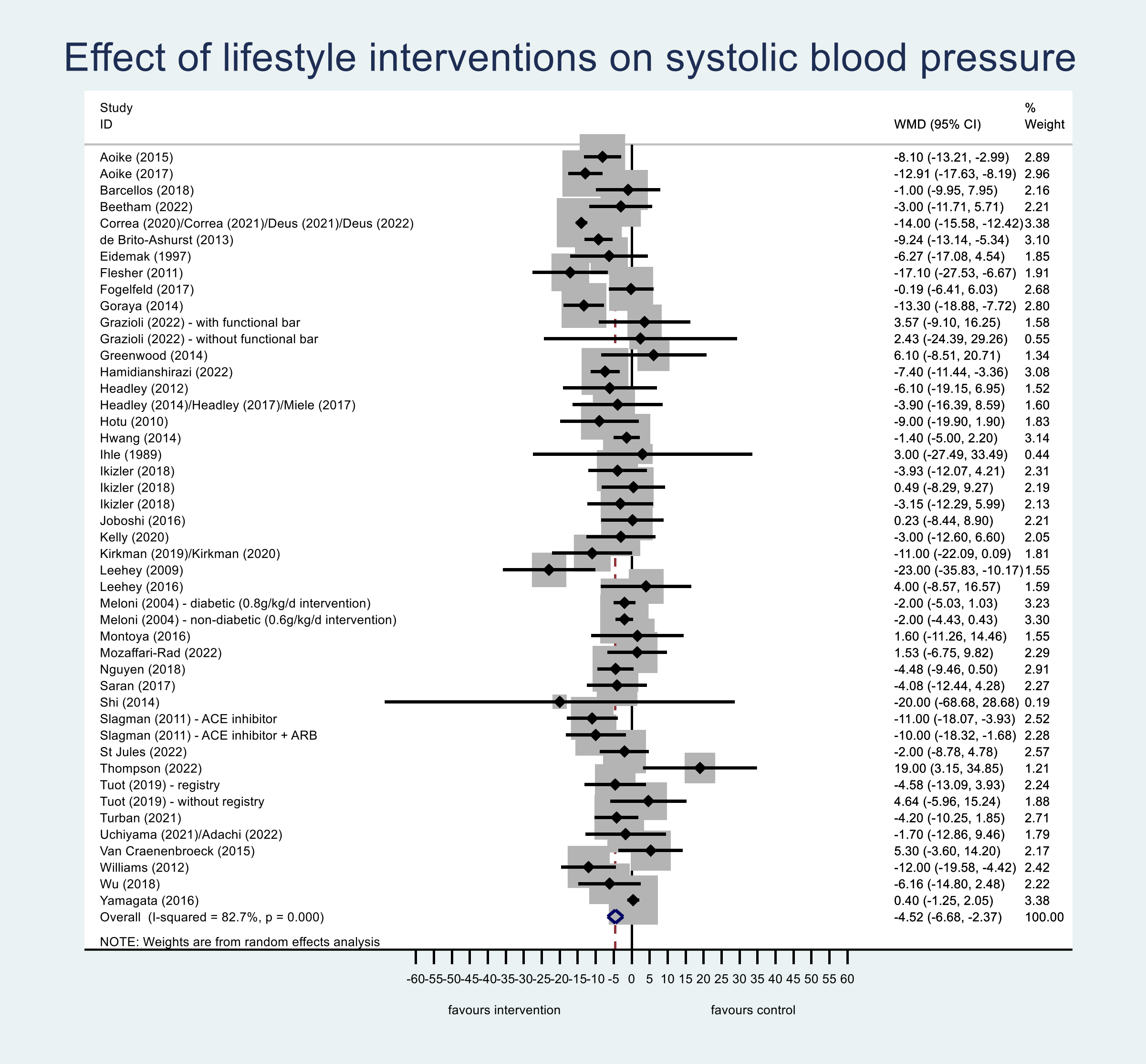

Figure 9E: Difference in systolic blood pressure (mmHg) between lifestyle intervention and control. Diamond indicates weighted mean difference with 95% confidence intervals.

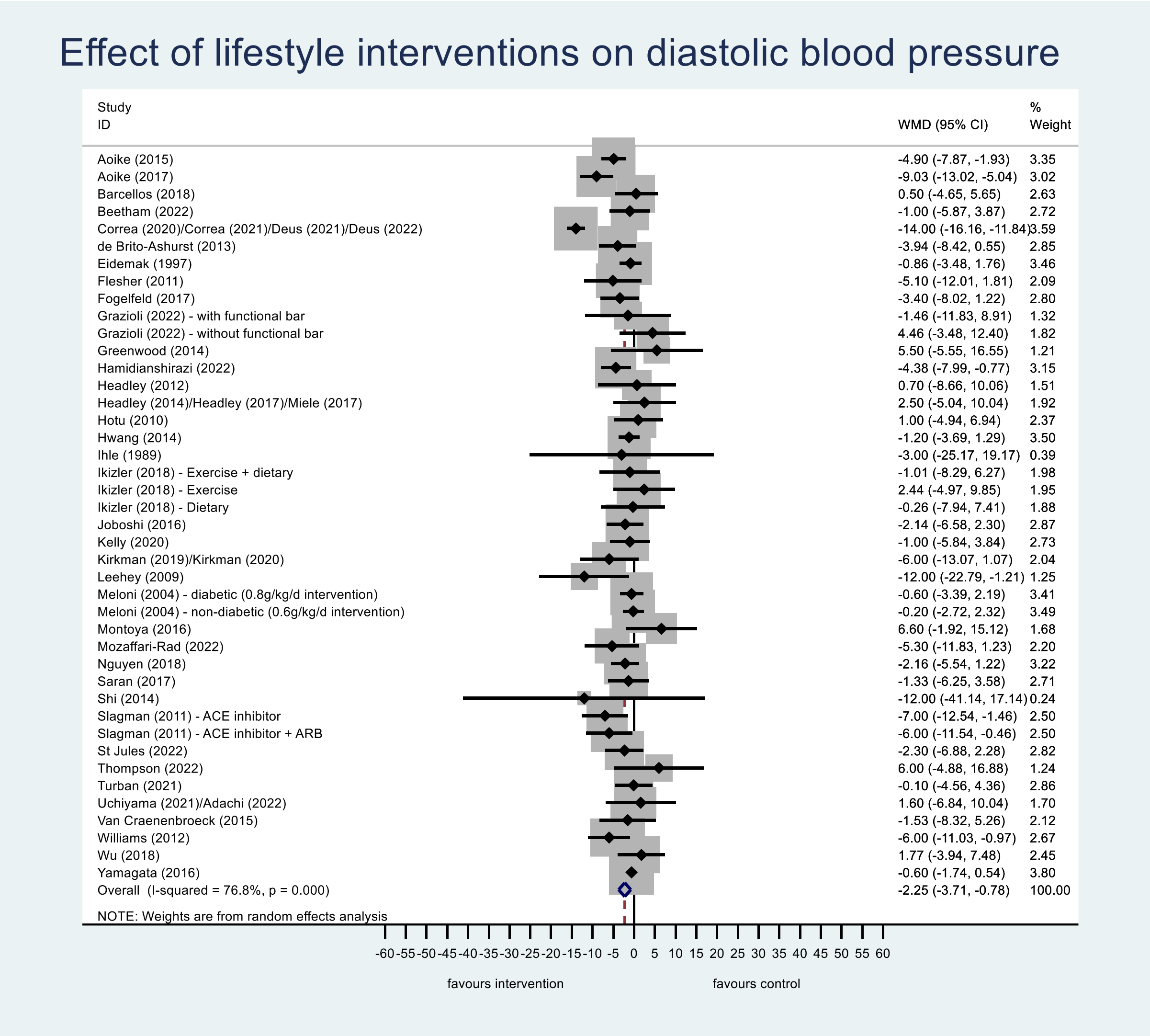

Figure 9F: Difference in diastolic blood pressure (mmHg) between lifestyle intervention and control. Diamond indicates weighted mean difference with 95% confidence intervals.

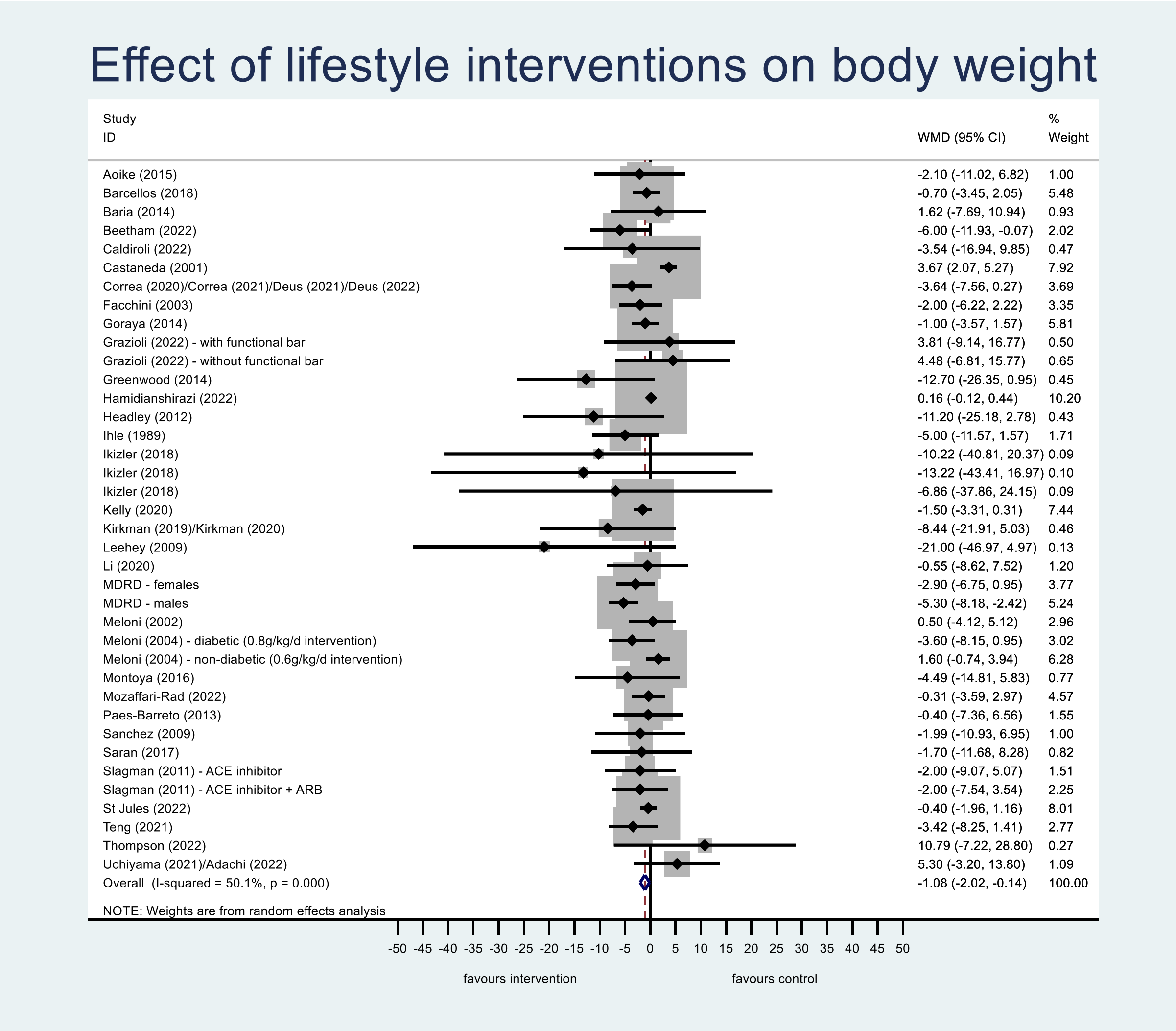

Figure 9G: Difference in body weight (kg) between lifestyle intervention and control. Diamond indicates weighted mean difference with 95% confidence intervals.

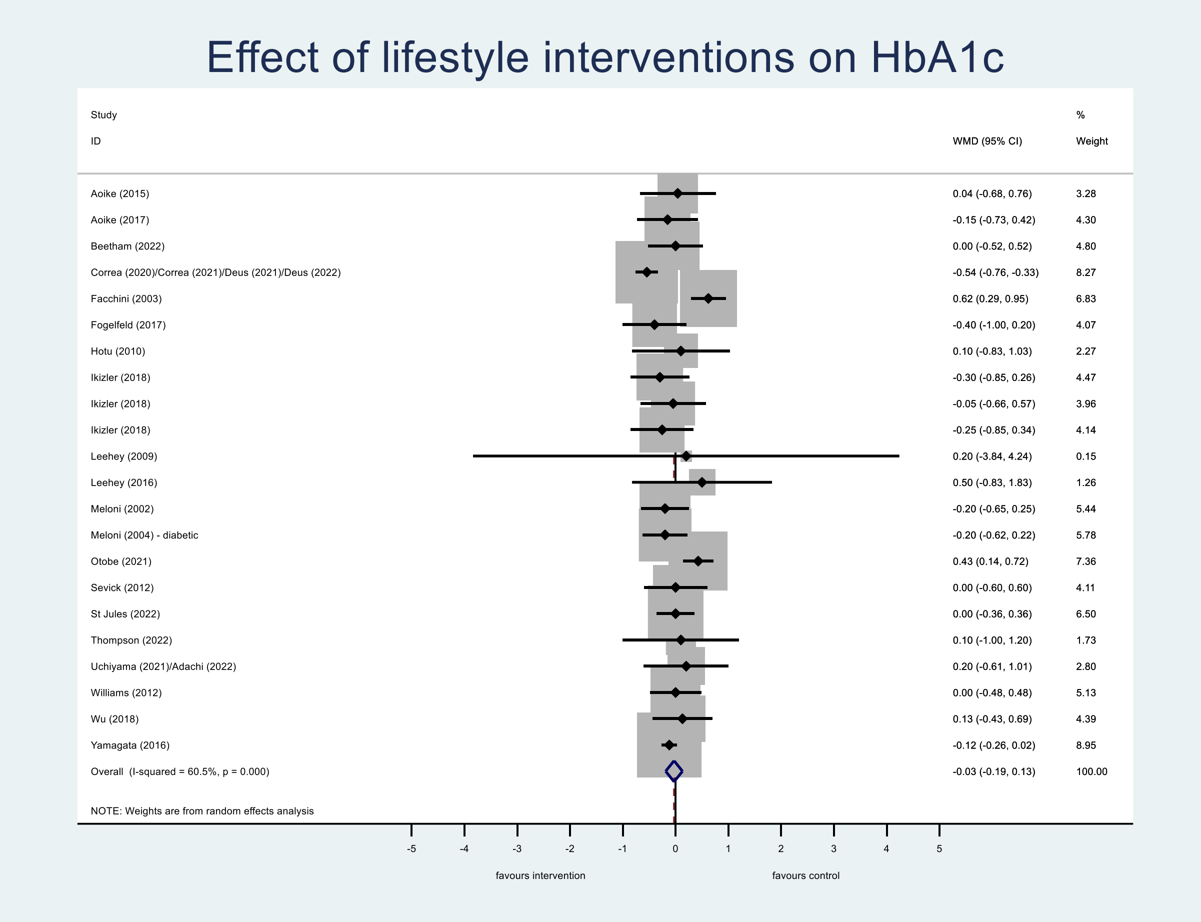

Figure 9H: Difference in HbA1c (%) between lifestyle intervention and control. Diamond indicates weighted mean difference with 95% confidence intervals.

Item S10: Sensitivity analyses using correlation coefficient of 0.25, 0.5, and 0.75 for cross-over studies

Table 10A: sensitivity analyses using varying correlation coefficients for cross-over studies, eGFR (mL/min/1.73^2)

**Outcome**	**Pooled effect**	**Inconsistency (I^2)**
**Paired analysis (original analysis)**	0.857 (-0.606, 2.320), p=0.251*	90.2%
**Correlation coefficient: 0.25**	0.938 (-0.426, 2.302), p=0.178†	95.1%
**Correlation coefficient: 0.5**	0.929 (-0.430, 2.288), p=0.180†	95.1%
**Correlation coefficient: 0.75**	0.917 (-0.431, 2.265), p=0.183†	95.1%

*Weighted mean difference (95% confidence intervals)
† Effect size (95% confidence intervals)

Table 10B: sensitivity analyses using varying correlation coefficients for cross-over studies, 24 hour albuminuria (mg/24hr)

**Outcome**	**Pooled effect**	**Inconsistency (I^2)**
**Paired analysis (original analysis)**	-53.103 (-56.151, -50.054), p<0.001*	0%
**Correlation coefficient: 0.25**	-53.100 (-55.256, -50.945), p<0.001†	0%
**Correlation coefficient: 0.5**	-53.102 (-55.257, -50.946), p<0.001†	0%
**Correlation coefficient: 0.75**	-53.104 (-55.259, -50.949), p<0.001†	0%

*Weighted mean difference (95% confidence intervals)
† Effect size (95% confidence intervals)

Table 10C: sensitivity analyses using varying correlation coefficients for cross-over studies, albumin:creatinine ratio (mg/g)

**Outcome**	**Pooled effect**	**Inconsistency (I^2)**
**Paired analysis (original analysis)**	-87.261 (-211.778, 37.255), p=0.170*	18.1%
**Correlation coefficient: 0.25**	-91.715 (-239.312, 55.881), p=0.223†	62.1%
**Correlation coefficient: 0.5**	-91.690 (-237.920, 54.539), p=0.219†	62.7%
**Correlation coefficient: 0.75**	-91.663 (-236.387, 53.061), p=0.214†	63.4%

*Weighted mean difference (95% confidence intervals)
† Effect size (95% confidence intervals)

Table 10D: sensitivity analyses using varying correlation coefficients for cross-over studies, creatinine (mg/dL)

Outcome	Pooled effect	Inconsistency (I^2)
**Paired analysis (original analysis)**	-0.426 (-0.740, -0.111), p=0.008*	96.3%
**Correlation coefficient: 0.25**	-0.494 (-0.808, -0.180), p=0.002†	98.2%
**Correlation coefficient: 0.5**	-0.490 (-0.797, -0.183), p=0.002†	98.2%
**Correlation coefficient: 0.75**	-0.480 (-0.768, -0.191), p=0.001†	98.2%

*Weighted mean difference (95% confidence intervals)
† Effect size (95% confidence intervals)

Table 10E: sensitivity analyses using varying correlation coefficients for cross-over studies, systolic blood pressure (mmHg)

Outcome	Pooled effect	Inconsistency (I^2)
**Paired analysis (original analysis)**	-4.524 (-6.683, -2.365), p<0.001*	82.7%
**Correlation coefficient: 0.25**	-4.271 (-6.342, -2.200), p<0.001†	91.2%
**Correlation coefficient: 0.5**	-4.294 (-6.349, -2.240), p<0.001†	91.2%
**Correlation coefficient: 0.75**	-4.326 (-6.347, -2.305), p<0.001†	91.3%

*Weighted mean difference (95% confidence intervals)
† Effect size (95% confidence intervals)

Table 10F: sensitivity analyses using varying correlation coefficients for cross-over studies, diastolic blood pressure (mmHg)

Outcome	Weighted mean difference (95% CI)	Inconsistency (I^2)
**Paired analysis (original analysis)**	-2.247 (-3.713, -0.781), p=0.003*	76.8%
**Correlation coefficient: 0.25**	-2.042 (-3.520, -0.564), p=0.007†	89.4%
**Correlation coefficient: 0.5**	-2.058 (-3.523, -0.593), p=0.006†	89.5%
**Correlation coefficient: 0.75**	-2.080 (-3.519, -0.641), p=0.005†	89.8%

*Weighted mean difference (95% confidence intervals)
† Effect size (95% confidence intervals)

Table 10G: sensitivity analyses using varying correlation coefficients for cross-over studies, body weight (kg)

Outcome	Weighted mean difference (95% CI)	Inconsistency (I^2)
**Paired analysis (original analysis)**	-1.076 (-2.016, -0.136), p=0.025*	50.1%
**Correlation coefficient: 0.25**	-1.448 (-2.366, -0.529), p=0.002†	75.3%
**Correlation coefficient: 0.5**	-1.454 (-2.365, -0.543), p=0.002†	75.4%
**Correlation coefficient: 0.75**	-1.464 (-2.361, -0.567), p=0.001†	75.7%

*Weighted mean difference (95% confidence intervals)
† Effect size (95% confidence intervals)

Item S11: Sensitivity analyses exploring the impact of different analysis scenarios (where applicable)

Table 11A: sensitivity analyses excluding studies with imputed standard deviations

Outcome	Original scenario, weighted mean difference (95% CI), I^2	Sensitivity analysis, weighted mean difference (95% CI), I^2
**eGFR (mL/min/1.73^2)**	0.857 (-0.606, 2.320), 90.2%	0.890 (-0.598, 2.379), 90.6%
**Creatinine (mg/dL)**	-0.426 (-0.740, -0.111), 96.3%	-0.014 (-0.099, 0.072), 41.2%
**Systolic blood pressure (mmHg)**	-4.524 (-6.683, -2.365), 82.7%	-4.179 (-6.401, -1.957), 83.3%
**Diastolic blood pressure (mmHg)**	-2.247 (-3.713, -0.781), 76.8%	-2.234 (-3.753, -0.716), 77.6%
**Body weight (kg)**	-1.076 (-2.016, -0.136), 50.1%	-0.886 (-1.843, 0.072), 50.4%
**HbA1c (%)**	-0.030 (-0.189, 0.130), 60.5%	-0.089 (-0.236, 0.057), 45.2%

Table 11B: sensitivity analyses excluding cluster randomised controlled trial (Yamagata et al., 2016)

Outcome	Original scenario, weighted mean difference (95% CI), I^2	Sensitivity analysis, weighted mean difference (95% CI), I^2
**Systolic blood pressure (mmHg)**	-4.524 (-6.683, -2.365), 82.7%	-4.738 (-6.844, -2.632), 77.5%
**Diastolic blood pressure (mmHg)**	-2.247 (-3.713, -0.781), 76.8%	-2.276 (-3.853, -0.698), 75.2%
**HbA1c (%)**	-0.030 (-0.189, 0.130), 60.5%	-0.020 (-0.205, 0.166), 62.1%

Table 11C: sensitivity analyses pooling intervention groups of Ikizler et al. (2018)

Outcome	Original scenario, weighted mean difference (95% CI), I^2	Sensitivity analysis, weighted mean difference (95% CI), I^2
**eGFR (mL/min/1.73^2)**	0.857 (-0.606, 2.320), 90.2%	0.846 (-0.623, 2.315), 90.6%
**Systolic blood pressure (mmHg)**	-4.524 (-6.683, -2.365), 82.7%	-4.615 (-6.817, -2.412), 83.4%
**Diastolic blood pressure (mmHg)**	-2.247 (-3.713, -0.781), 76.8%	-2.324 (-3.815, -0.832), 77.9%
**Body weight (kg)**	-1.076 (-2.016, -0.136), 50.1%	-1.099 (-2.052, -0.146), 52.9%

**HbA1c (%)**	-0.030 (-0.189, 0.130), 60.5%	-0.022 (-0.188, 0.144), 64.2%

Item S12: Results of 'leave-one-out' sensitivity analyses

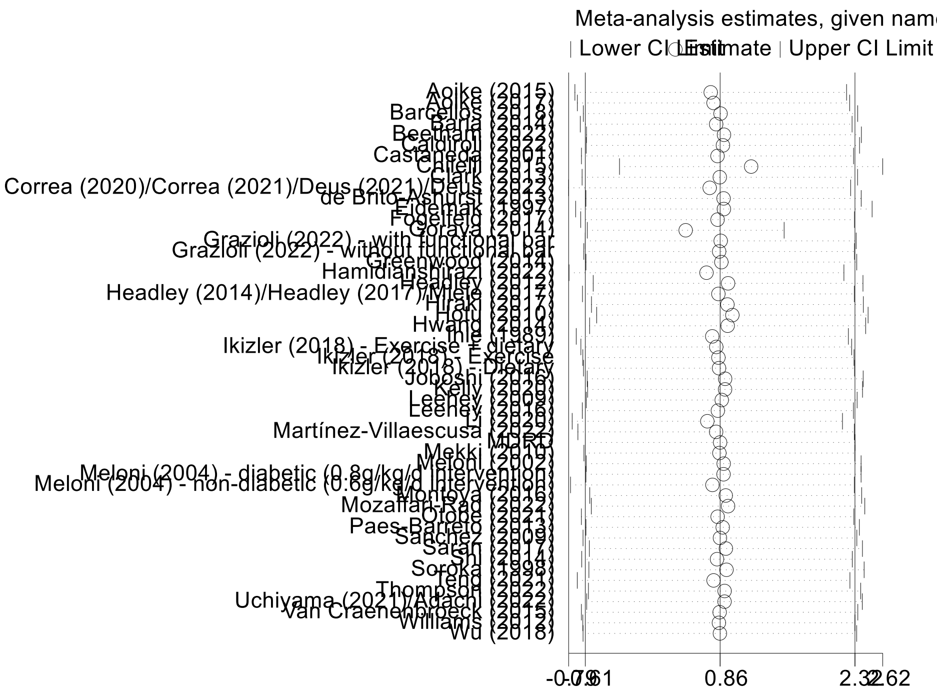

Figure 12A: Estimates for effect of lifestyle intervention on eGFR (mL/min/1.73^2) if one study was omitted

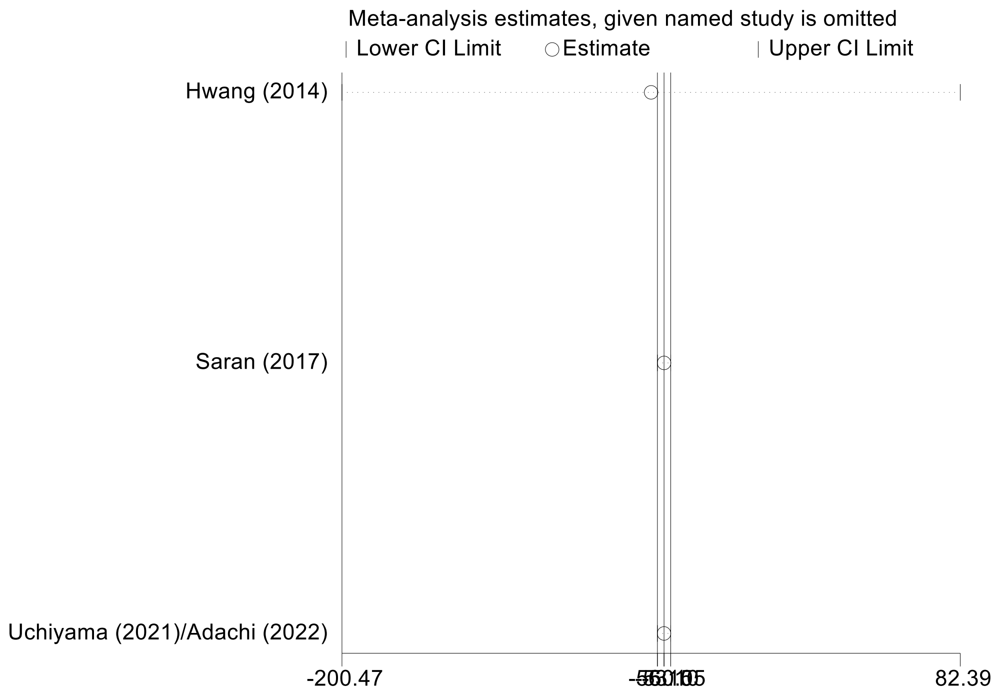

Figure 12B: Estimates for effect of lifestyle intervention on 24 hr albuminuria (mg/24 hr) if one study was omitted

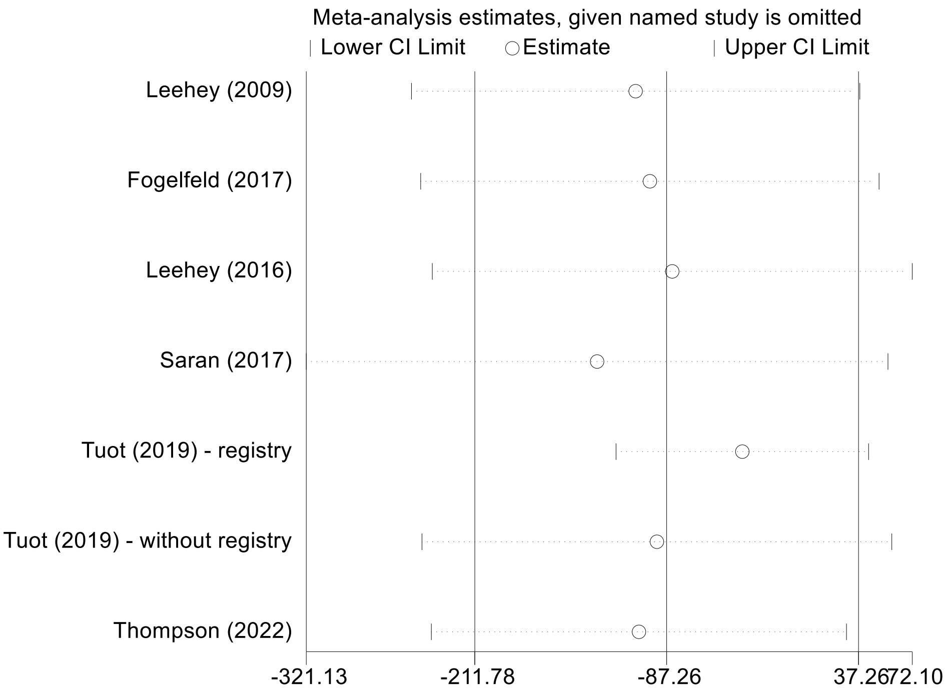

Figure S12C: Estimates for effect of lifestyle intervention on albumin: creatinine ratio (mg/g) if one study was omitted

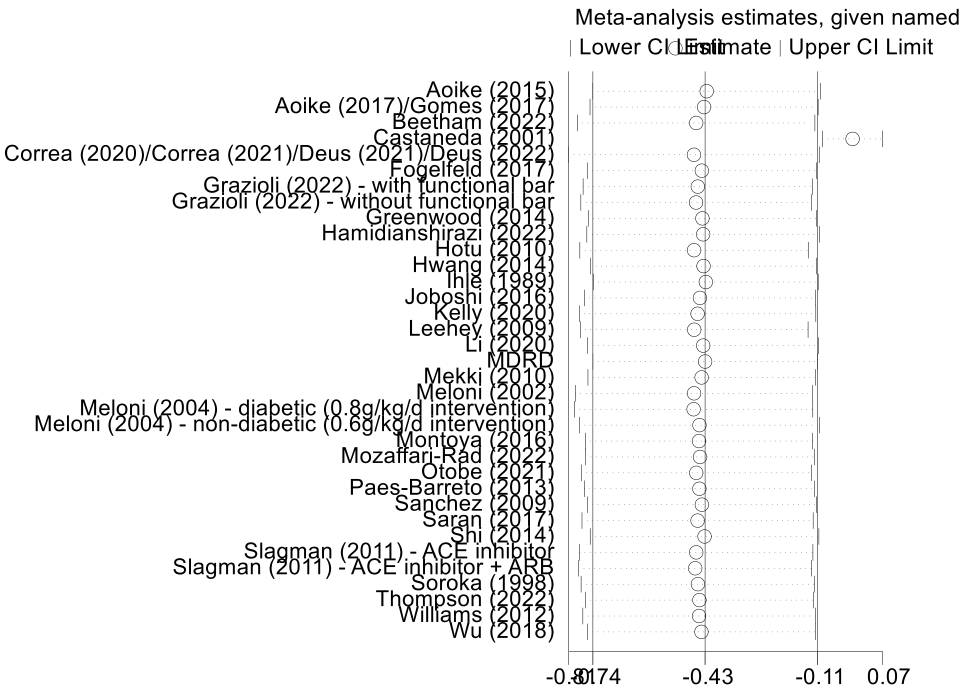

Figure 12D: Estimates for effect of lifestyle intervention on creatinine (mg/dL) if one study was omitted

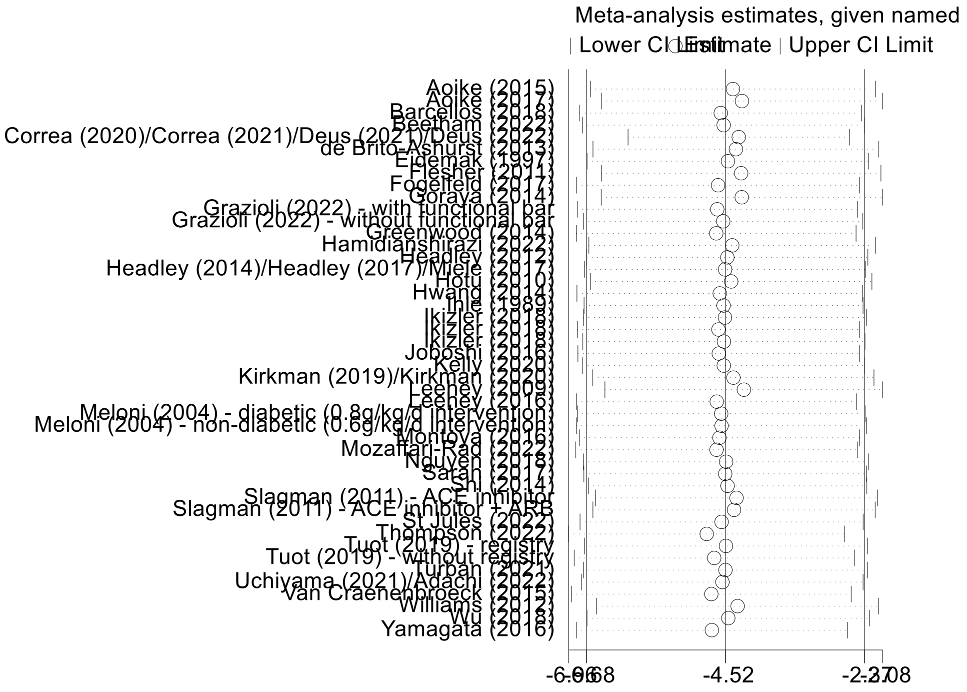

Figure 12E: Estimates for effect of lifestyle intervention on systolic blood pressure (mmHg) if one study was omitted

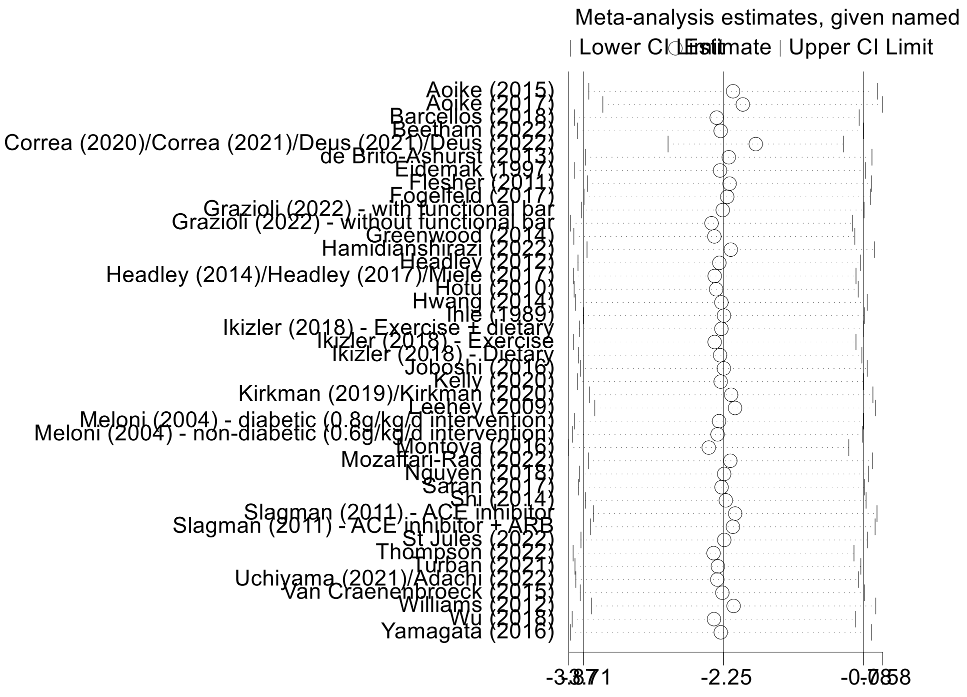

Figure 12F: Estimates for effect of lifestyle intervention on diastolic blood pressure (mmHg) if one study was omitted

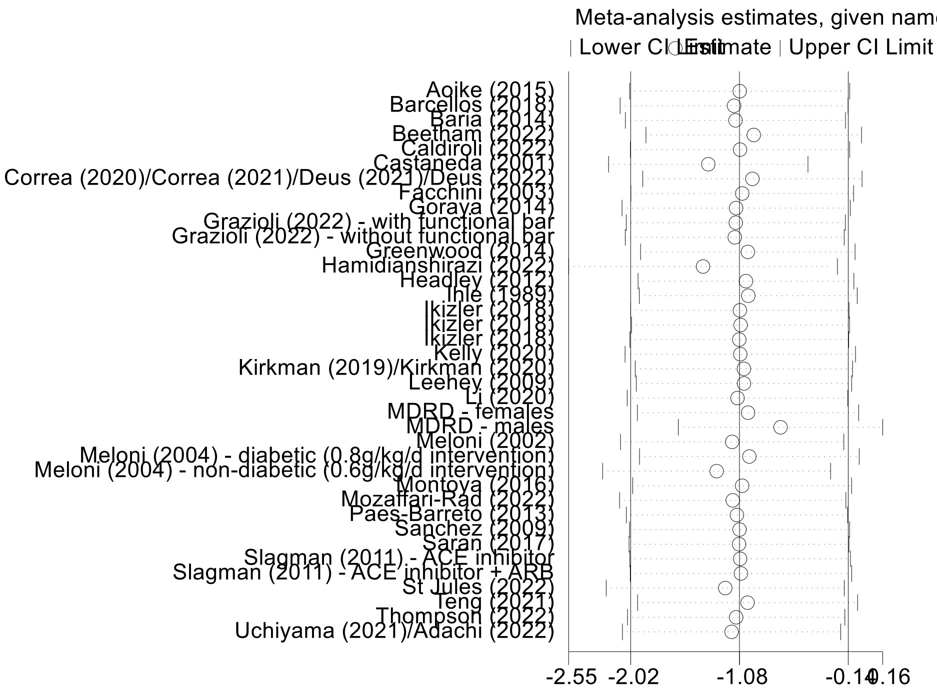

Figure 12G: Estimates for effect of lifestyle intervention on body weight (kg)

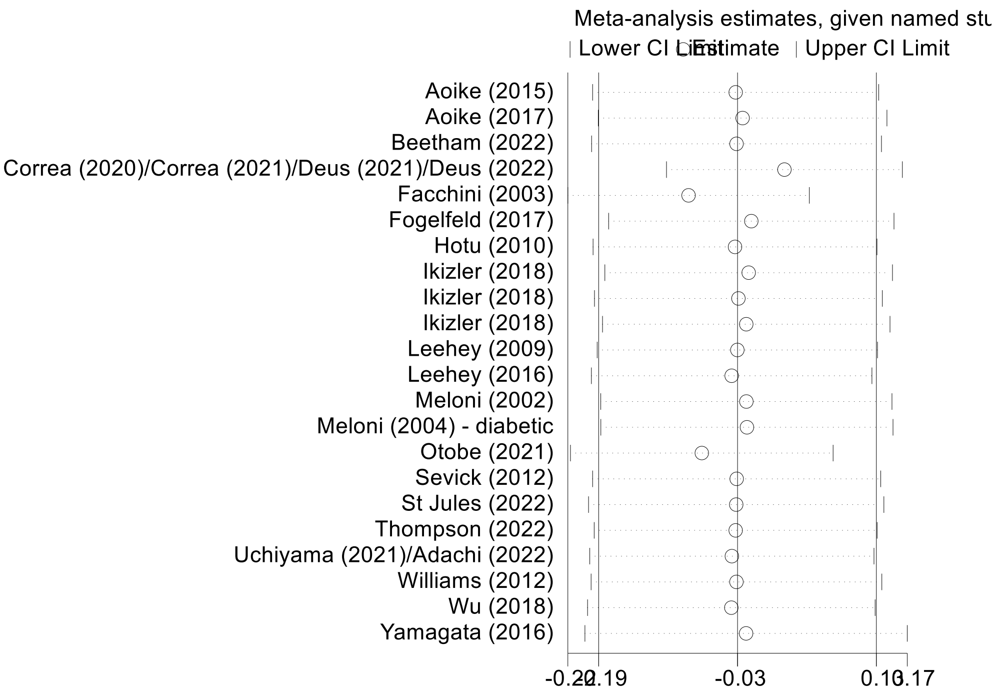

Figure 12H: Estimates for effect of lifestyle intervention on HbA1c (%)

Item S13: Contour funnel plots and results of Egger's test (limited to outcomes with more than 10 effect sizes)

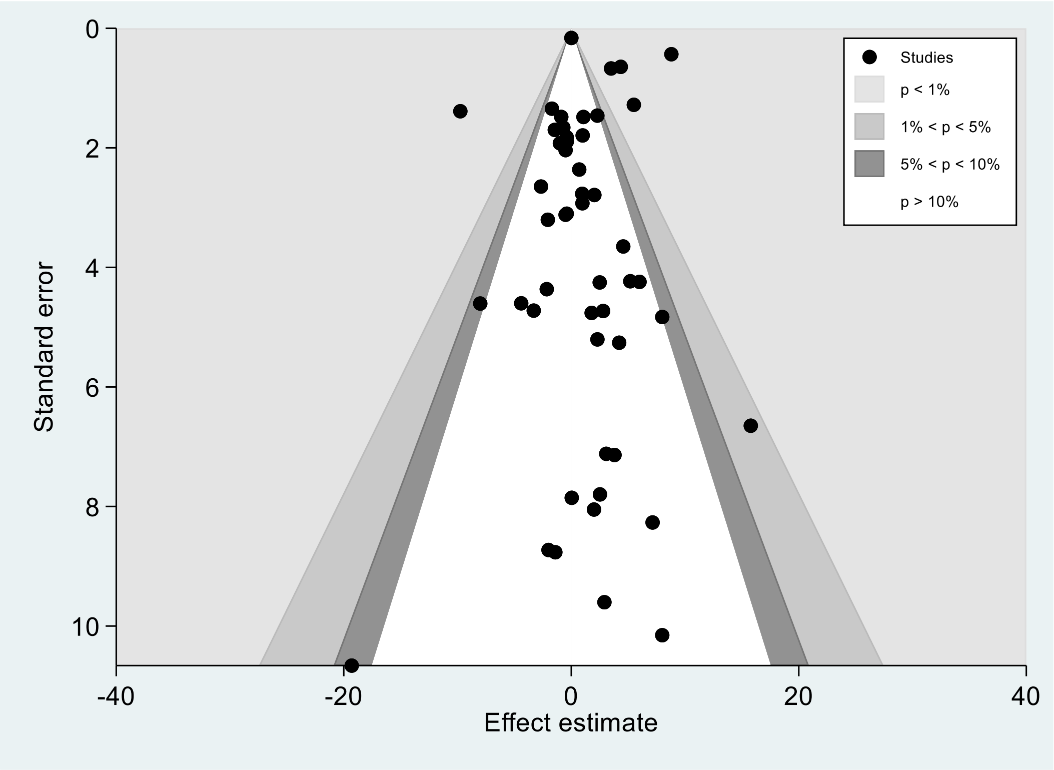

Figure 13A: Contour funnel plot for the effect of lifestyle intervention on eGFR (mL/min/1.73^2) (mg/dL)

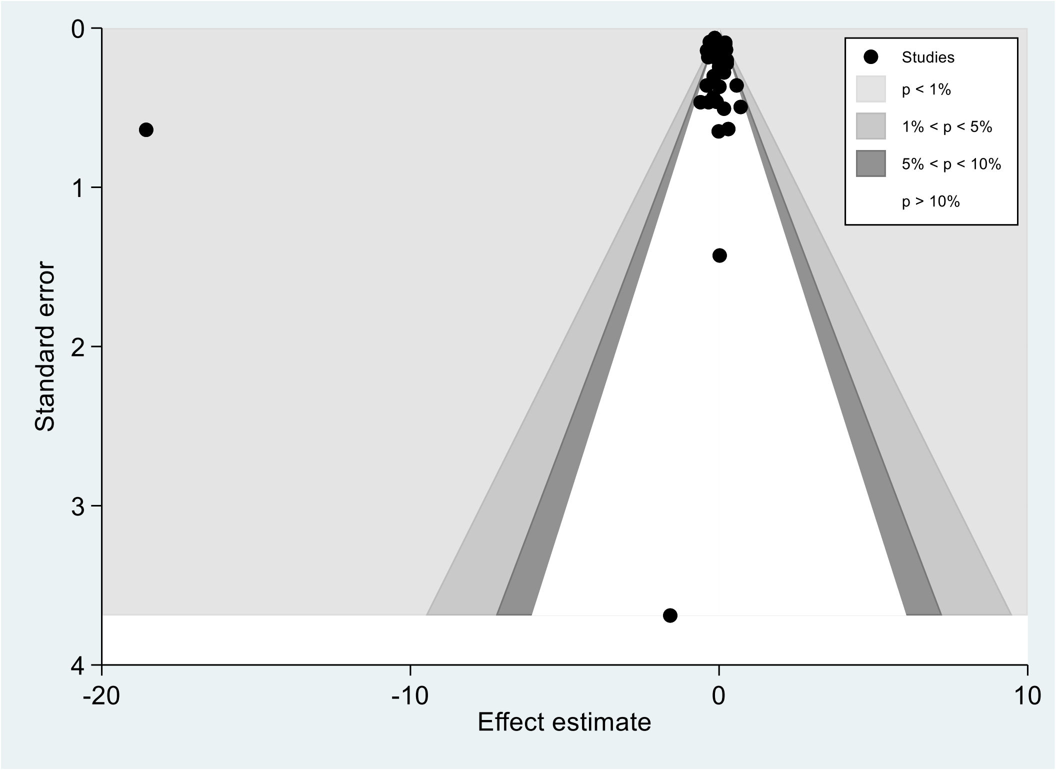

Figure 13B: Contour funnel plot of the effect of lifestyle intervention on creatinine (mg/dL)

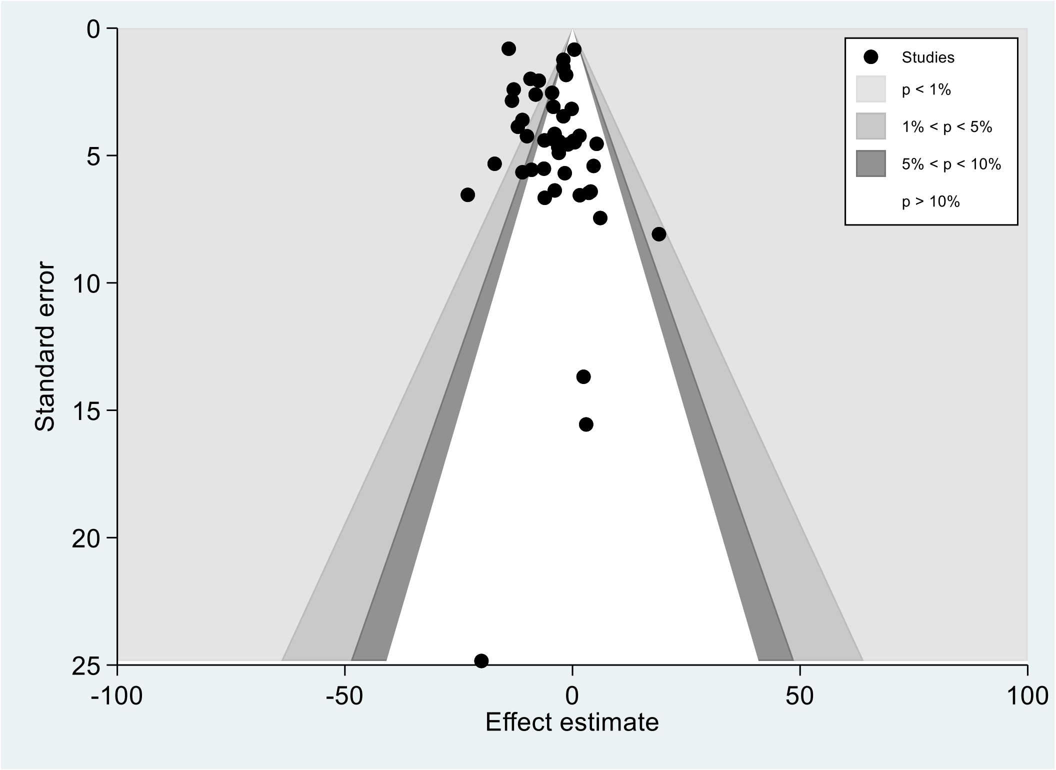

Figure 13C: Contour funnel plot of the effect of lifestyle intervention on systolic blood pressure (mmHg)

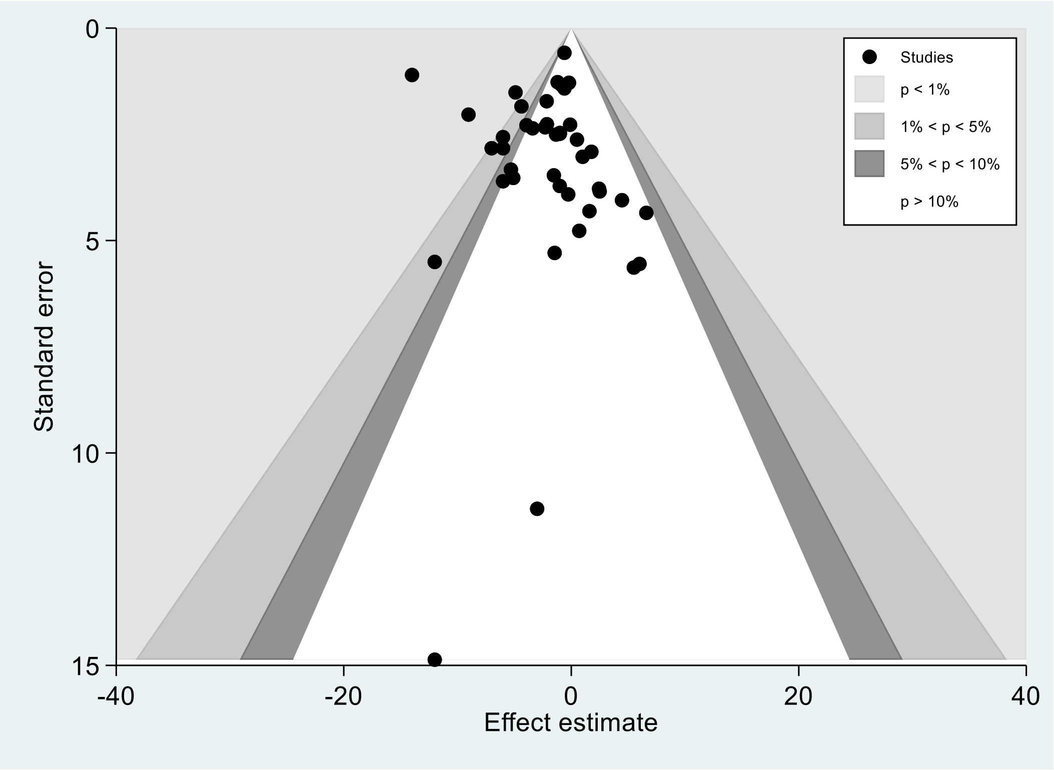

Figure 13D: Contour funnel plot of the effect of lifestyle intervention on diastolic blood pressure (mmHg)

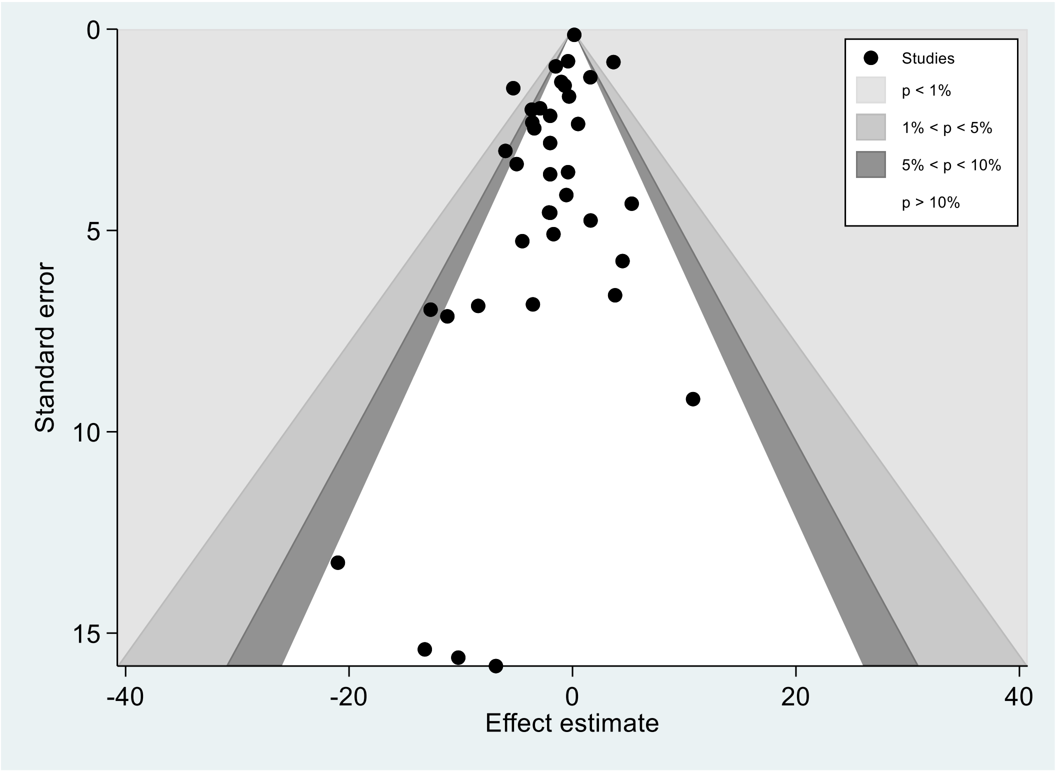

Figure 13E: Contour funnel plot of the effect of lifestyle intervention on body weight (kg)

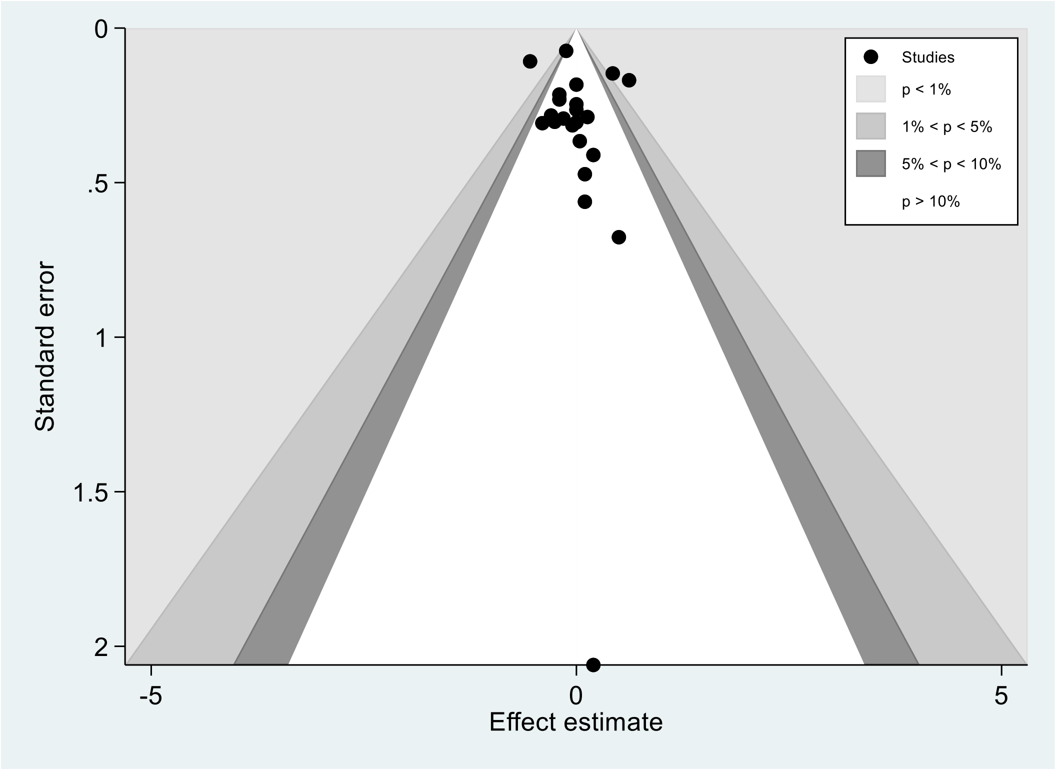

Figure 13F: Contour funnel plot of the effect of lifestyle intervention on HbA1c (%)

Table 13A: Results of Egger's test

Outcome	Bias	95% CI	p-value
**eGFR (mL/min/1.73^2)**	0.107	-0.951, 1.167	0.839
**Creatinine (mg/dL)**	-1.788	-4.877, 1.301	0.247
**Systolic blood pressure (mmHg)**	0.547	-0.557, 1.706	0.312
**Diastolic blood pressure (mmHg)**	0.186	-0.987, 1.358	0.750
**Body weight (kg)**	-0.659	-1.138, 0.180	0.008
**HbA1c (%)**	0.565	-0.731, 1.861	0.374

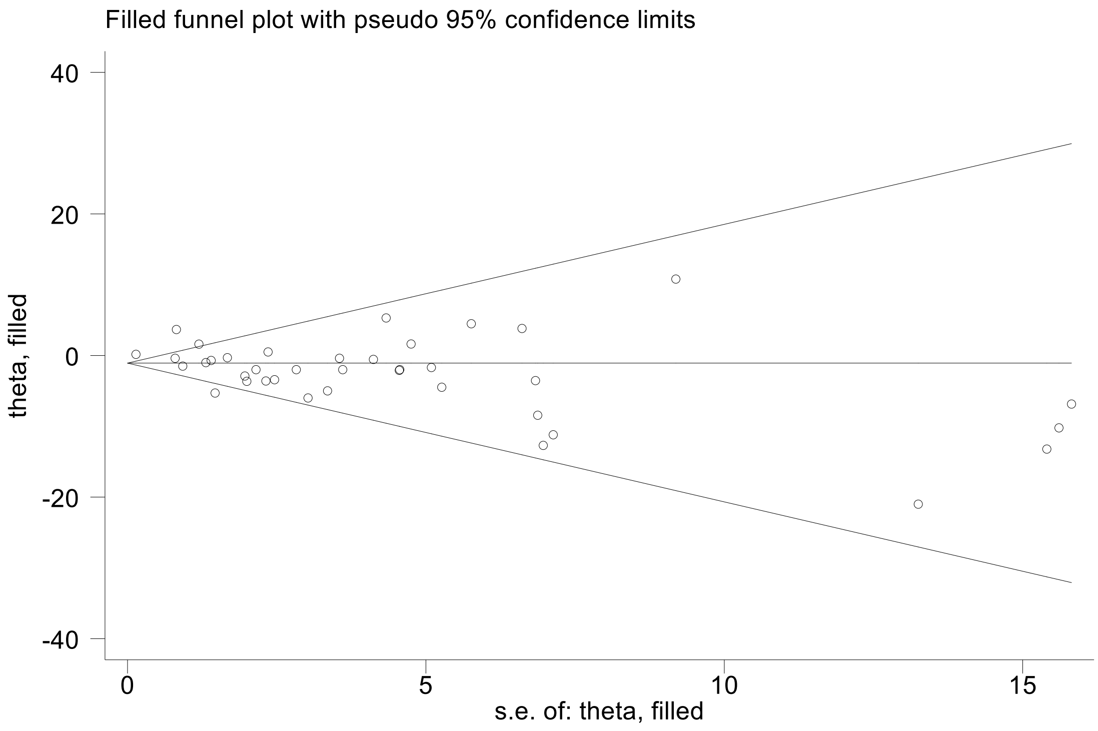

Figure 13G: Filled funnel plot for body weight

Item S14: Effect of lifestyle intervention on quality of life, compared to control

Citation	Intervention type	Tool/Domain	Significant increase	Non-significant increase	No difference	Significant decrease	Non-significant decrease
Aoike (2017)/Gomes (2017)	Exercise	SF-36 (%)	X				
Campbell (2008)	Dietary	KDQoL-SF Social support		X			
		KDQoL-SF Overall health		X			
		KDQoL-SF Effects of kidney disease		X			
		KDQoL-SF Burden of kidney disease		X			
		KDQoL-SF Work status		X			
		KDQoL-SF Cognitive function	X				
		KDQoL-SF Sleep		X			
		KDQoL-SF Satisfaction		X			
		KDQoL-SF Symptoms of kidney disease		X			
		KDQoL-SF Social interactions		X			
		SF-36 physical function	X				
		SF-36 role function		X			
		SF-36 body pain		X			
		SF-36 general health					X
		SF-36 vitality	X				

		SF-36 social function		X			
		SF-36 role emotional		X			
		SF-36 mental health					X
		SF-36 PHC		X			
		SF-36 MHC			X		
Clark (2013)	Hydration	KDQoL-SF - overall health					X
		KDQoL-SF - Affect of physical health on social functioning					X
		KDQoL-SF - sleep quality					X
		KDQoL-SF - appetite quality			X		
Hamidianshirazi (2022)	Dietary	SF-12 PCS					X
		SF-12 MCS		X			
Headley (2014)/Headley (2017)/Miele (2017)	Exercise	SF-36 - physical functioning	X				
		SF-36 - physical role functioning		X			
		SF-36 - emotional role functioning		X			
		SF-36 - vitality		X			
		SF-36 - mental health		X			
		SF-36 - social functioning		X			
		SF-36 - bodily pain	X				
		SF-36 - general health		X			

Johns (2020)	Multiple	SF-36 - composite physical score					X
		SF-36 - composite mental score		X			
Kelly (2020)	Dietary	AQoL-4D - total		X			
		AQoL-4D - independent living		X			
		AQoL-4D - relationships		X			
		AQoL-4D - senses					X
		AQoL-4D - mental health			X		
Leehey (2016)	Exercise	SF-36 PCS		X			
		SF-36 MCS		X			
Li (2020)	Multiple	KDQoL-SF - all QoL scales	X				
		KDQoL-SF - 36 item health survey scales		X			
		KDQoL-SF - general health perceptions		X			
		KDQoL-SF - physical functioning	X				
		KDQoL-SF - role-physical		X			
		KDQoL-SF - role-emotional		X			
		KDQoL-SF - Social Function		X			
		KDQoL-SF - pain		X			
		KDQoL-SF - Emotional Well-Being		X			
		KDQoL-SF - Energy/Fatigue		X			

		KDQoL-SF - Burden of kidney disease		X			
		KDQoL-SF - Cognitive function		X			
		KDQoL-SF - Symptom/problems		X			
		KDQoL-SF - Effects of kidney disease		X			
		KDQoL-SF - sleep		X			
		KDQoL-SF - Quality of social interaction		X			
		KDQoL-SF - All kidney disease–targeted scales		X			
Lin (2020)	Behaviour	WHOQOL-BREF	X				
Mustata (2011)	Exercise	EQ-5D index score		X			
		SF-36 - physical functioning					X
		SF-36 - role-physical		X			
		SF-36 - Pain		X			
		SF-36 - General health perceptions		X			
		SF-36 - Emotional well-being		X			
		SF-36 - Role-emotional		X			
		SF-36 - Social function					X
		SF-36 - Energy/fatigue		X			
		SF-36 - PCS		X			

		SF-36 - MCS		X			
Nguyen (2018)	Behaviour	SF-36 PCS	X				
		SF-36 MCS	X				
Rahimimoghadam (2018)	Exercise	KDQoL-KDCS	X				
		KDQoL-SF (Total QoL) PCS	X				
		KDQoL-SF (Total QoL) MCS	X				
		KDQoL-SF (Total QoL)	X				
Rossi (2014)	Exercise	RAND-36 (physical) - role functioning/physical	X				
		RAND-36 (physical) - physical functioning	X				
		RAND-36 (physical) - energy/fatigue	X				
		RAND-36 (physical) - general health	X				
		RAND-36 (mental) - Pain	X				
		RAND-36 (mental) - emotional well being		X			
		RAND-36 (mental) - social functioning		X			
		RAND-36 (mental) - role functioning/emotional		X			
Tang (2017)	Exercise	KDQoL-36 - symptom/problem list	X				
		KDQoL-36 - effects of kidney disease	X				

		KDQoL-36 - burden of kidney disease	X				
		SF-12 PCS	X				
		SF-12 MCS	X				
Teng (2021)	Behaviour	WHOQOL-BREF - Physical domains		X			
		WHOQOL-BREF - Psychological domains					X
		WHOQOL-BREF - Social domains		X			
		WHOQOL-BREF - Environment domains		X			
Thompson (2022)	Exercise	Veterans RAND-12 - physical health composite		X			
		Veterans RAND-12 - mental health composite		X			
Tuot (2019) - not registry	Behaviour	SF-12 - total					X
		SF-12 - physical health					X
		SF-12 - mental health					X
Tuot (2019) - registry	Behaviour	SF-12 - total		X			
		SF-12 - physical health		X			
		SF-12 - mental health		X			

Uchiyama (2021)/Adachi (2022)	Exercise	KDQOL-SF - Symptoms/problems		X			
		KDQOL-SF - Effects of kidney disease		X			
		KDQOL-SF - Burden of kidney disease					X
		KDQOL-SF - Work status	X				
		KDQOL-SF - Cognitive function		X			
		KDQOL-SF - Quality of social interaction	X				
		KDQOL-SF - Sleep		X			
		KDQOL-SF - Social support		X			
		KDQOL-SF - Overall health rating		X			
		KDQOL-SF - KDCS	X				
		SF-36 - physical functioning		X			
		SF-36 - physical role functioning		X			
		SF-36 - bodily pain					X
		SF-36 - General health		X			
		SF-36 - vitality		X			
		SF-36 - social functioning		X			
		SF-36 - Emotional role functioning		X			
		SF-36 mental health		X			

		SF-36 - PCS		X			
		SF-36 - MCS		X			
		SF-36 - RCS		X			
Van Craenenbroeck (2015)	Exercise	KDQoL-SF - Symptoms/problems		X			
		KDQoL-SF - Effects of kidney disease		X			
		KDQoL-SF - Burden of kidney disease		X			
		KDQoL-SF - Work status		X			
		KDQoL-SF - Cognitive function	X				
		KDQoL-SF - Quality of social interaction		X			
		KDQoL-SF - Sleep	X				
		KDQoL-SF - Social support		X			
		KDQoL-SF - Overall health rating		X			
		KDQoL-SF - Role–Physical		X			
		KDQoL-SF - Pain		X			
		KDQoL-SF - General Health Perceptions					X
		KDQoL-SF - Emotional Well-Being		X			

		KDQoL-SF - Role–Emotional		X			
		KDQoL-SF - Social Function		X			
		KDQoL-SF - Energy/Fatigue	X				

Abbreviations: 12-item short form survey: SF-12; 36-item short form survey: SF-36; Assessment of Quality of Life questionnaire: AQoL-4D; EuroQol five-dimensional: EQ-5D; Kidney Disease Health Related Quality of Life: KDQoL-36; Kidney Disease Health Related Quality of Life short form: KDQoL-SF; RAND 36-Item Short Form Health Survey: RAND-36; World Health Organization Quality of Life-BREF: WHOQOL-BREF

Item S15: GRADE assessment of the certainty of the body of evidence

Certainty assessment							№ of patients		Effect		Certainty	Importance
№ of studies	Study design	Risk of bias	Inconsistency	Indirectness	Imprecision	Other considerations	Lifestyle interventions	usual care	Relative (95% CI)	Absolute (95% CI)		
**Glomerular filtration rate**												
51	randomised trials	serious[a]	very serious[b]	not serious	not serious[c]	none	1479	1373	-	MD **0.857 mL/min/1.73^2 higher** (0.606 lower to 2.320 higher)	⊕◯◯◯ Very low	CRITICAL
**Albumin:creatinine ratio**												
7	randomised trials	very serious[d]	not serious	not serious	serious[e]	none	197	192	-	MD **87.261 mg/g lower** (211.778 lower to 37.255 higher)	⊕◯◯◯ Very low	CRITICAL
**24 hour albuminuria**												
3	randomised trials	not serious	not serious	not serious	serious[f]	none	170	176	-	MD **53.103 mg/24 hr lower** (56.151 lower to 50.054 lower)	⊕⊕⊕◯ Moderate	CRITICAL
**Creatinine**												
34	randomised trials	serious[g]	very serious[h]	not serious	not serious	none	1072	1058	-	MD **0.426 mg/dL lower** (0.740 lower to 0.111 lower)	⊕◯◯◯ Very low	CRITICAL
**Systolic blood pressure**												
46	randomised trials	serious[i]	very serious[j]	not serious	not serious	none	1472	1377	-	MD **4.524 mmHg lower** (6.683 lower to 2.365 lower)	⊕◯◯◯ Very low	CRITICAL
**Diastolic blood pressure**												
42	randomised trials	serious[k]	very serious[l]	not serious	not serious	none	1351	1263	-	MD **2.247 mmHg lower** (3.713 lower to 0.781 lower)	⊕◯◯◯ Very low	CRITICAL
**Body weight**												
38	randomised trials	serious[o]	serious[p]	not serious	not serious	publication bias strongly suspected[q]	1386	1275	-	MD **1.076 kg lower** (2.016 lower to 0.136 lower)	⊕◯◯◯ Very low	IMPORTANT

Certainty assessment							№ of patients		Effect		Certainty	Importance
№ of studies	Study design	Risk of bias	Inconsistency	Indirectness	Imprecision	Other considerations	Lifestyle interventions	usual care	Relative (95% CI)	Absolute (95% CI)		
**HbA1c**												
22	randomised trials	serious[r]	serious[s]	not serious	not serious	none	765	682	-	MD **0.030 % lower** (0.189 lower to 0.130 higher)	⨁⨁◯◯ Low	IMPORTANT
**Quality of Life**												
21	randomised trials	serious[s]	not serious	not serious	not serious	none	680	646	-	Not pooled	⨁⨁⨁◯ Moderate	IMPORTANT

CI: confidence interval; **MD:** mean difference

Explanations

a. The studies were viewed as being in the category of ' serious limitation'. This category was selected as the risk of bias assessments for each study resulted in mainly 'unknown risk' (see risk of bias assessment charts). In accordance with the GRADE guidelines, 'unclear risk' needed to be categorised as either 'no limitations' or 'serious limitations'. In view of the potential implications of the 'unclear risk' aspects on the certainty of the body of evidence, 'serious limitations' was selected

b. I squared value of 90.2% indicating considerable heterogeneity

c. Sample size in review exceeds estimated Optimal Information Size of 400 pts. 95% confidence intervals in analysis do not cross appreciable harm/benefit and no effect. As a result, this outcome was not downgraded for imprecision

d. The studies were viewed as being in the category of ' very serious limitation'. This category was selected as the risk of bias assessments for each study resulted in mainly 'high risk' (see risk of bias assessment charts). In view of the potential implications of the 'high risk' aspects on the certainty of the body of evidence, 'very serious limitations' was selected

e. Sample size in review does not meet estimated Optimal Information Size of 400 pts.

f. Sample size in review does not meet estimated Optimal Information Size of 400 pts.

g. The studies were viewed as being in the category of ' serious limitation'. This category was selected as the risk of bias assessments for each study resulted in mainly 'unknown risk' (see risk of bias assessment charts). In view of the potential implications of the 'unclear risk' aspects on the certainty of the body of evidence, 'serious limitations' was selected

h. I squared value of 96.3% indicating considerable heterogeneity

i. The studies were viewed as being in the category of ' serious limitation'. This category was selected as the risk of bias assessments for each study resulted in mainly 'unknown risk' (see risk of bias assessment charts). In view of the potential implications of the 'unclear risk' aspects on the certainty of the body of evidence, 'serious limitations' was selected

j. I squared value of 82.7% indicating considerable heterogeneity

k. The studies were viewed as being in the category of ' serious limitation'. This category was selected as the risk of bias assessments for each study resulted in mainly 'unknown risk' (see risk of bias assessment charts). In view of the potential implications of the 'unclear risk' aspects on the certainty of the body of evidence, 'serious limitations' was selected

l. I squared value of 76.8% indicating considerable heterogeneity

o. The studies were viewed as being in the category of ' serious limitation'. This category was selected as the risk of bias assessments for each study resulted in mainly 'unknown risk' (see risk of bias assessment charts). In view of the potential implications of the 'unclear risk' aspects on the certainty of the body of evidence, 'serious limitations' was selected

p. I squared value of 50.1% indicating moderate heterogeneity

q. Funnel plot and results of Egger's test suggest small study effects which may be attributable to publication bias

r. The studies were viewed as being in the category of ' serious limitation'. This category was selected as the risk of bias assessments for each study resulted in mainly 'unknown risk' (see risk of bias assessment charts). In view of the potential implications of the 'unclear risk' aspects on the certainty of the body of evidence, 'serious limitations' was selected

s. I squared value of 60.5% indicating substantial heterogeneity

t. The studies were viewed as being in the category of ' serious limitation'. This category was selected as the risk of bias assessments for each study resulted in mainly 'unknown risk' (see risk of bias assessment charts). In view of the potential implications of the 'unclear risk' aspects on the certainty of the body of evidence, 'serious limitations' was selected

References

1. Rohatgi A. WebPlotDigitizer version 4.5. https://automeris.io/WebPlotDigitizer
2. Higgins J, Thomas, J, Chandler, J, Cumpston, M, Li, T, Page, MJ, Welch, VA,. Cochrane Handbook for Systematic Reviews of Interventions version 6.3 (updated February 2022). Cochrane. www.training.cochrane.org/handbook
3. Wan X, Wang W, Liu J, Tong T. Estimating the sample mean and standard deviation from the sample size, median, range and/or interquartile range. *BMC medical research methodology*. 2014;14(1):1-13.
4. Luo D, Wan X, Liu J, Tong T. Optimally estimating the sample mean from the sample size, median, mid-range, and/or mid-quartile range. *Statistical methods in medical research*. 2018;27(6):1785-1805.
5. Williams A, Manias E, Walker R, Gorelik A. A multifactorial intervention to improve blood pressure control in co-existing diabetes and kidney disease: a feasibility randomized controlled trial. *Journal of advanced nursing*. 2012;68(11):2515-2525.
6. Neale EP, Guan V, Tapsell LC, Probst YC. Effect of walnut consumption on markers of blood glucose control: A systematic review and meta-analysis. *British Journal of Nutrition*. 2020;124(7):641-653.
7. Neale EP, Tapsell LC, Guan V, Batterham MJ. The effect of nut consumption on markers of inflammation and endothelial function: a systematic review and meta-analysis of randomised controlled trials. *BMJ open*. 2017;7(11):e016863.
8. Ikizler TA, Robinson-Cohen C, Ellis C, et al. Metabolic effects of diet and exercise in patients with moderate to severe CKD: a randomized clinical trial. *Journal of the American Society of Nephrology*. 2018;29(1):250-259.
9. Tangri N, Stevens LA, Schmid CH, et al. Changes in dietary protein intake has no effect on serum cystatin C levels independent of the glomerular filtration rate. *Kidney international*. 2011;79(4):471-477.
